# A synoptic review of the aloes (Asphodelaceae, Alooideae) of KwaZulu-Natal, an ecologically diverse province in eastern South Africa

**DOI:** 10.3897/phytokeys.142.48365

**Published:** 2020-03-12

**Authors:** Ronell R. Klopper, Neil R. Crouch, Gideon F. Smith, Abraham E. van Wyk

**Affiliations:** 1 Biosystematics and Biodiversity Collections Division, South African National Biodiversity Institute, Private Bag X101, Pretoria, 0002 South Africa South African National Biodiversity Institute Pretoria South Africa; 2 H.G.W.J. Schweickerdt Herbarium, Department of Plant and Soil Sciences, University of Pretoria, Pretoria, 0002 South Africa University of Pretoria Pretoria South Africa; 3 Biodiversity Research, Monitoring and Assessment, South African National Biodiversity Institute, P.O. Box 52099, Berea Road, 4007 South Africa South African National Biodiversity Institute Durban South Africa; 4 School of Chemistry & Physics, University of KwaZulu-Natal, Durban, 4041 South Africa University of KwaZulu-Natal Durban South Africa; 5 Department of Botany, Nelson Mandela University, P.O. Box 77000, Port Elizabeth, 6031 South Africa Nelson Mandela University Port Elizabeth South Africa

**Keywords:** *
Aloe
*, *
Aloiampelos
*, *
Aloidendron
*, *
Aristaloe
*, conservation status, distribution map, Maputaland-Pondoland-Albany Hotspot

## Abstract

The KwaZulu-Natal province of South Africa has a varied topography, geology and climate and presents diverse habitats that support a rich and diverse flora. Aloes are well represented in KwaZulu-Natal, with four genera [*Aloe* L., *Aloiampelos* Klopper & Gideon F.Sm., *Aloidendron* (A.Berger) Klopper & Gideon F.Sm. and *Aristaloe* Boatwr. & J.C.Manning] and 49 taxa occurring in the province. Fourteen of these are endemic and eleven near-endemic to the province. A floristic treatment of the aloes of KwaZulu-Natal is presented in the form of a synoptic review. Included are an identification key to the aloes that occur naturally in the province, species-level distribution maps and accompanying images, so providing for the first time, an atlas of aloe occurrence in this part of the subcontinent.

## Introduction

The KwaZulu-Natal province covers an area of ± 92 290 km^2^ in the southeast of South Africa (Raimondo et al. 2009). It has a ± 580 km long coastline (Palmer et al. 2011) with the warm Agulhas Current of the Indian Ocean in the east and is bordered in the south and southwest by the Eastern Cape province, in the west by the Free State province and Lesotho and, in the north, by the Mpumalanga province, Eswatini (formerly Swaziland) and Mozambique.

The main agricultural industry in KwaZulu-Natal is sugar production and vast areas of the province are covered with sugar cane plantations. Other agricultural activities include farming with sheep, cattle (dairy and beef), plantation forestry, citrus fruit, maize, sorghum, cotton, bananas, macadamia nuts and pineapples. Industrial areas with textile, chemical, vehicle and food-processing plants and oil refineries are mainly located near the main ports of Durban (east-central KwaZulu-Natal) and Richards Bay (north coast). Coastal dune mining for heavy metals and minerals is having a negative impact on the coastal dune vegetation and marine ecology along parts of the north coast of KwaZulu-Natal. The province is also a popular tourism destination, especially the coastal region and the high mountains of the Great Escarpment (Drakensberg) on the border with Lesotho (Palmer et al. 2011; http://en.wikipedia.org/wiki/KwaZulu-Natal).

Many areas in KwaZulu-Natal are densely populated or otherwise anthropogenically impacted, leading to the destruction or degradation of much natural vegetation. By 2005, 43% of the natural habitat in the province was already transformed, with the rate of such change much higher than the national average (Jewitt et al. 2015). These authors demonstrated that, in the seven-year period 2005–2011, an alarming 7.6% more natural habit was lost to anthropogenic transformation of the landscape. Jewitt et al. (2015) determined that the main drivers of change in the KwaZulu-Natal landscape were agriculture, silviculture (plantation timber industry), built environments (peri-urban expansion), mines and dam construction. Unsustainable farming practices, the cultivation of monocultures such as sugar cane, commercial timber plantations and dune mining have long been recognised as important drivers of habitat loss in the province (Van Wyk and Smith 2001; Steenkamp et al. 2004). The province includes two natural areas that have been declared UNESCO World Heritage Sites, namely the iSimangaliso Wetland Park and the uKhahlamba Drakensberg Park, where sensitive ecosystems are protected. Several other smaller protected areas are scattered throughout the province. These areas play a vital role in conserving natural habitats and the species they harbour and, concomitantly, service the ecotourism economy of the province (Cyrus and Robson 1980; http://en.wikipedia.org/wiki/KwaZulu-Natal).

The climate of KwaZulu-Natal ranges from temperate in the higher inland areas to subtropical or tropical along the coast. Maputaland, in the north-eastern corner of KwaZulu-Natal, is at the southern end of the tropics in Africa and many tropical plants and animals reach the southermost limit of their range here (Van Wyk 1996). The southern boundary of this true tropical area seems to follow the 18 °C mean midwinter isotherm (Poynton 1961, 1962). Mean summer temperatures in the high Drakensberg are below 22 °C (Van Wyk and Smith 2001) and in the low 20s to high 30s at the coast (Palmer et al. 2011). Coastal temperatures (°C) range from the low teens to mid-20s in winter (Palmer et al. 2011), while very low temperatures of below freezing with frost and snow are regularly recorded during winter along the Drakensberg Escarpment (Van Wyk and Smith 2001). Mean daily winter temperatures in the mid-teens are usually experienced throughout the midlands (Cyrus and Robson 1980). KwaZulu-Natal receives predominantly summer rain (November–March) and the relative humidity is usually high, especially near and along the coast. Mist and fog is fairly common in the midlands (Van Wyk and Smith 2001). Rainfall at the coast can be as high as 1 200–1 500 mm per annum (McDonald and Jarman 1985; Palmer et al. 2011), but is lower at inland localities, with as little as 650 mm in the north-eastern interior and some of the drier river valleys (McDonald and Jarman 1985). Mean annual rainfall along the high Drakensberg Escarpment can be as high as 2 000 mm (Van Wyk and Smith 2001).

Elevation ranges from sea level at the coast to an average crest height on the Drakensberg Escarpment, on the border between Lesotho and KwaZulu-Natal, of around 3 377 m above sea level (a.s.l.) (McDonald and Jarman 1985; Van Wyk and Smith 2001). Soils in KwaZulu-Natal were formed from a variety of geological formations and under various climatic and topographical conditions. This leads to a very diverse soil profile throughout the province (McDonald and Jarman 1985).

**Figure 1.**
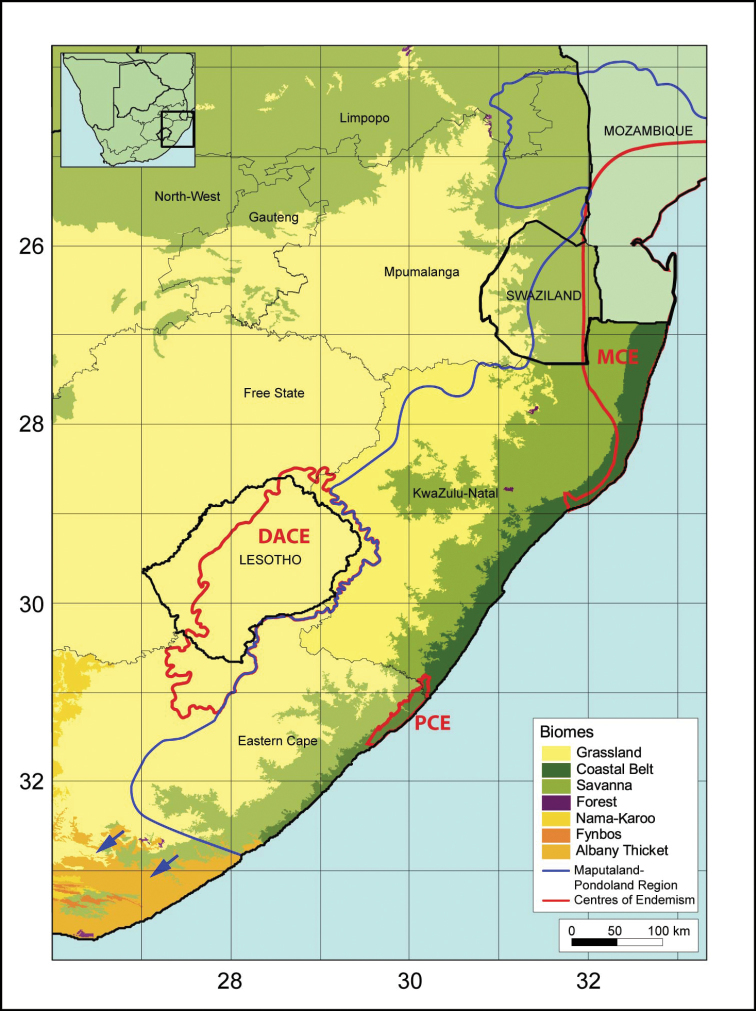
Vegetation of the KwaZulu-Natal province of South Africa (based on Mucina and Rutherford 2006), the boundary of the Maputaland-Pondoland Region and the Maputaland (MCE), Pondoland (PCE) and Drakensberg Alpine (DACE) Centres of Endemism (based on Van Wyk and Smith 2001).

The great variation found in climate, topography and geology throughout KwaZulu-Natal leads to high environmental heterogeneity. In addition, the proximity of the warm Agulhas Current may well have provided some climatic stability by acting as a buffer against periods of cooler climates in the past. This in turn has led to, inter alia, an exceptionally diverse vegetation and flora in the province (Ross 1972). KwaZulu-Natal harbours a large number of vegetation types (Mucina and Rutherford 2006) (see Fig. 1). Grassland predominates in the west of the province with savannah towards the east. Alpine vegetation occurs on the high Drakensberg Escarpment on the border with Lesotho. The Coastal Belt vegetation is regularly interrupted by large river systems, where a thicket-type vegetation is often present in the valleys. Numerous relatively small (relict) pockets of forest occur throughout the province and large wetlands are present, especially in the northeast (Maputaland; e.g. iSimangaliso) (Van Wyk and Smith 2001; Mucina 2018). KwaZulu-Natal harbours ± 5 250 indigenous plant species and infraspecific taxa. After the Eastern Cape (with ± 6 070 taxa), this is the second most diverse flora for any of the South African provinces. Around 390 species and infraspecific taxa (7.5%) are endemic to the province and ± 470 (9%) are regarded as taxa being of conservation concern (217 of which are threatened with extinction according to IUCN Red List criteria) (Raimondo and Von Staden 2009).

One of the 34 global biodiversity hotspots recognised by Conservation International (Mittermeier et al. 2004), namely, the Maputaland-Pondoland-Albany Hotspot (Steenkamp et al. 2004) covers most of KwaZulu-Natal. In addition, three major local centres of plant endemism fall partly in the province (Van Wyk and Smith 2001). These are the Maputaland, Pondoland and Drakensberg Alpine Centres of Endemism. Almost the entire KwaZulu-Natal is included in the Maputaland-Pondoland Region (sensu Van Wyk and Smith 2001; partly congruent with and representing the bulk of the Maputaland-Pondoland-Albany Hotspot), a floristic unit that is recognised at a higher level and includes the three above-mentioned Centres of Endemism and other smaller local centres of endemism (see Fig. 1). In terms of species numbers, the Maputaland-Pondoland Region is, after the Cape Floristic Region, the second-richest floristic region in southern Africa (Van Wyk and Smith 2001). This Region also extends northwards to around the Hoedspruit area in South Africa’s Mpumalanga province, Massingir and Xai-Xai in Mozambique and in a southwesterly direction into the Eastern Cape province, to beyond East London. This entire region has an endemism level of around 25.7% and also harbours a large number of threatened plants (Van Wyk and Smith 2001). The majority of endemics are confined to the most threatened vegetation type in the region, namely the Grassland Biome (Van Wyk and Smith 2001; Steenkamp et al. 2004).

The Drakensberg Alpine Centre of Endemism covers the central high-lying portion of the Drakensberg Mountains in Lesotho and western KwaZulu-Natal (see Fig. 1). The high Drakensberg (above 1 800 m a.s.l.) is often recognised as a distinct floristic region, based on climatological and floristic evidence and the vegetation can be broadly classified into subalpine (± 1 800–2 800 m) and alpine belts (± 2 800–3 500 m) (Killick 1978; Van Wyk and Smith 2001). Levels of endemism of 13% for strict endemics and 37% for near-endemic taxa have been recorded for the Drakensberg Alpine Centre (Carbutt and Edwards 2006), also referred to as the Drakensberg Mountain Centre (Carbutt 2019), although endemic succulents are generally not well-represented in this region (e.g. see Smith and Willis 1999 on *Crassula* L.). The only aloe that is endemic to the Drakensberg Alpine Centre is the highly threatened spiral aloe, *Aloe polyphylla* Schönland ex Pillans (Van Wyk and Smith 2001). Note, however, that as *Aloe polyphylla* is confined to Lesotho, it is not treated in this atlas for the aloes of KwaZulu-Natal.

## KwaZulu-Natal aloes

South Africa harbours 27% (± 170 species and infraspecific taxa) of the world’s ± 630 species of aloe. This is more than for any other country. KwaZulu-Natal alone has 49 aloes (29% of the aloes in South Africa). Of these, 14 (28.5%) are endemic to the province and a further 11 (22%) near-endemic. Near-endemic status is here applied to restricted range aloes where more than 75% of the distribution range falls within KwaZulu-Natal. Accordingly, a majority (51%) of the aloes of KwaZulu-Natal are wholly or mostly restricted to the province. At least 31 aloes in KwaZulu-Natal (63%) are endemic or near-endemic to the Maputaland-Pondoland Region (see Table 1). As a result, figures for aloe endemism are much higher than the 7.5% endemism estimated for the province or the average 25.7% endemism recorded for the flora of the Maputaland-Pondoland Region, making KwaZulu-Natal an important area for the conservation of aloes and their habitats.

**Table 1.** Checklist of endemic and near-endemic aloe taxa of KwaZulu-Natal, South Africa and the Maputaland-Pondoland Region. Restricted range aloes, where more than 75% of the distribution range falls within KwaZulu-Natal, are here regarded as near-endemic to the province. Also indicated are taxa that only have a minor part of their range in this province, but have their main distribution range outside of KwaZulu-Natal, as well as taxa that reach the limits of their distribution in this province. [N – reaches northern extreme of range; NE – reaches northeast of range; S – reaches southern extreme of range; SE – reaches south-eastern extreme of range; MPR – Maputaland-Pondoland Region endemic or near-endemic].

Taxon	Endemic	Near-endemic	Minor range	Limit	MPR
*Aloiampelos tenuior*			X	N	
*Aloidendron barberae*					X
*Aloidendron tongaense*			X	S	X
*Aristaloe aristata*			X	NE	
*Aloe arborescens*					
*Aloe bergeriana*			X	SE	
*Aloe boylei*					
*Aloe candelabrum*	X				X
Aloe chabaudii var. chabaudii			X	S	
*Aloe cooperi*					X
*Aloe dewetii*		X			X
*Aloe dominella*		X			X
*Aloe ecklonis*			X		
*Aloe gerstneri*	X				X
*Aloe hlangapies*		X			X
*Aloe inconspicua*	X				X
*Aloe kniphofioides*			X	S	
*Aloe kraussii*	X				X
*Aloe linearifolia*		X			X
Aloe maculata subsp. maculata					
Aloe marlothii subsp. marlothii				S	
Aloe marlothii subsp. orientalis				S	X
*Aloe micracantha*			X	N	
*Aloe minima*					X
*Aloe modesta*				S	
*Aloe mudenensis*		X			X
*Aloe myriacantha*					
*Aloe neilcrouchii*	X				X
*Aloe nicholsii*	X				X
*Aloe parvibracteata*				S	
*Aloe parviflora*	X				X
*Aloe pluridens*			X	N	
*Aloe pratensis*			X	N	
*Aloe prinslooi*	X				X
*Aloe pruinosa*	X				X
Aloe reitzii var. vernalis	X				X
*Aloe rupestris*		X			X
*Aloe saundersiae*	X				X
*Aloe sharoniae*		X			X
*Aloe spectabilis*	X				X
*Aloe spicata*			X	S	
*Aloe suffulta*				S	X
*Aloe suprafoliata*		X			X
*Aloe thraskii*		X			X
*Aloe umfoloziensis*		X			X
*Aloe vanbalenii*		X			X
*Aloe vanrooyenii*	X				X
*Aloe viridiana*	X				X
*Aloe vryheidensis*			X	S	
**Total = 49**	**14**	**11**	**12**	**16**	**31**

A total of 12 (24.5%) aloes have the largest part of their distribution ranges outside of KwaZulu-Natal and only marginally enter this province. The distribution range of eight (16%) aloes extend to the north and south of KwaZulu-Natal, while 16 of the non-endemic aloes (33%) reach either the southern or northern limit of their distribution ranges within the province (see Table 1).

Aloes in KwaZulu-Natal are represented by four genera (generic classification following Grace et al. 2013 and Manning et al. 2014): one scrambling aloe in the genus *Aloiampelos* Klopper & Gideon F.Sm., two tree aloes in the genus *Aloidendron* (A.Berger) Klopper & Gideon F.Sm., the monotypic *Aristaloe* Boatwr. & J.C.Manning and 45 “true aloes” in the genus *Aloe*, including one member of Aloe section Chortolirion (A.Berger) Boatwr. & J.C.Manning (previously treated at genus rank as *Chortolorion* A.Berger).

KwaZulu-Natal is especially rich in so-called grass aloes with no less than 18 species (37% of the aloes treated here) (Craib 2005). This is around 55% of the total number of grass aloes present in South Africa. Ten of these (55% of grass aloes in KwaZulu-Natal) are either endemic (six; 33%) or near-endemic (four; 22%) to the province (see Table 1). Considering that the Grassland Biome, which is the habitat of these grass aloes, is the most threatened vegetation type in the region (Van Wyk and Smith 2001; Steenkamp et al. 2004), the importance of grassland conservation in KwaZulu-Natal for the continued survival of these aloes cannot be over-emphasised. This is further highlighted by the fact that six (33%) of the grass aloes in KwaZulu-Natal are regarded as threatened (see Table 2).

Another group that is very well represented in this province is the maculate aloes, with ten (20% of the aloes in KwaZulu-Natal) representatives, of which four (40% of maculate aloes in KwaZulu-Natal) are endemic and three (30%) near-endemic (see Table 1). However, there is considerable debate on the delimitation of *Aloe* taxa in northern KwaZulu-Natal, especially regarding the maculate aloes. In this region, several species grow sympatrically and hybridisation is common amongst species with overlapping flowering periods, with the result that, at certain localities, species seem to intergrade. This makes identification difficult and in several areas plants can only be positively identified during the flowering season. A particular example is the *Aloe maculata* / *Aloe parvibracteata*-complex of maculate aloes that flower in mid-winter (southern hemisphere), during June and July. A gradual cline has been observed from south to north amongst *Aloe maculata* All., *Aloe umfoloziensis* Reynolds and *Aloe parvibracteata* Schönland (Reynolds 1950). At their geographical extremes, it is, however, possible to tell these three species apart.

This synoptic review provides a complete floristic treatment of the aloes of KwaZulu-Natal. It also contains an identification key to the aloes of this province, along with species-level distribution maps and accompanying images, giving for the first time, an atlas of aloe occurrence in this part of the subcontinent. To prevent confusion between *Aloe*, *Aloiampelos* and *Aristaloe* in the species treatments, we do not abbreviate generic names to the first letter.

**Table 2.** Red-List categories for the aloe taxa in KwaZulu-Natal. [Least Concern (LC), Near-threatened (NT), Vulnerable (VU), Endangered (EN), Critically Endangered (CR); Taxa of conservation concern = NT, VU, EN, CR; Threatened taxa = VU, EN, CR].

Taxon	LC	NT	VU	EN	CR
*Aloiampelos tenuior*	X				
*Aloidendron barberae*	X				
*Aloidendron tongaense*	X				
*Aristaloe aristata*	X				
*Aloe arborescens*	X				
*Aloe bergeriana*	X				
*Aloe boylei*	X				
*Aloe candelabrum*		X			
Aloe chabaudii var. chabaudii	X				
*Aloe cooperi*	X				
*Aloe dewetii*	X				
*Aloe dominella*		X			
*Aloe ecklonis*	X				
*Aloe gerstneri*				X	
*Aloe hlangapies*			X		
*Aloe inconspicua*				X	
*Aloe kniphofioides*		X			
*Aloe kraussii*				X	
*Aloe linearifolia*	X				
Aloe maculata subsp. maculata	X				
Aloe marlothii subsp. marlothii	X				
Aloe marlothii subsp. orientalis	X				
*Aloe micracantha*		X			
*Aloe minima*	X				
*Aloe modesta*				X	
*Aloe mudenensis*		X			
*Aloe myriacantha*	X				
*Aloe neilcrouchii*				X	
*Aloe nicholsii*					X
*Aloe parvibracteata*	X				
*Aloe parviflora*			X		
*Aloe pluridens*	X				
*Aloe pratensis*	X				
*Aloe prinslooi*				X	
*Aloe pruinosa*				X	
Aloe reitzii var. vernalis			X		
*Aloe rupestris*	X				
*Aloe saundersiae*				X	
*Aloe sharoniae*	X				
*Aloe spectabilis*	X				
*Aloe spicata*	X				
*Aloe suffulta*	X				
*Aloe suprafoliata*	X				
*Aloe thraskii*		X			
*Aloe umfoloziensis*		X			
*Aloe vanbalenii*	X				
*Aloe vanrooyenii*	X				
*Aloe viridiana*	X				
*Aloe vryheidensis*	X				
**Total = 49**	**30**	**7**	**3**	**8**	**1**

## Conservation and protection status

Aloes are protected under both provincial legislation and international convention. All KwaZulu-Natal aloes are listed as specially protected under KwaZulu-Natal nature conservation legislation (Province of KwaZulu-Natal 1997) and may, therefore, not be removed from the wild without the necessary permits. Furthermore, all species of *Aloe* [or, at least, species treated in this genus prior to the new generic classification published in Grace et al. (2013) and Manning et al. (2014); see Grace and Klopper (2014)] [except for *Aloe vera* (L.) Burm.f.] appear on CITES (Convention on the International Trade in Endangered Species of Wild Fauna and Flora) Appendices, meaning that international trade in aloes is controlled to prevent utilisation that would be incompatible with their survival. The taxa occurring in KwaZulu-Natal are all included in Appendix II (CITES 2018), necessitating CITES permit arrangements for such trade. In practice, most aloes are nonetheless subjected to ongoing illegal removal of plants from the wild, coupled to impacts resulting from anthropogenic degradation of their habitats.

Conservation status and threats to the survival of each species are given here according to Raimondo et al. (2009) and the Red List of South African Plants website (http://redlist.sanbi.org), reflecting the 2001 IUCN Red List categories (IUCN 2001). Where an assessment is not available in these sources, the information given was obtained from Lize von Staden (personal communication) of the Threatened Species Programme at the South African National Biodiversity Institute in Pretoria. Many of these conservation statuses are still under review and might change as further evidence becomes available. The majority of aloes in KwaZulu-Natal (30 taxa or 61%) falls in the Least Concern (LC) category. For *Aloe cooperi* Baker, the population trend is considered to be Declining. Nineteen (39%) of the KwaZulu-Natal aloes are regarded as taxa of conservation concern (NT, VU, EN, CR): seven taxa (14%) are Near-Threatened (NT). A total of twelve aloes (24%) are threatened (VU, EN, CR): three (6%) are Vulnerable (VU); eight (16%) are Endangered (EN); and one (2%) is Critically Endangered (CR) (see Table 2).

## Key to the aloes of KwaZulu-Natal

**Note.** To reliably identify aloes species, knowledge of the morphology of mature plants and their reproductive structures and, often, their geographical origin, are essential. Accordingly, this key does not cater for juvenile or sterile material and only applies to plants in the wild or collected/cultivated ones of known provenance.

**Table:** 

1	Tangled shrubs with very slender stems; leaves cauline dispersed	***Aloiampelos tenuior***
–	Plants do not form tangled shrubs, stems more robust when present; leaves rosulate to distichous	**2**
2	Arborescent plants with stems longer than 1 m, usually longer than 2 m	**3**
–	Acaulescent plants or plants with stems shorter than 1 m	**13**
3	Stems dichotomously branched, without persistent dried leaves	**4**
–	Stems simple or branched, with persistent dried leaves	**5**
4	Tree aloe of up to 18 m high; leaves 60–90 cm long; inflorescence 0.4–0.6 m high, 3-branched from a single point; raceme cylindrical, 20–30 cm long; flowers rose to rose-pink, 33–37 mm long; sparsely scattered in a broad coastal zone throughout KwaZulu-Natal, but absent from most of the Maputaland Centre	***Aloidendron barberae***
–	Tree aloe of up to 8 m high; leaves 40–59 cm long; inflorescence ± 0.35 m high, up to 6-branched; raceme capitate, 4–6 cm long; flowers yellowish-orange, 47–50 mm long; confined to the Maputaland Center of Endemism	***Aloidendron tongaense***
5	Inflorescence simple or occasionally up to 2-branched	**6**
–	Inflorescence always branched	**8**
6	Much-branched shrub; pedicels 35–40 mm long; flowers scarlet to yellow, ± 40 mm long, cylindrical	***Aloe arborescens***
–	Stem simple or sometimes few-branched; pedicels absent; flowers pinkish-brown to greenish-yellow, up to 20 mm long, campanulate	**7**
7	Stem erect to decumbent; leaves spreading to recurved; inflorescence erect; raceme 4–5 cm wide; ovary uniformly green	***Aloe spicata***
–	Stem procumbent to shortly suberect, sometimes absent; leaves arcuate-erect to slightly spreading; inflorescence oblique to erect; raceme 5–7 mm wide; ovary green with red line longitudinally down the three broad angles	***Aloe vryheidensis***
8	Racemes horizontal or spreading to suboblique; flowers secund	***Aloe marlothii***
–	Racemes erect; flowers not secund	**9**
9	Leaves obscurely lineate; floral bracts ± 20 mm long; pedicels 30–35 mm long; flowers 40–45 mm long	***Aloe pluridens***
–	Leaves without spots or lines; floral bracts shorter than 10 mm; pedicels shorter than 6 mm; flowers shorter than 35 mm	**10**
10	Racemes 50–80 cm long; pedicels ± 6 mm long	***Aloe candelabrum***
–	Racemes up to 25 cm long; pedicels up to 3 mm long	**11**
11	Leaves 30–70 cm long, without surface prickles or spines; racemes ± 7 cm wide; floral bracts ± 1 mm long; flowers 15–50 mm long	***Aloe rupestris***
–	Leaves ± 100 cm or longer, with spines in median line on lower surface or with copious surface spines; racemes wider than 9 mm; floral bracts longer than 4 mm; flowers ± 25 mm and longer	**12**
12	Leaves suberect to spreading, ± 100 × 12–15 cm, usually with copious spines on both surfaces; floral bracts 4–5 mm long; flowers ± 32 mm long	***Aloe spectabilis***
–	Leaves gracefully recurved, ± 160 × 22 cm, lower surface sometimes with few spines in median line; floral bracts ± 9 mm long; flowers ± 25 mm long	***Aloe thraskii***
13	Leaves thick and succulent, usually with fierce marginal teeth	**14**
–	Leaves thin and not very succulent, usually with small marginal teeth; grass aloes	**30**
14	Leaves with tuberculate spots or spines on lower surface, up to 17 cm long	**15**
–	Leaves without surface spines, if spines are present on median line of lower surface, then leaves longer than 40 cm	**16**
15	Leaves with several scattered small, white, subtuberculate to spinulescent spots on both surfaces, 8–10 × 1–2 cm, marginal teeth 1–2 mm long; peduncle mostly without sterile bracts	***Aristaloe aristata***
–	Leaves with few scattered brown spines on lower surface, especially along median line, 10–17 × 4–6 cm, marginal teeth ± 5 mm long; peduncle covered with large imbricate sterile bracts	***Aloe pratensis***
16	Leaves with numerous spots on one or both surfaces, often in confluent transverse bands; flowers usually with globose basal swelling	**17**
–	Leaves without spots, sometimes with a few scattered spots only; flowers without globose basal swelling	**26**
17	Inflorescence with very slender peduncle, twining or climbing, requiring support from surrounding vegetation	***Aloe suffulta***
–	Inflorescence with robust peduncle, not climbing, stands erect without support from surrounding vegetation	**18**
18	Racemes capitate, rather dense	**19**
–	Racemes cylindrical, rather lax	**21**
19	Flowers pale whitish-green, tinged with pink, 13–17 mm long, globose basal swelling not very prominent	***Aloe prinslooi***
–	Flowers salmon pink to orange or red, longer than 30 mm, with prominent globose basal swelling	**20**
20	Inflorescence 0.4–1.0 m high; racemes 10–12 cm long; floral bracts 12–23 mm long; pedicels 35–45 mm long	**Aloe maculata subsp. maculata**
–	Inflorescence 1.0–1.5 m high; racemes 7–9 cm long; floral bracts 8–12 mm long; pedicels 10–15 mm long	***Aloe umfoloziensis***
21	Rosettes suckering profusely to form large dense groups	**22**
–	Rosettes usually solitary or sometimes suckering to form small groups	**23**
22	Leaves with markings more pronounced on lower surface; flowers light to dark flesh pink, with bloom, 28–30 mm long	***Aloe viridiana***
–	Leaves usually without markings on lower surface; flowers dull to somewhat glossy red, without bloom, 30–40 mm long	***Aloe parvibracteata***
23	Inflorescence 1- or 2-branched; floral bracts 8–10 mm long; peduncle cannot support weight of very large mature capsules and bends towards ground	***Aloe vanrooyenii***
–	Inflorescence with more than four branches; floral bracts longer than 10 mm; peduncle remains erect in fruiting stage	**24**
24	Inflorescence 4- to 8-branched; pedicels 20–25 mm long; flowers bright, without a powdery bloom, 25–35 mm long	***Aloe mudenensis***
–	Inflorescence up to 12-branched; pedicels up to 20 mm long; flowers dull, with powdery bloom, up to ± 40 mm long	**25**
25	Rosettes always acaulescent, erect; leaves glossy, without spots on the lower surface, marginal teeth up to 10 mm long; inflorescence 2–3 m high; perianth 14 mm across ovary	***Aloe dewetii***
–	Rosettes usually with very short procumbent stem; leaves with heavy powdery bloom, with spots more numerous on lower surface, marginal teeth 3–4 mm long; inflorescence 1.4–2.0 m high; perianth 8 mm across ovary	***Aloe pruinosa***
26	Leaves not obscurely lineate; raceme 30–40 cm long; pedicels up to 5 mm long; flowers pointing downwards and pressed against stalk	**27**
–	Leaves obscurely lineate; raceme shorter than 30 cm; pedicels 14–25 mm long; flowers spreading to pendent, but not pressed against stalk	**28**
27	Leaves with marginal teeth 10–15 mm apart; inflorescence 1.0–1.3 m high, simple in young plants, 1- to 3-branched in mature plants; floral bracts ± 18 mm long; flowers yellowish-orange, 24–30 mm long	***Aloe gerstneri***
–	Leaves with marginal teeth ± 5 mm apart; inflorescence 0.70–0.75 m high, 2- to 4-branched; floral bracts ± 6 mm long; flowers bright red above, lemon yellow below, 32–40 mm long	**Aloe reitzii var. vernalis**
28	Rosettes usually solitary; inflorescence simple	***Aloe suprafoliata***
–	Rosettes suckering to form dense groups; inflorescence branched	**29**
29	Inflorescence 6- to 12-branched; racemes rather lax; floral bracts 3–6 mm long; flowers usually pale red, with stamens and style exserted to 2 mm	**Aloe chabaudii var. chabaudii**
–	Inflorescence 2- or 3-branched; racemes rather dense; floral bracts up to 15 mm long; flowers usually orange-yellow, with stamens and style exserted to 12 mm	***Aloe vanbalenii***
30	Plants with an underground bulb-like swelling of the leaf bases	**31**
–	Plants without an underground bulb-like swelling of the leaf bases	**34**
31	Racemes subcapitate; flowers scented	***Aloe modesta***
–	Racemes cylindrical; flowers unscented	**32**
32	Inflorescence ± 0.15 m high; raceme very dense; pedicels absent	***Aloe inconspicua***
–	Inflorescence longer than 0.2 m high; raceme lax; pedicels present (short or long)	**33**
33	Flowers sub-erect to horizontal, pale pink to coral pink with darker median stripes on perianth segments, with bilabiate mouth	***Aloe bergeriana***
–	Flowers pendent, pale pink to scarlet, green-tipped, with mouth not bilabiate	***Aloe kniphofioides***
34	Leaves strongly keeled, V-shaped in cross section	**35**
–	Leaves not strongly keeled and V-shaped in cross section	**37**
35	Inflorescence up to 0.3 m high; flowers 15–20 mm long, with bilabiate upturned mouth	***Aloe myriacantha***
–	Inflorescence longer than 0.3 m; flowers longer than 25 mm, with mouth not bilabiate or upturned	**36**
36	Leaves with the margin toothed throughout; inflorescence sometimes shorter than the leaves; floral bracts flat, not clasping the pedicels	***Aloe cooperi***
–	Leaves with no marginal teeth in the upper 2/3; inflorescence longer than the leaves; floral bracts clasping the pedicels	***Aloe sharoniae***
37	Leaves up to 3.5 cm wide	**38**
–	Leaves usually wider than 3.5 cm	**44**
38	Leaves wider than 2 cm	**39**
–	Leaves up to 1 cm wide	**40**
39	Rosettes usually solitary; flowers 25–40 mm long, mouth not upturned	***Aloe micracantha***
–	Rosettes usually in dense groups; flowers 13–16 mm long, mouth distinctly upturned	***Aloe nicholsii***
40	Flowers yellow to greenish-yellow	**41**
–	Flowers dull or greenish-white to pink or purple	**42**
41	Rosettes in dense groups; leaves rosulate; raceme ± 4 cm long; flowers 13–18 mm long, with stamens and style exserted 4–7 mm	***Aloe dominella***
–	Rosettes usually solitary, sometimes in small groups; leaves usually distichous; raceme ± 2 cm long; flowers ± 12 mm long, with stamens and style exserted to 2 mm	***Aloe linearifolia***
42	Leaves up to 10 cm long; inflorescence shorter than 0.2 m	***Aloe saundersiae***
–	Leaves longer than 20 cm; inflorescence taller than 0.25 m	**43**
43	Leaves rosulate, 25–35 × 0.4–0.6 cm; inflorescence 0.25–0.50 m high; peduncle smooth; flowers 10–11 mm long	***Aloe minima***
–	Leaves distichous or rosulate, 20–25 × 0.6–0.8 cm; inflorescence ± 0.4 m high; peduncle with numerous small spines on lower part; flowers ± 8 mm long	***Aloe parviflora***
44	Leaves usually distichous or sub-distichous, up to 6 cm wide	**45**
–	Leaves rosulate, usually wider than 6 cm	**46**
45	Flowers usually apricot-yellow, 28–30 mm long	***Aloe hlangapies***
–	Flowers yellow, 16–18 mm long	***Aloe kraussii***
46	Stem up to 0.95 m long, decumbent to erect, branched; inflorescence 0.6–0.8 m high; floral bracts ± 30 mm long; flowers ± 45 mm long	***Aloe neilcrouchii***
–	Stem absent or very short, erect, usually simple; inflorescence up to 0.6 m high; floral bracts shorter than 25 mm; flowers up to 40 mm long	**47**
47	Leaves erect, 50–60 cm long; floral bracts 20–23 mm long; pedicels 40–45 mm long; flowers 30–40 mm long	***Aloe boylei***
–	Leaves erectly spreading, 30–40 cm long; floral bracts 10–15 mm long; pedicels 30–40 mm long; flowers 20–24 mm long	***Aloe ecklonis***

## Species treatments

Species are arranged alphabetically according to species name, with the minor genera treated before the true aloes. Only common names that are relevant to KwaZulu-Natal are given. Further common names can be found in Grace et al. (2011).

^E^ indicates taxa that are endemic to KwaZulu-Natal.

^NE^ indicates taxa that are near-endemic to KwaZulu-Natal (more than 75% of the distribution range falls within this province).

### Aloiampelos tenuior

Taxon classificationPlantaeAsparagalesAsphodelaceae

(Haw.) Klopper & Gideon F.Sm.

E482C826-FB2E-59E3-8178-36D032C951E2

#### Syn.

*Aloe tenuior* Haw.

#### Common names.

Fence aloe, gardener’s aloe (English); heiningaalwyn, heuningaalwyn (Afrikaans).

#### Description.

Tangled shrub of 0.6 m or higher. ***Stems*** slender, 1–3 m long, branched low down or higher, erectly spreading or scandent to recurved or decumbent, without persistent dried leaves. ***Leaves*** cauline dispersed, erectly spreading, glaucous green, without spots, linear-lanceolate, 10–18 cm long, 1.0–2.2 cm wide; sheath obscurely green-lineate, not auriculate, 0.5–2.5 cm long; margin narrow, white, cartilaginous, with minute, white teeth, up to 0.5 mm long, 1–2 mm apart. ***Inflorescence*** 0.3–0.4 m high, ascending to erect, simple or 1- or 2-branched. ***Racemes*** cylindrical, slightly acuminate, 10–20 cm long, rather dense to dense. ***Floral bracts*** ± 5 mm long, 1–2 mm wide. ***Pedicels*** 3–5 mm long. ***Flowers***: *perianth* yellow, orange or red with yellow tips, 11–15 mm long, ± 2 mm across ovary, very slightly narrowed above ovary, widening towards mouth, cylindrical; outer segments free for 3–6 mm; *stamens* and *style* exserted 4–6 mm.

#### Flowering time.

(August) October–December (May).

#### Habitat.

Often in open habitats on sandy soils, more rarely in thicket vegetation, sometimes on steep slopes. In contrast, other species of *Aloiampelos* that do not occur in KwaZulu-Natal, such as *A. ciliaris* (Haw.) Klopper & Gideon F.Sm. from the Eastern Cape, more commonly occur in thicket or fynbos.

#### Diagnostic characters.

*Aloiampelos tenuior* is the only aloe indigenous to KwaZulu-Natal that forms an untidy tangled shrub with thin slender stems. Also diagnostic is its cauline dispersed, blue-green leaves, with distinct sheaths that are obscurely lined. Racemes are elongated, with small red, orange or yellow cylindrical, uncurved flowers and long-exserted stamens and style.

#### Conservation status.

Least Concern (Raimondo et al. 2009).

#### Distribution.

**Figure 2.**
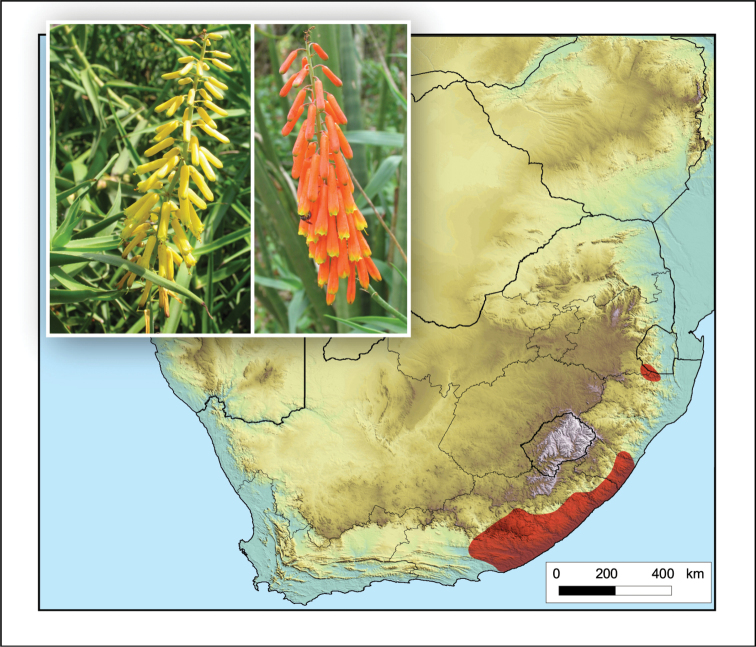
*Aloiampelos tenuior*. Photos: N.R. Crouch.

Occurs from the Port Elizabeth and Jansenville areas in the Eastern Cape into southern KwaZulu-Natal, the Richmond area and then with a disjunct distribution in northern KwaZulu-Natal on the border with Mpumalanga (South Africa) and Eswatini (Fig. 2).

### Aloidendron barberae

Taxon classificationPlantaeAsparagalesAsphodelaceae

(Dyer) Klopper & Gideon F.Sm.

4904DE90-0D29-541B-84DC-353662B4BF57

#### Syn.

*Aloe barberae* Dyer.

#### Common names.

Tree aloe (English); boomaalwyn, mikaalwyn (Afrikaans); impondondo, indlabendlazi, inkalane unkulu, umgxwala (Zulu).

#### Description.

Arborescent plant, up to 18 m high. ***Stem*** 10–18 m high, profusely branched dichotomously and rebranched from about middle, erect, without persistent dried leaves. ***Leaves*** densely rosulate, recurved, dull green, without spots, ensiform, deeply channelled, 60–90 cm long, 7–9 cm wide at base; sheath with greenish-white marginal border; margin narrow, white, cartilaginous, with firm, horny, brownish tipped, dull white, deltoid teeth, 2–3 mm long, 10–25 mm apart. ***Inflorescences*** 0.4–0.6 m high, erect, dichotomously 3-branched. ***Racemes*** cylindrical, slightly acuminate, 20–30 cm long, dense. ***Floral bracts*** 8–10 mm long, ± 1 mm wide. ***Pedicels*** 7–10 mm long. ***Flowers***: *perianth* rose to rose-pink, greenish tipped, 33–37 mm long, ± 9 mm across ovary, not narrowed above ovary, widening towards middle, narrowing somewhat towards upturned mouth, cylindrical-ventricose; outer segments free almost to base; *stamens* exserted to 15 mm; *style* exserted 15–20 mm.

#### Flowering time.

May–August.

#### Habitat.

Dense, tall bush and low forest, rocky slopes of wooded valleys.

#### Diagnostic characters.

*Aloidendron barberae* is one of only two large-growing tree aloes indigenous to KwaZulu-Natal. These two aloes both have dichotomously branched stems and branches that lack persistent dried leaves. *Aloidendron barberae* differs from *Aloidendron tongaense* in being much taller (up to 18 m) with more branches and having larger bright green leaves of 60–90 cm long (not dull green and 40–59 cm); their distribution ranges are also mutually exclusive. The inflorescence is also slightly taller at 0.4–0.6 m (not ± 0.35 m) and 3-branched from a single point (not up to 6-branched), with longer cylindrical racemes of 20–30 cm long (not capitate and 4–6 cm), bearing straight rose-pink flowers that are 33–37 mm long (not curved yellow flowers of 47–50 mm) with stamens exserted to 15 mm at anthesis (not 3–5 mm).

#### Conservation status.

Least Concern (Raimondo et al. 2009).

#### Distribution.

**Figure 3.**
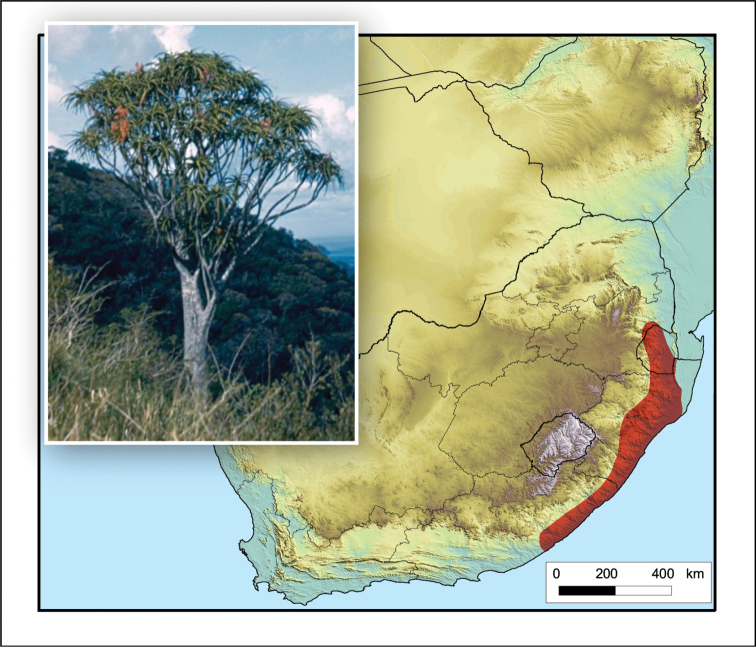
*Aloidendron barberae*. Photo: G.W. Reynolds.

Occurs in scattered localities, often in inaccessible sites (with steep gradients), in a broad coastal zone from East London in the Eastern Cape, through KwaZulu-Natal and Mpumalanga, South Africa, also in Eswatini (Fig. 3).

#### Notes.

*Aloidendron barberae* is often cited as occurring in Mozambique, the latest of these being Van Jaarsveld and Judd (2015). However, an examination of available herbarium specimens at several South African and European herbaria has shown that specimens from Mozambique all represent *A. tongaense* (Walker et al. 2019b). This is supported by Burrows et al. (2018) who only treat the latter species. However, considering that *A. barberae* is common on the South African side of the Lebombo range, it may well be present in nearby southern Mozambique, which borders on the foothills of the range. Further investigation is needed to confirm whether or not *A. barberae* is present in this botanically under-explored part of Mozambique.

### Aloidendron tongaense

Taxon classificationPlantaeAsparagalesAsphodelaceae

(Van Jaarsv.) Klopper & Gideon F.Sm.

67F1F5DC-EF17-58C6-BC6D-D6C8563F62CC

#### Syn.

*Aloe tongaensis* Van Jaarsv.

#### Description.

Tree, 4–8 m high, with rounded crown. ***Trunk*** 60–80 cm diameter at base, erect, dichotomously branched, without persistent dried leaves, with grey bark. ***Leaves*** rosulate at branch tips, spreading to recurved, dull green, without markings, leathery, ensiform, upper surface canaliculate, 40–59 cm long, 4.5 cm wide; margin with teeth, 2 mm long, 5–10 mm apart. ***Inflorescence*** ± 0.35 m tall, erect, up to 6-branched. ***Racemes*** capitate, 4–6 cm long, rather dense. ***Floral bracts*** 12–14 mm long, 3–4 mm wide. ***Pedicels*** 10–14 mm long. ***Flowers***: *perianth* yellowish-orange, 47–50 mm long, 8–9 mm across ovary, narrowing very slightly towards mouth, cylindrical, curved; outer segments free for 10 mm; *stamens* exserted 3–5 mm; *style* exserted to 7 mm.

#### Flowering time.

Mainly April–May.

#### Habitat.

Sand forest and coastal dune forest, in warm, humid, tropical/subtropical conditions, on sandy soil.

#### Diagnostic characters.

*Aloidendron tongaense* is one of only two large tree aloes indigenous to KwaZulu-Natal. These two aloes both have dichotomously branched stems that lack persistent dried leaves. *Aloidendron tongaense* differs from *Aloidendron barberae* in being a shorter tree (up to 8 m, not up to 18 m) with fewer branches and having smaller dull green leaves of 40–59 cm long (not bright green and 60–90 cm). The inflorescence is also slightly shorter at ± 0.35 m (not 0.4–0.6 m) and up to 6-branched (not 3-branched from a single point), with shorter capitate racemes of 4–6 cm long (not cylindrical and 20–30 cm), bearing curved yellowish-orange flowers that are 47–50 mm long (not straight rose-pink flowers of 33–37 mm) with stamens exserted 3–5 mm at anthesis (not up to 15 mm).

#### Conservation status.

Least Concern (Von Staden et al. 2013).

#### Distribution.

**Figure 4.**
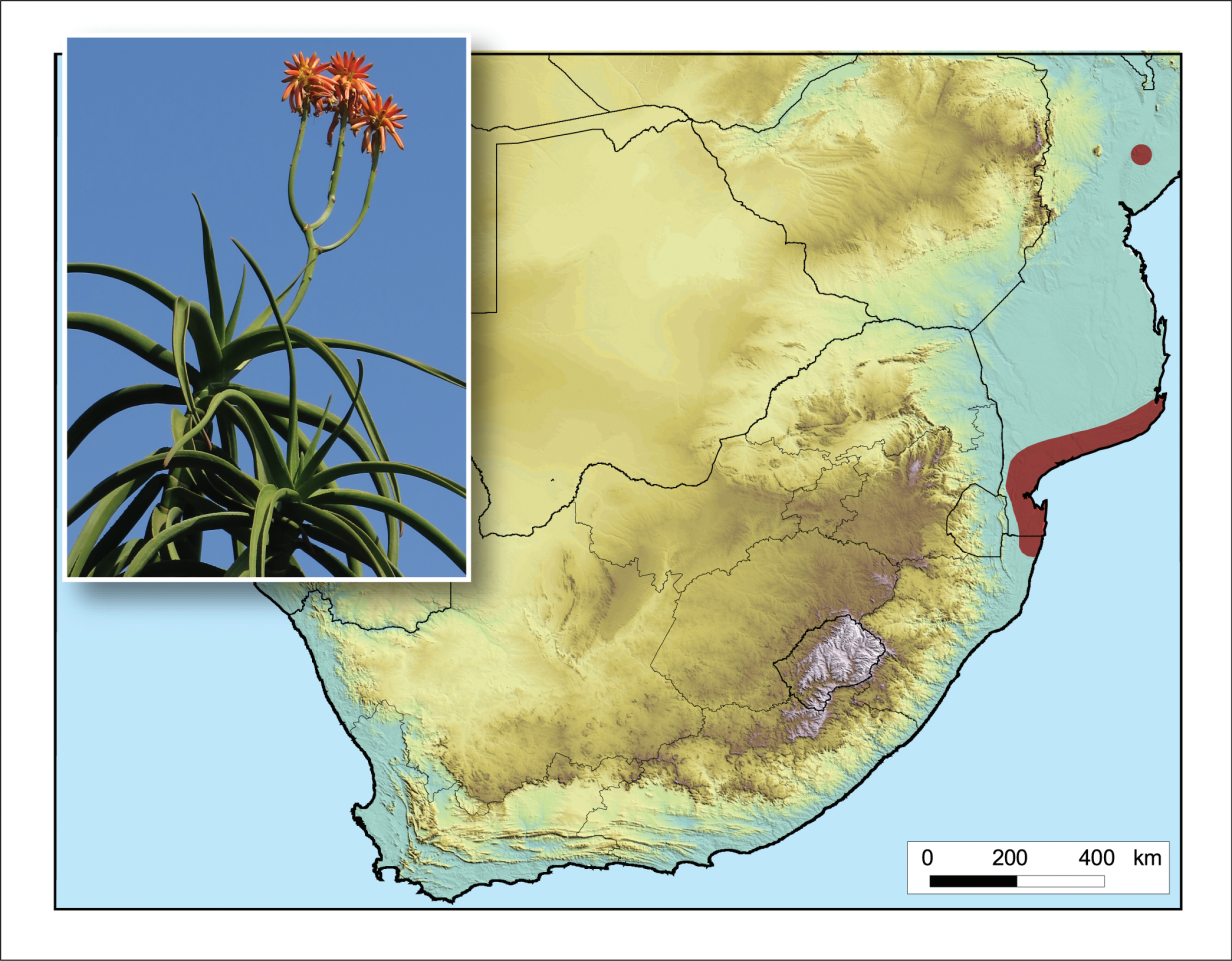
*Aloidendron tongaense*. Photo: N.R. Crouch.

Occurs in the sand forest and coastal dune forest at Kosi Bay in northern KwaZulu-Natal (Maputaland), South Africa and along the southern Mozambique coast as far north as Inhambane, with a known disjunct collection further north in the Cheringoma District of east-central Mozambique (Fig. 4). It is a near-endemic of the Maputaland Centre of Endemism (Van Wyk and Smith 2001).

#### Notes.

This aloe was previously considered to be a coastal form of *A. barberae*, but was later accorded species status (Van Jaarsveld 2010). Although the protologue and subsequent literature states that the species is only known from northern KwaZulu-Natal, South Africa and adjacent areas of southern Mozambique, herbarium specimens indicate that it occurs much wider in Mozambique. In fact, numerous specimens from lowland parts of Mozambique, previously regarded as *A. barberae*, have now been assigned to *A. tongaense* (Walker et al. 2019b).

### Aristaloe aristata

Taxon classificationPlantaeAsparagalesAsphodelaceae

(Haw.) Boatwr. & J.C.Manning

8A268E3E-4D04-598F-8E44-1DE29D1B2E98

#### Syn.

*Aloe aristata* Haw.

#### Common names.

Guinea-fowl aloe (English); tarentaalaalwyn (Afrikaans); umathithibala (Zulu).

#### Description.

Acaulescent plants; rosettes solitary or usually suckering to form dense clumps. ***Leaves*** densely rosulate, erect to arcuate-incurved, green to grey-green, with several scattered small, white spots, more copiously spotted with spots in more or less transverse bands on lower surface, spots subtuberculate to spinulescent, soft white spines in 1 or 2 rows at apex of keel, narrowly lanceolate to deltoid, tapering to hair-like awn, leaf 8–10 cm long, 1–2 cm wide at base; margin with soft, white, cartilaginous teeth, 1–2 mm long, 1–2 mm apart at mid-leaf. ***Inflorescence*** 0.2–0.5 m high, erect, usually 2- to 6-branched, occasionally simple. **Racemes** subcapitate, 10–20 cm long, rather lax. ***Floral bracts*** 11–12 mm long, 4 mm wide. ***Pedicels*** 20–35 mm long. ***Flowers***: *perianth* red on upper surface, paler below, ± 40 mm long, ± 7 mm across ovary, slightly narrowed above ovary, slightly widening towards middle, narrowing at mouth, base somewhat globose, tube slightly decurved; outer segments free for 7 mm; *stamens* exserted to 1 mm; *style* exserted 1–2 mm.

#### Flowering time.

August–October (November).

#### Habitat.

Wide variety of habitats, including sandy to clayey soils in hot, dry karroid areas, deep shade on humus-rich soil in riverine forest and montane forest and grassland on high mountains in Lesotho.

#### Diagnostic characters.

*Aristaloe aristata* can easily be distinguished from other KwaZulu-Natal aloes by being an acaulescent plant with small haworthia-like rosettes (10–15 cm diameter) that sometimes occur solitary, but more often sucker to form dense groups. The leaves (8–10 × 1–2 cm) have numerous, tuberculed, white-spots with long, thin, hair-like tips on both surfaces. The inflorescence (0.2–0.5 m high) is usually 2- to 6-branched or occasionally simple with the peduncle without sterile bracts. Racemes are subcapitate and rather lax. Flowers are tubular and slightly curved (± 40 mm long), with a basal swelling around the ovary. The uppermost (dorsal) portion of the pedicel and flower, which receive more sun, are deeper red than the paler lower (ventral) portion. This is the only South African *Aloe* species that resembles a member of *Haworthia* Duval when not in flower.

#### Conservation status.

Least Concern (Raimondo et al. 2009).

#### Distribution.

**Figure 5.**
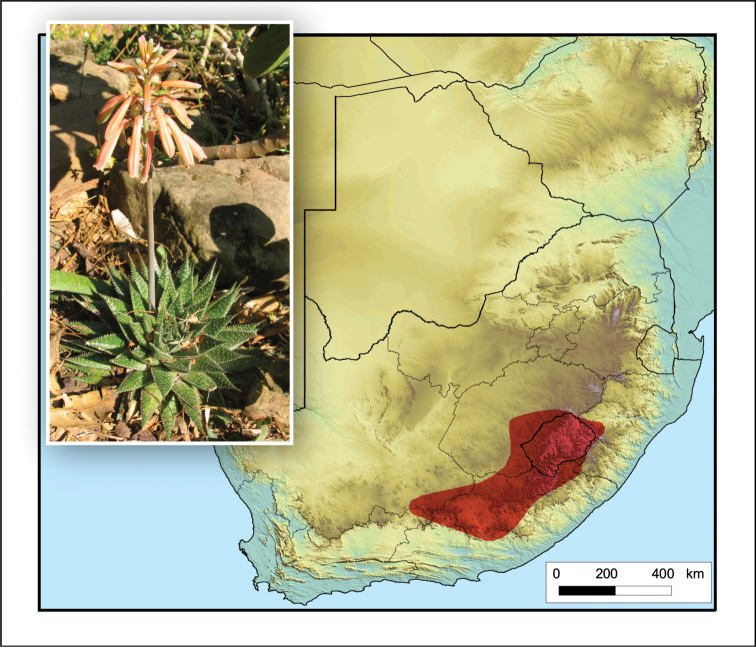
*Aristaloe aristata*. Photo: N.R. Crouch.

Widespread from Beaufort West (Western Cape) in the central Great Karoo, through the Eastern Cape and eastern Free State to south-western KwaZulu-Natal, South Africa, as well as in Lesotho (Fig. 5).

### Aloe arborescens

Taxon classificationPlantaeAsparagalesAsphodelaceae

Mill. (including A. arborescens subsp. mzimnyati Van Jaarsv. & A.E.van Wyk)

47DC3388-4C3C-5375-947C-E5DF4937553D

#### Common names.

Krantz aloe (English); kransaalwyn (Afrikaans); inhlaba-encane, inhlazi, inkalane, inkalane-encane, umhlabana (Zulu).

#### Description.

Much-branched shrub, 2–5 m high. ***Stems*** erect, with persistent dried leaves. ***Leaves*** densely rosulate at branch apices, spreading-recurved, dull green to grey-green, tinged reddish in dry conditions, without spots, texture smooth, lanceolate-attenuate, 40–60 cm long, 5–7 cm wide at base; margin with firm, pale teeth, 3–5 mm long, 5–20 mm apart at mid-leaf; exudate pale yellow. ***Inflorescences*** 0.6–0.8 m high, erect, usually simple, occasionally with 1 or 2 short branches. ***Racemes*** conical to conical-cylindrical, 20–30 cm long, dense. ***Floral bracts*** 15–20 mm long, 10–12 mm wide. ***Pedicels*** 35–40 mm long. ***Flowers***: *perianth* scarlet, often pink turning yellow at anthesis or occasionally yellow, ± 40 mm long, 7 mm across ovary, narrowed above ovary, widening to middle, narrowing slightly towards mouth, cylindrical-trigonous; outer segments free to base; *stamens* and *style* exserted to 5 mm.

#### Flowering time.

(February) June–July (August).

#### Habitat.

Usually in pockets of rich soil on krantz edges, rocky slopes and outcrops in areas of high summer rainfall, sometimes in dense bush.

#### Diagnostic characters.

*Aloe arborescens* is a much-branched shrub up to 5 m high, with stems rather robust (not thin and slender as in *Aloiampelos tenuior*) and leaves in dense rosettes at the branch apices. Leaves are greyish-green with pale yellow teeth. Inflorescences are usually simple with elongated conical racemes that are densely flowered. Floral bracts are large (15–20 mm long) with the pedicels twice as long (35–40 mm).

#### Conservation status.

Least Concern (Raimondo et al. 2009).

#### Distribution.

**Figure 6.**
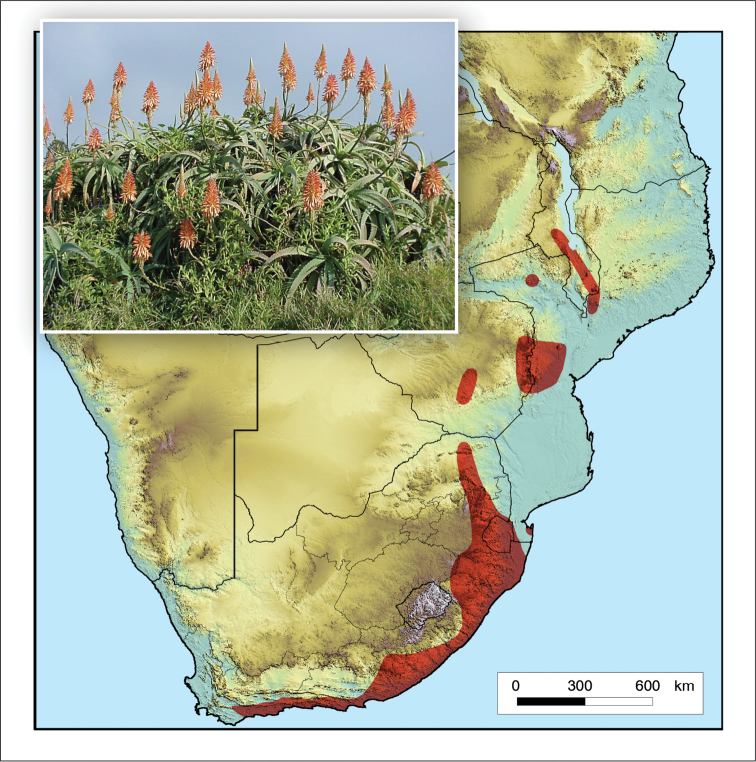
*Aloe arborescens*. Photo: G. Nichols.

The krantz aloe is very widely distributed in south-eastern Africa and has the third widest distribution range of all *Aloe* species. It occurs from the Cape Peninsula (where it has arguably become naturalised), along the south and east coast of South Africa, through the Western Cape, Eastern Cape and KwaZulu-Natal and inland to Mpumalanga and Limpopo, just entering the eastern Free State, as well as further north to Mozambique and the eastern mountains of Zimbabwe and Malawi (Fig. 6). A robust form of the species has become naturalised along the European Mediterranean coast (see, for example, Smith and Figueiredo 2009).

#### Notes.

In the past, several variations of *A. arborescens* have been afforded formal status at subspecific or varietal ranks, the most recent being A. arborescens subsp. mzimnyati Van Jaarsv. & A.E.van Wyk, which is endemic to the lower Mzimnyati River (Buffalo River) in KwaZulu-Natal. This subspecies is distinguished by its smaller growth habit (forming a shrub of 0.50–0.75 m high), its smaller, slightly clavate flowers (22–25 mm long) that vary in colour (orange-red to orange to yellow) within the same population and its slightly later flowering time (July–August) (Van Jaarsveld and Van Wyk 2005). We here follow the view of Smith et al. (2012), who concluded that it is better to regard *A. arborescens* as a single variable species, pending further research and, therefore, include A. arborescens subsp. mzimnyati in the synonymy of the species.

### Aloe bergeriana

Taxon classificationPlantaeAsparagalesAsphodelaceae

(Dinter) Boatwr. & J.C.Manning

578565BC-A7EC-559E-A448-59F2A9B3F6A7

#### Syn.

*Chortolirion bergeriana* Dinter.

#### Common names.

Kleinaalwyn (Afrikaans).

#### Description.

Herbaceous acaulescent perennial. ***Bulb*** usually solitary, ovoid-oblong, formed by pale rosy membraneous leaves-bases. ***Leaves*** rosulate, slightly succulent, grass-like, flaccid to erect, greyish-green, once twisted, 15–29 cm long, 1–3(– 5) mm wide in middle of leaf, leaf base hairy and unspotted; margins soft, white, decurved teeth. ***Inflorescence*** 20–35 cm high, simple, lower sterile parts bracteate, produced when leaves are fully developed. ***Racemes*** narrowly cylindrical, lax. ***Floral bracts*** 7 mm long, 4 mm wide. ***Pedicels*** 3–4 mm long. ***Flowers***: *perianth* pinkish-white with darker keel, 14–17 mm long, very slightly narrowed above ovary, cylindrical and straight to wide open bilabiate mouth, base obtuse; outer segments free almost to base; *stamens* and *style* hardly or not exserted.

#### Flowering time.

January–March.

#### Habitat.

Rocky sandstone and quartzitic outcrops.

#### Diagnostic characters.

*Aloe bergeriana* can be distinguished from other grass aloes in KwaZulu-Natal where the leaf bases form a subterranean bulb-like swelling (*Aloe inconspicua*, *Aloe kniphofioides* and *Aloe modesta*) by the very narrow leaves (15–29 × 0.1–0.3 cm) that are twisted once and hairy near the unspotted base, with rosy leaf bases. It is also characterised by the lax, unbranched, cylindrical raceme with shortly pedicellate, sub-erect to horizontal, pinkish-white, darker keeled, bilabiate, unscented flowers (14–17 mm long).

#### Conservation status.

Least Concern (Von Staden 2014a).

#### Distribution.

**Figure 7.**
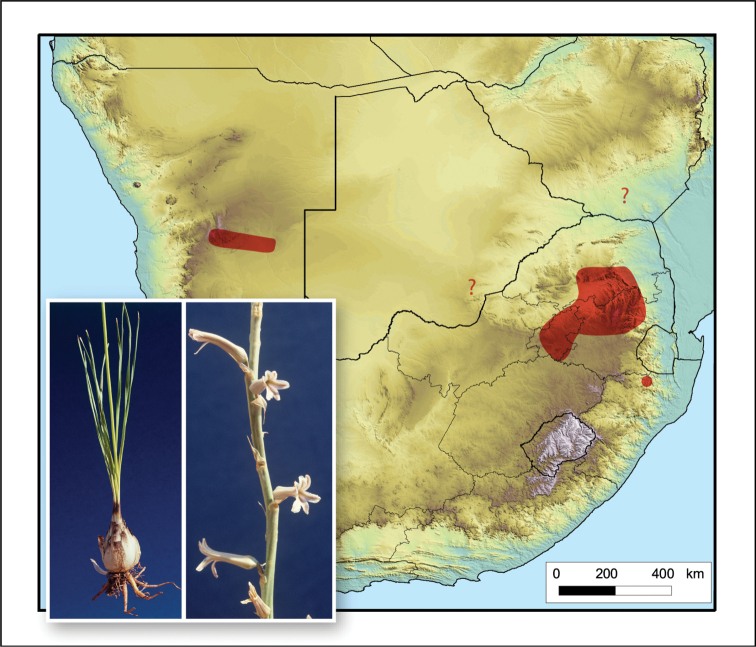
*Aloe bergeriana*. Photos: G.F. Smith.

Widespread but rare throughout Gauteng, Mpumalanga and Limpopo, South Africa, with records from KwaZulu-Natal in South Africa and Namibia, possibly also in Botswana and Zimbabwe (Fig. 7).

### Aloe boylei

Taxon classificationPlantaeAsparagalesAsphodelaceae

Baker

C5E1D8D6-CAF1-598E-9286-7EE93C4E2489

#### Common names.

Broad-leaved grass aloe (English); breëblaargrasaalwyn (Afrikaans); incothobe, isiphukuthwane, isiphuthumane, isiputhujane (Zulu).

#### Description.

Grass aloe. ***Stem*** short, up to 0.2 m long, simple or with offshoots from ground level to form dense groups, erect, dried leaves not persistent. ***Leaves*** rosulate, deciduous, erect, deep green, upper surface channelled, usually without spots, sometimes lineate or with few scattered spots near base, lower surface copiously white-spotted near base, lanceolate-ensiform, 50–60 cm long, 6–9 cm wide at base; margin with soft, white teeth, 1–3 mm long, 2–5 mm apart near base; exudate clear. ***Inflorescence*** 0.4–0.6 m high, erect, simple. ***Raceme*** capitate, sub-corymbose or slightly conical, 10–12 cm long, dense. ***Floral bracts*** 20–23 mm long, 5–7 mm wide. ***Pedicels*** 40–45 mm long. ***Flowers***: *perianth* salmon-pink, greenish tipped, 30–40 mm long, 11–12 mm across ovary, narrowing towards mouth, cylindrical, basally stipitate and narrowing into pedicel; outer segments almost free to base; *stamens* scarcely exserted or to 1–2 mm; *style* exserted 2–3 mm.

#### Flowering time.

December–January.

#### Habitat.

Eastern escarpment grassland, open rocky grassy hillsides.

#### Diagnostic characters.

*Aloe boylei* can be distinguished from other grass aloes in KwaZulu-Natal with its unkeeled leaves that are wider than 3.5 cm (*Aloe ecklonis*, *Aloe hlangapies*, *Aloe kraussii* and *Aloe neilcrouchii*), by the large rosette of erect, rosulate leaves (50–60 × 6–9 cm), with the upper surface usually without spots and the lower surface copiously white-spotted near the base. It is further characterised by the unbranched inflorescences (0.4–0.6 m high) that have dense, capitate, subcorymbose or slightly conical racemes (10–12 cm long) with large (30–40 mm long), salmon-pink, tubular flowers.

#### Conservation status.

Least Concern (L. von Staden pers. comm.).

#### Distribution.

**Figure 8.**
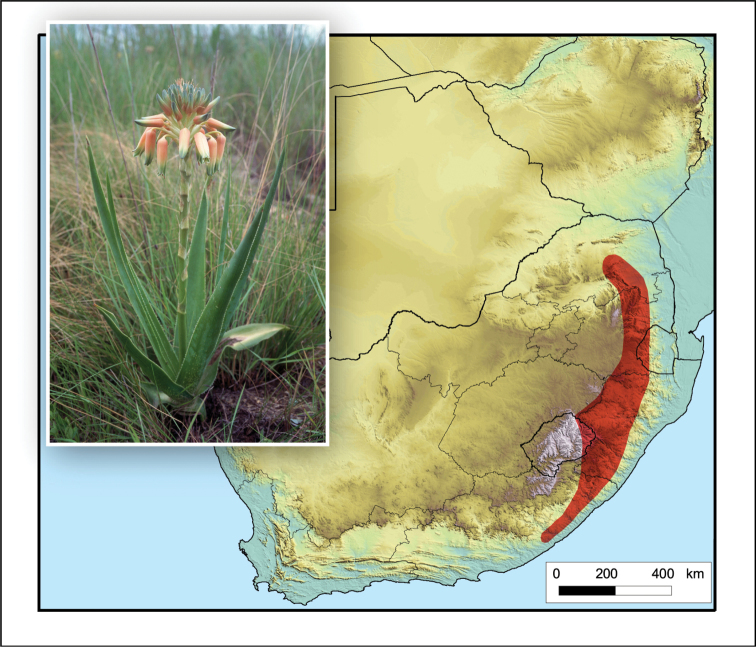
*Aloe boylei*. Photo: N.R. Crouch.

This species is widely distributed in eastern southern Africa, occurring in the Eastern Cape, western KwaZulu-Natal, eastern Free State, Mpumalanga and Limpopo in South Africa, as well as eastern Lesotho and western Eswatini (Fig. 8).

#### Notes.

*Aloe boylei* is considered by some as conspecific with *Aloe ecklonis* Salm-Dyck, together with *Aloe kraussii* Baker and *Aloe hlangapies* Groenewald (Glen and Hardy 2000; Carter et al. 2011).

### ^E^ Aloe candelabrum

Taxon classificationPlantaeAsparagalesAsphodelaceae

A.Berger

E1DCE10C-6A24-5CE3-A6BF-26AE0B46A63D

#### Common names.

Candelabrum aloe (English); doringaalwyn, kandelaaraalwyn (Afrikaans); umhlaba (Zulu).

#### Description.

Solitary, arborescent plant up to 2–4 m high. ***Stem*** simple, erect, 2–4 m high, densely covered with persistent dried leaves. ***Leaves*** densely rosulate, spreading to recurved, dull green to glaucous, without spots, surfaces smooth, lanceolate-ensiform, ± 100 cm long, 15 cm wide at base, under surface with few spines in median line near apex, occasionally with few scattered spines; margin reddish, cartilaginous, with pungent, reddish to reddish-brown, deltoid teeth, ± 3 mm long, 15–20 mm apart; exudate honey-coloured. ***Inflorescence*** usually single, ± 1 m high, erect, 6- to 12-branched. ***Racemes*** cylindrical, slightly acuminate, 50–80 cm long, terminal raceme, the longest and standing out higher than lateral racemes, very dense. ***Floral bracts*** ± 10 mm long, ± 5 mm wide. ***Pedicels*** 6 mm long. ***Flowers***: *perianth* scarlet, sometimes rose-pink or orange, rarely white, ± 32 mm long, ± 5 mm across ovary, widening above ovary towards slightly upturned mouth, clavate-cylindrical, slightly ventricose; outer segments free for 16–22 mm; *stamens* and *style* exserted 20 mm.

#### Flowering time.

June–July.

#### Habitat.

Thornveld and bushy places on rocky slopes and hills and undulating country.

#### Diagnostic characters.

*Aloe candelabrum* differs from the other tall often single-stemmed aloes in KwaZulu-Natal (*Aloe marlothii*, *Aloe pluridens*, *Aloe rupestris*, *Aloe spectabilis* and *Aloe thraskii*) with branched inflorescences, by having long (± 100 × 15 cm), spreading to recurved, deeply channelled leaves that sometimes have a few scattered spines on the lower surface and pungent, reddish to reddish-brown marginal teeth. The candelabra-like inflorescence is 6- to 12-branched with erect, very dense, cylindrical, slightly acuminate racemes of 50–80 cm long (the terminal raceme being longer than the lateral ones). Flowers are scarlet, sometimes rose-pink to orange, rarely white and ± 32 mm long with white inner segment tips.

#### Conservation status.

Near-threatened. Threats include habitat loss and degradation owing to silviculture, agriculture (mainly sugarcane) and urban expansion, as well as encroachment by alien invasives and illegal harvesting (L. von Staden pers. comm.).

#### Distribution.

**Figure 9.**
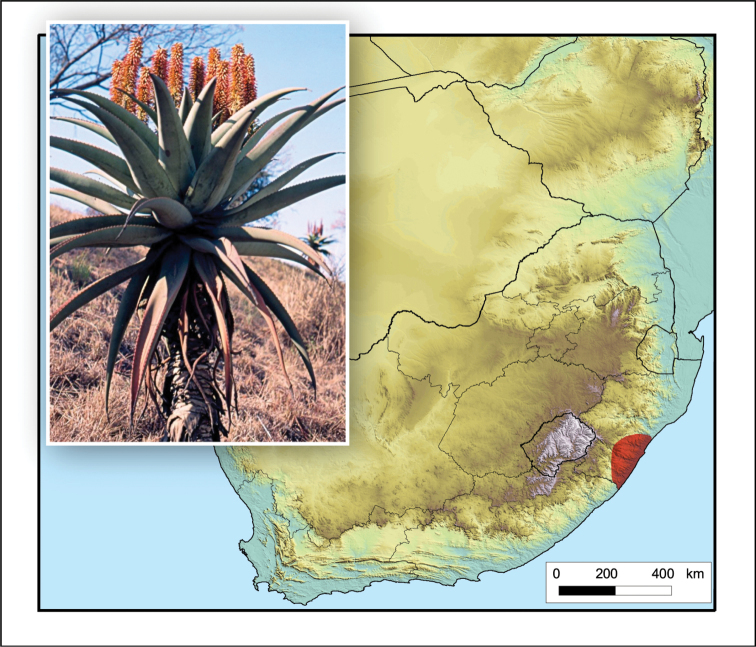
*Aloe candelabrum*. Photo: G.F. Smith.

More or less restricted to the valleys between the Umkhomazi and Umgeni Rivers in KwaZulu-Natal, South Africa (Fig. 9).

#### Notes.

The *Index kewensis* entry (now included in International Plant Names Index, www.ipni.org) for *Aloe candelabrum* Tod. in *Hortus Botanicus Panormitanus*: 46 (1876) is wrong as no such name exists. That reference is to *Agave candelabrum* Tod. in *Hortus Botanicus Panormitanus*: 66 (1876). This agave species is probably a synonym of *Agave cantala* (Haw.) Roxb. ex Salm-Dyck (Figueiredo and Smith 2012). The name *Aloe candelabrum* A.Berger is thus legitimate and not a later homonym (Reynolds 1950) as is often reported (e.g. Govaerts 2014).

Recognition of *Aloe candelabrum* as distinct from *Aloe ferox* Mill. (Smith et al. 2016), in the synonymy of which it is sometimes included, implies that *Aloe ferox*, a predominantly Western and Eastern Cape species that just enters the south-western Free State and southern Lesotho, does not occur in KwaZulu-Natal.

### Aloe chabaudii Schönland var. chabaudii

Taxon classificationPlantaeAsparagalesAsphodelaceae

031DABDC-9690-50CC-8860-FF89EC48EFD3

#### Common names.

Chabaud’s aloe (English); grysaalwyn (Afrikaans); inhlaba, inkalane (Zulu).

#### Description.

Acaulescent plants or stem very short, procumbent; rosettes up to 0.5 m high, suckering or dividing to form dense groups. ***Leaves*** densely rosulate, erect or spreading, dull grey-green to glaucous green, sometimes with reddish tinge, obscurely lineate, usually without spots, sometimes with few small confluent, H-shaped, scattered spots, ovate-lanceolate, acuminate, 30–60 cm long, 6–15 cm wide at base; margin cartilaginous, narrow, greyish, with small, deltoid, pale to brownish teeth, 1–3 mm long, 5–10 mm apart; exudate clear pale yellow. ***Inflorescence*** 0.5–1.5 m high, erect or oblique, 6- to 12-branched, lower branches rebranching. ***Racemes*** broadly cylindrical, slightly acuminate, occasionally sub-capitate, 5–15 cm long, rather lax. ***Floral bracts*** 3–6 mm long, 1.5–4.0 mm wide. ***Pedicels*** up to 20–25 mm long, spreading. ***Flowers***: *perianth* pale brick-red or bright coral-pink, sometimes orange to yellow, paler at mouth, 35–40 mm long, 7–9 mm across ovary, narrowed above ovary, widening towards mouth, cylindrical-trigonous, decurved; outer segments free for ± 8 mm; *stamens* exserted 1–2 mm; *style* exserted to 2 mm.

#### Flowering time.

April–August.

#### Habitat.

Usually on bare rock on granite domes, at foot of granite whalebacks and outcrops or in shallow soil pockets and shady wooded slopes. Frost-sensitive.

#### Diagnostic characters.

Aloe chabaudii var. chabaudii can be distinguished from other virtually acaulescent, non-maculate aloes in KwaZulu-Natal (*Aristaloe aristata*, *Aloe gerstneri*, *Aloe pratensis*, Aloe reitzii var. vernalis, *Aloe suprafoliata* and *Aloe vanbalenii*) by its suckering habit that results in the establishment of dense groups of rosettes. It is further characterised by its erect to spreading, greyish-green to glaucous green leaves (30–60 × 6–15 cm) with rather small closely-spaced marginal teeth. The inflorescence is erect to oblique, up to 1.5 m high and 6- to 12-branched with the lower branches spreading and rebranching. Floral bracts are short (3–6 mm) and pedicels oblique to almost horizontal (up to 25 mm). Flowers are mostly reddish, 35–40 mm long and narrowed above the ovary.

#### Conservation status.

Least Concern (Raimondo et al. 2009).

#### Distribution.

**Figure 10.**
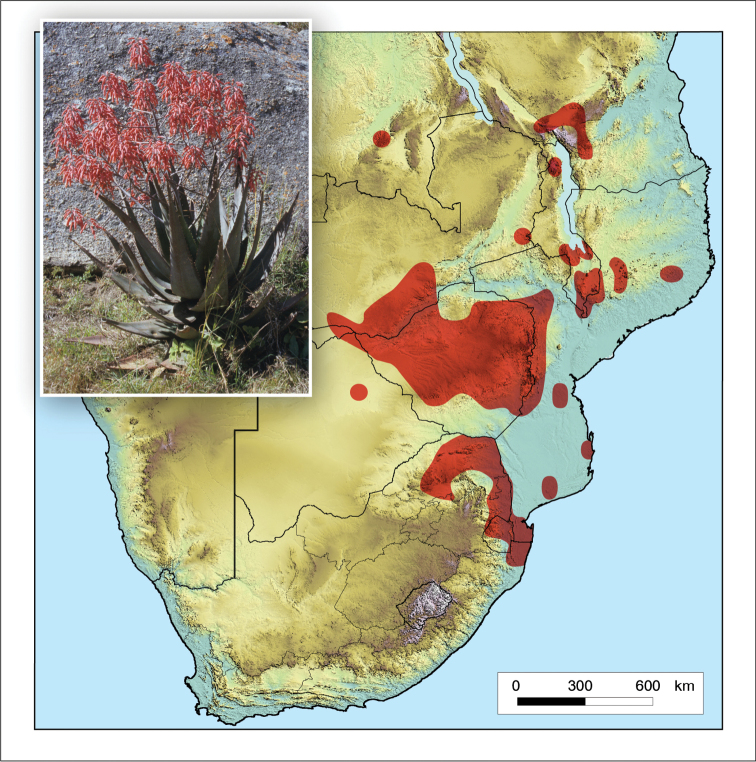
Aloe chabaudii var. chabaudii. Photo: M. Kimberley.

Centre of distribution in Zimbabwe, extending north to Zambia and Malawi and south-western Tanzania, west into eastern Botswana, the Caprivi Strip of north-eastern Namibia, east to Mozambique and south to the Limpopo, Mpumalanga and northern KwaZulu-Natal provinces of South Africa, as well as Eswatini (Fig. 10).

#### Notes.

One other variety is recognised in *A. chabaudii*, namely A. chabaudii var. mlanjeana Christian that is confined to the Mulanje Massif and hills in the Thyolo and Mulanje District, Malawi.

### Aloe cooperi

Taxon classificationPlantaeAsparagalesAsphodelaceae

Baker

DC4DA69D-97ED-505D-B196-1B7162F0E16C

#### Common names.

Cooper’s aloe (English); cooperse-aalwyn (Afrikaans); isipukutwane, isiputumane, inqimindolo (Zulu).

#### Description.

Grass aloe. Acaulescent plants or ***stem*** short, up to 0.15 m, erect, usually simple; rosettes solitary or sometimes with offshoots at ground level to form small groups; dried leaves not persistent. ***Leaves*** distichous, sometimes spirally twisted to rosulate in old plants, erect, deciduous, green, usually without spots on upper surface, with copious white spots at base on lower surface, obscurely lineate, narrowly long-deltoid, distinctly keeled, V-shaped in cross section, 40–80 cm long, 2.5–6.0 cm wide at base; margin with firm, white teeth, 1–2 mm long, 1–2 mm apart at mid-leaf; exudate clear. ***Inflorescences*** 0.4–1.0 m high, erect, simple. ***Raceme*** broadly conical, 10–20 cm long, dense. ***Floral bracts*** 20–35 mm long, 10 mm wide. ***Pedicels*** 30–60 mm long. ***Flowers***: *perianth* salmon-pink near base, green tipped, 25–40 mm long, ± 12 mm across ovary, narrowing towards mouth, roundly trigonous, basally stipitate and narrowing into pedicel; outer segments free almost to base; *stamens* not exserted or exserted 1–2 mm; *style* exserted to 5 mm.

#### Flowering time.

December–February.

#### Habitat.

Regularly occurs in marshy places. Grows also in well-drained habitats, often amongst rocks on grassy hillsides.

#### Diagnostic characters.

*Aloe cooperi* is distinguished from other grass aloes in KwaZulu-Natal with strongly keeled leaves (*Aloe myriacantha* and *Aloe sharoniae*) by the inflorescence (0.4–1.0 m high) that can sometimes be shorter than the distichous leaves (40–80 cm long). Leaves have copious white spots near the base on the lower surface and a toothed margin. Flowers are salmon-pink near the base, green tipped and 25–40 mm long, with the mouth not bilabiate or upturned. Floral bracts are flat and not clasping the pedicel (as in *Aloe sharoniae*).

#### Conservation status.

Least Concern, but declining. Threats include habitat transformation owing to commercial silvicultural and agricultural practices, as well as overgrazing and alien invasives (Raimondo et al. 2009).

#### Distribution.

**Figure 11.**
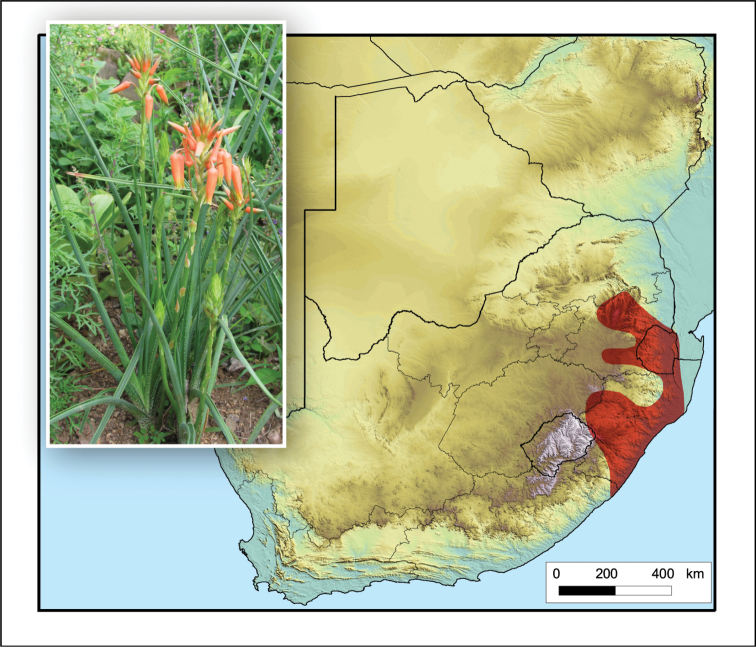
*Aloe cooperi*. Photo: N.R. Crouch.

Occurs mainly in KwaZulu-Natal and Mpumalanga, just entering the eastern Free State, the southeast of Limpopo and the northern part of the Eastern Cape in South Africa, also widespread in Eswatini and just entering Lesotho and Mozambique (Fig. 11).

#### Notes.

In recent years, *Aloe cooperi* has become very popular in South Africa in large-scale landscaping, for example of industrial sites. Unlike several other grass and slender aloes, that do not thrive beyond their natural geographical distribution ranges, most forms of *Aloe cooperi* are relatively easy in cultivation.

### ^NE^ Aloe dewetii

Taxon classificationPlantaeAsparagalesAsphodelaceae

Reynolds

4FCD9F3C-49AC-5EF9-9FA7-9E4DA97F8A93

#### Common names.

De Wet’s aloe (English); dewetse-aalwyn (Afrikaans).

#### Description.

Acaulescent plants, 0.5–0.8 m high; rosettes solitary, erect, can be over 1 m in diameter. ***Leaves*** densely rosulate, speading, dull glossy green, upper surface with numerous dull white, elongate, spots, irregularly scattered or sometimes in irregular undulating transverse bands, lower surface without spots, obscurely lineate, lanceolate-attenuate, 36–50 cm long, 7–13 cm wide at base; margin prominent, horny, brown, with pungent, deltoid, stout, brown teeth, up to 10 mm long, 10–15 mm apart; exudate clear. ***Inflorescence*** up to 2–3 m high, erect, 8- to 12-branched from about middle. **Racemes** cylindrical-acuminate, up to 40 cm long, ± 7 cm wide, lax, terminal raceme the longest. ***Floral bracts*** ± 20 mm long, 3 mm wide. ***Pedicels*** 8–15 mm long. **Flowers**: *perianth* dull scarlet with a bloom, 35–42 mm long, up to 14 mm across ovary, abruptly constricted above ovary to form distinct globose basal swelling, enlarging towards mouth, slightly decurved; outer segments free for 6 mm; *stamens* exserted to 3 mm; *style* exserted 1–2 mm.

#### Flowering time.

February–March.

#### Habitat.

Windswept, gently sloping open grassland in midlands of the province on heavy soils, in areas with fairly cold winters and high rainfall with a summer maximum.

#### Diagnostic characters.

*Aloe dewetii* can be distinguished from other maculate aloes in KwaZulu-Natal (Aloe maculata subsp. maculata, *Aloe mudenensis*, *Aloe parvibracteata*, *Aloe prinslooi*, *Aloe pruinosa*, *Aloe suffulta*, *Aloe umfoloziensis*, *Aloe vanrooyenii* and *Aloe viridiana*) by the spreading leaves (36–50 × 7–13 cm) that have a peculiar glossy appearance and a most pronounced horny, brown margin with extra-large, pungent teeth of up to 10 mm long. Leaves are spotted on the upper surface, while the lower surface is without spots and obscurely lineate. The 8- to 12-branched and rebranched inflorescences are the tallest of all the maculates (up to 2–3 m high) and have widely-spreading branches and long cylindrical, lax racemes (up to 40 cm long). Pedicels are 8–15 mm long. Flowers are dull scarlet with a bloom, 35–42 mm long and with a large globose basal swelling (up to 14 mm diameter).

#### Conservation status.

Least Concern (Raimondo et al. 2009).

#### Distribution.

**Figure 12.**
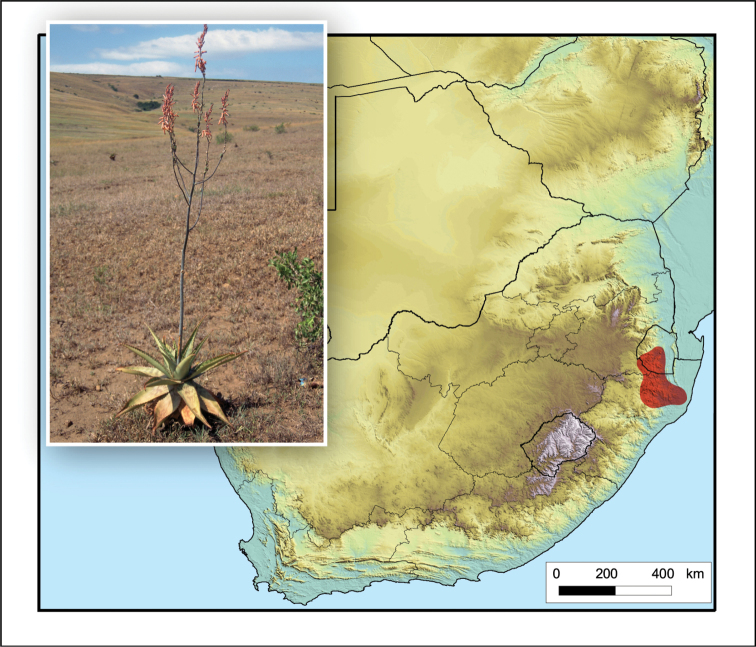
*Aloe dewetii*. Photo: N.R. Crouch.

Limited to northern KwaZulu-Natal and southern Mpumalanga in South Africa, as well as Eswatini (Fig. 12).

### ^NE^ Aloe dominella

Taxon classificationPlantaeAsparagalesAsphodelaceae

Reynolds

9595D73F-F049-56A9-911D-5606D3CFAA7C

#### Description.

Grass aloe, 0.3–0.4 m high. ***Stem*** up to 0.15 m, branched, suckering to form clumps, erect, with persistent dried leaves. ***Leaves*** rosulate, stiffly erect, dull green, upper surface without spots, lower surface with numerous small white spots near base, narrowly linear-lanceolate, attenuate, 7–35 cm long, 0.2–1.0 cm wide, widening to ± 25 mm at sheathing base; margin very narrow, white, cartilaginous, with firm white teeth, 0.5–1.0 mm long, 2–5 mm apart; exudate clear. ***Inflorescence*** 0.25–0.40 m high, erect, simple. ***Raceme*** capitate, ± 4 cm long, ± 8 cm wide, rather dense. ***Floral bracts*** up to 15 mm long, 3–4 mm wide. ***Pedicels*** 13–20 mm long. ***Flowers***: *perianth* lemon-yellow, 13–18 mm long, 4–5 mm across ovary, widening slightly towards mouth, cylindrical-trigonous, slightly clavate; outer segments free to base; *stamens* exserted 3–4 mm; *style* exserted to 7 mm.

#### Flowering time.

June–October.

#### Habitat.

Wedged between rocks in short grassland, often on steep, dry, rocky slopes.

#### Diagnostic characters.

*Aloe dominella* can be distinguished from other grass aloes in KwaZulu-Natal with unkeeled leaves that are usually narrower than 3.5 cm and that lack a bulb-like underground swelling (*Aloe linearifolia*, *Aloe micracantha*, *Aloe minima*, *Aloe nicholsii*, *Aloe parviflora* and *Aloe saundersiae*), by the rosulate, very narrow leaves (7–35 × 0.2–1.0 cm) that are stiffly erect in small tufts. The dull green leaves have numerous small white spots near the base on the lower surface. It is also characterised by the unbranched inflorescences (0.25–0.40 m high) with short, yellow flowers (13–18 mm long) that are carried in rather dense capitate racemes. Pedicels are 13–20 mm long. Rosettes are in groups.

#### Conservation status.

Near-threatened. Threats include overgrazing, alien invasives and poor recruitment owing to too frequent fires (Raimondo et al. 2009).

#### Distribution.

**Figure 13.**
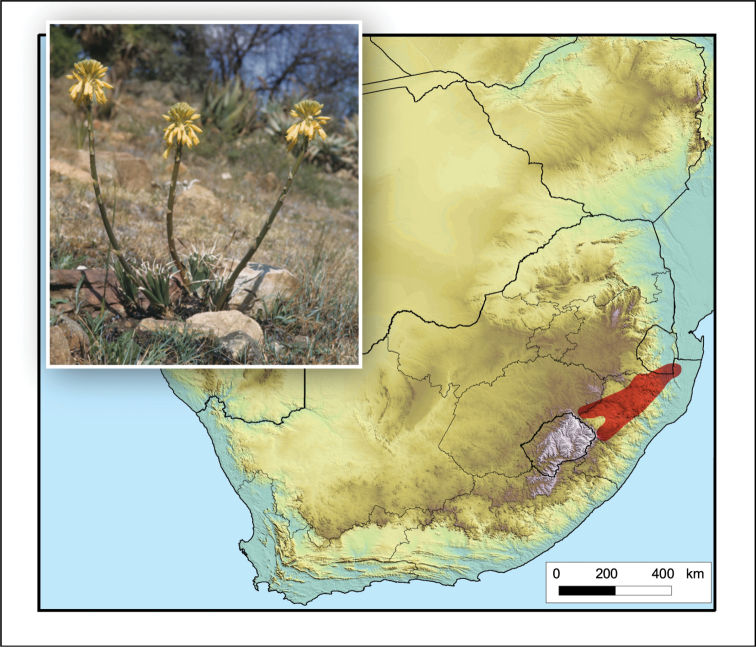
*Aloe dominella*. Photo: SANBI, PRE Slide Collection.

Confined to the central highlands of KwaZulu-Natal in South Africa, from Estcourt to Vryheid; just entering southern Eswatini (Fig. 13).

### Aloe ecklonis

Taxon classificationPlantaeAsparagalesAsphodelaceae

Salm-Dyck

19D6FE5C-B2D4-5B6B-85D1-BEFF3AA46846

#### Common names.

Ecklon’s aloe (English); ecklonse-aalwyn, vlei-aalwyn (Afrikaans); isipukutwane, isiphuthumane (Zulu).

#### Description.

Grass aloe. Acaulescent plants or ***stem*** very short; rosettes solitary or in small groups. ***Leaves*** rosulate, deciduous, erectly spreading, dull green, without spots, sometimes with few small, white spots near base on lower surface, lanceolate-attenuate, 30–40 cm long, 3–10 cm wide at base; margin with firm, white, deltoid teeth, 1–3 mm long, 3–5 mm apart; exudate clear. ***Inflorescences*** 0.38–0.50 m high, erect, simple. ***Raceme*** broadly capitate, somewhat corymbose, ± 5 cm long, dense. ***Floral bracts*** 10–15 mm long, 3–9 mm wide. ***Pedicels*** 30–40 mm long. ***Flowers***: *perianth* yellow to red, usually salmon pink, 20–24(–40) mm long, ± 7 mm across ovary, markedly swollen in middle, narrowing towards slightly upturned mouth, basally stipitate and narrowing into pedicel, cylindrical trigonous; outer segments free almost to base; *stamens* exserted to 3 mm; *style* exserted to 5 mm.

#### Flowering time.

November–February.

#### Habitat.

Usually on heavy clay soils which pack hard on drying. Flat to undulating grassland, rarely on rocky slopes.

#### Diagnostic characters.

*Aloe ecklonis* can be distinguished from other grass aloes in KwaZulu-Natal with unkeeled leaves that are wider than 3.5 cm (*Aloe boylei*, *Aloe hlangapies*, *Aloe kraussii* and *Aloe neilcrouchii*), by the large rosettes of erectly spreading, rosulate leaves (30–40 × 3–10 cm), that sometimes have a few small, white spots near the base on the lower surface. It is further characterised by the unbranched inflorescences (0.38–0.50 m high) that have dense, broadly capitate, somewhat corymbose racemes (± 5 cm long) with short [20–24(–40) mm long], yellow to red or usually salmon-pink flowers that are markedly swollen in the middle.

#### Conservation status.

Least Concern (Raimondo et al. 2009).

#### Distribution.

**Figure 14.**
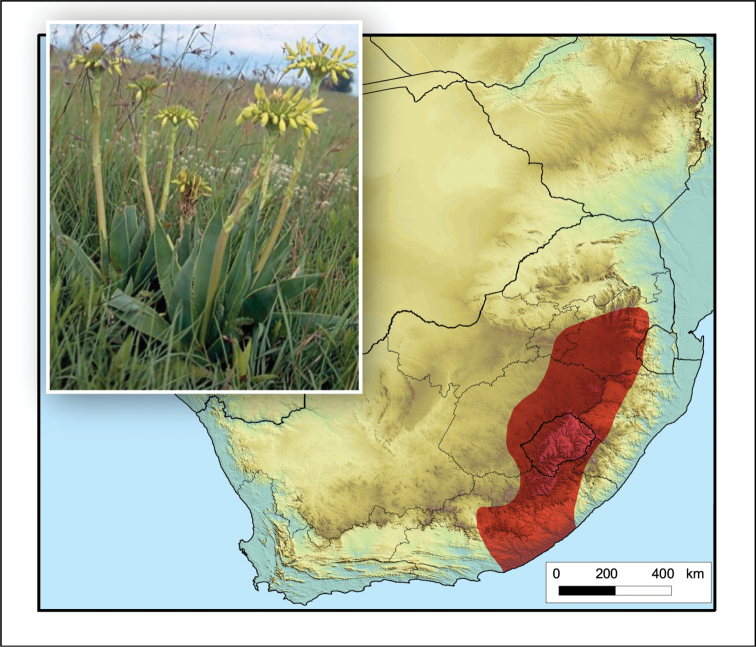
*Aloe ecklonis*. Photo: R.R. Klopper.

This species is the most widely distributed grass aloe in southern Africa, occurring along the Great Escarpment in the Eastern Cape, KwaZulu-Natal, eastern Free State, Gauteng and Mpumalanga, South Africa, as well as in Lesotho and western Eswatini (Fig. 14).

#### Note.

*Aloe ecklonis* is highly variable across its range.

### ^E^ Aloe gerstneri

Taxon classificationPlantaeAsparagalesAsphodelaceae

Reynolds

FD6FD78B-0EE9-5E4D-9839-EE89FA3AC92C

#### Common names.

Gerstner’s aloe (English); bergaalwyn (Afrikaans); isihlabane (Zulu).

#### Description.

Acaulescent plants or ***stem*** short; rosettes solitary, erect, 0.4–0.7 m high. ***Leaves*** densely rosulate, arcuate-erect, dull grey-green, without spots, texture smooth, lanceolate-ensiform, 40–60 cm long, 6–12 cm wide at base, lower surface sometimes with few spines in median line near apex (can be copiously spiny on both surfaces in young plants); margin not distinctly coloured, with isolated, pungent, deltoid, pale brown teeth from white sub-tuberculate base, 4 mm long, 10–15 mm apart; exudate honey-coloured. ***Inflorescence*** 1.0–1.3 m high, erect, simple in young plants, 1- to 3-branched from below middle in mature plants. ***Racemes*** cylindrical, slightly acuminate, up to 36 cm long, 6–7 cm wide, very dense; buds and flowers pendent. ***Floral bracts*** 18 mm long, 5 mm wide. ***Pedicels*** 5 mm long. ***Flowers***: *perianth* reddish-orange in bud, flowers yellowish-orange, 24–30 mm long, ± 7 mm across ovary, narrowing slightly towards mouth, cylindrical-ventricose, slightly clavate, mouth slightly upturned; outer segments free for 15–17 mm; *stamens* exserted to 13 mm; *style* exserted to 14 mm.

#### Flowering time.

February–March.

#### Habitat.

Rocky slopes in grassland in areas with cold winters and reasonably high rainfall, on granite or quartzite formations.

#### Diagnostic characters.

*Aloe gerstneri* can be distinguished from other virtually acaulescent, non-maculate aloes in KwaZulu-Natal (*Aristaloe aristata*, Aloe chabaudii var. chabaudii, *Aloe pratensis*, Aloe reitzii var. vernalis, *Aloe suprafoliata* and *Aloe vanbalenii*) by the very dense racemes (up to 36 × 6–7 cm) with short erect pedicels (5 mm). Flowers are yellowish-orange, 24–30 mm long, tubular and straight, pointing downwards and almost pressed against the stalk, with conspicuously exserted stamens and style. Leaves can be copiously spiny on both surfaces in young plants, but mature leaves (40–60 × 6–12 cm) are without surface prickles (sometimes with spines on median line of lower surface), arcuate erect, dull grey-green and with pungent marginal teeth on a distinctive white base.

#### Conservation status.

Endangered. Threats include habitat degradation owing to erosion caused by overgrazing and subsistence farming (Raimondo et al. 2009, L. von Staden pers. comm.).

#### Distribution.

**Figure 15.**
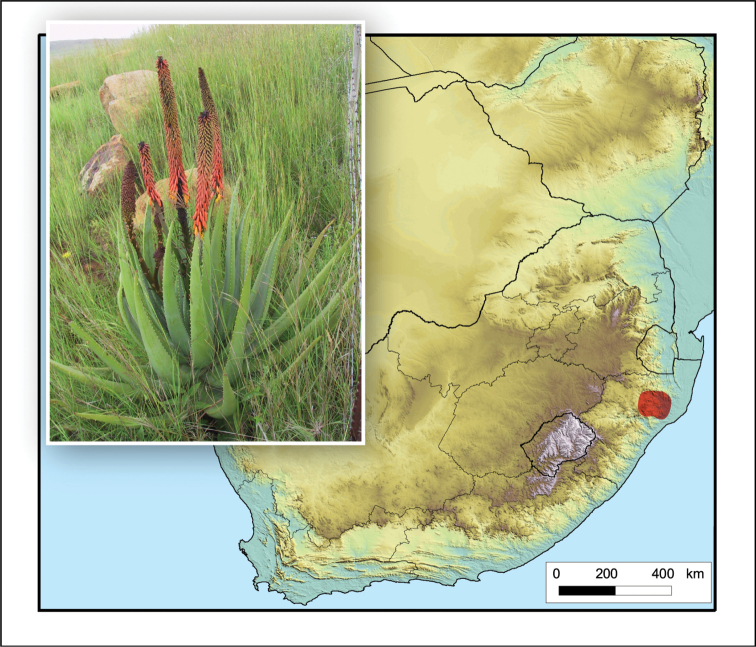
*Aloe gerstneri*. Photo: E. van Wyk.

Restricted to a small area in northern KwaZulu-Natal, South Africa (Fig. 15).

### ^NE^ Aloe hlangapies

Taxon classificationPlantaeAsparagalesAsphodelaceae

Groenew.

CF1CCA0B-1EE3-520E-972F-0D0AD1B137C5

#### Description.

Grass aloe. Acaulescent plants or ***stem*** short, up to 0.15 m; rosettes usually solitary or suckering to form small groups; with persistent dried leaves. ***Leaves*** distichous, deciduous, erect to spreading, dull green, upper surface usually without spots, sometimes sparingly spotted, lower surface usually copiously white-spotted near base, lorate-acuminate, 35–50 cm long, 5–6 cm wide; margin with soft, white teeth, ± 0.5 mm long, 5–15 mm apart; exudate clear. ***Inflorescence*** ± 0.5 m high, erect, simple. ***Raceme*** capitate, up to 7 cm long, 9–10 cm wide, dense. ***Floral bracts*** 15 mm long, 7 mm wide. ***Pedicels*** ± 25 mm long. ***Flowers***: *perianth* apricot-yellow, only rarely red or yellow, greenish tipped, 28–30 mm long, 8–10 mm across ovary, slightly widening towards middle, narrowing towards mouth, base tapering into pedicel, straight, cylindrical; outer segments free for 23–25 mm; *stamens* and *style* exserted to 1 mm.

#### Flowering time.

October–November.

#### Habitat.

Damp, low-lying grassland and on grassy slopes.

#### Diagnostic characters.

*Aloe hlangapies* can be distinguished from other grass aloes in KwaZulu-Natal with unkeeled leaves that are wider than 3.5 cm (*Aloe boylei*, *Aloe ecklonis*, *Aloe kraussii* and *Aloe neilcrouchii*), by the rosette of erect to spreading, distichous leaves (35–50 × 5–6 cm), with the upper surface usually without spots and the lower surface usually copiously white-spotted near the base. It is further characterised by the unbranched inflorescences (± 0.5 m high) that have dense, capitate racemes (up to 7 cm long) with relatively long (28–30 mm long), usually apricot-yellow and greenish tipped, tubular flowers.

#### Conservation status.

Vulnerable. Threats include habitat loss owing to silviculture, agriculture and urban expansion, as well as overgrazing and alien invasives. There is also a potential threat from coal mining (L. von Staden pers. comm.).

#### Distribution.

**Figure 16.**
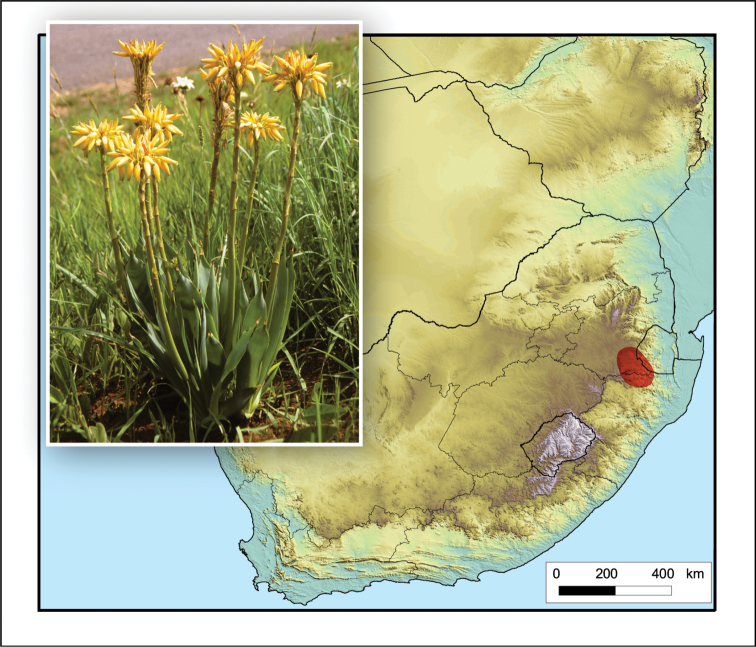
*Aloe hlangapies*. Photo: J.E. Burrows.

Only known from the area on the border between KwaZulu-Natal and Mpumalanga in South Africa and just entering south-western Eswatini (Fig. 16).

#### Notes.

Near Wakkerstroom and Volksrust in KwaZulu-Natal, *Aloe hlangapies* merges into intermediates with *Aloe ecklonis* Salm-Dyck (Reynolds 1950).

### ^E^ Aloe inconspicua

Taxon classificationPlantaeAsparagalesAsphodelaceae

Plowes

CC83E89A-B49D-5F61-8580-B4AFF1FDE70E

#### Description.

Grass aloe. Acaulescent plants, rosettes solitary, erect, with old persistent leaf bases forming a subterranean ovoid bulb-like swelling. ***Leaves*** rosulate, erect, deciduous, dark green, upper surface without spots, lower surface with narrow elongate white spots in basal half, narrowly linear, acuminate, 10–20 cm long, 0.3–0.4 cm wide, dilating below ground to 3 cm; margin narrow, translucent, with soft translucent teeth, 0.5 mm long, 2–4 mm apart. ***Inflorescence*** ± 0.15 m high, erect, simple. ***Raceme*** narrowly triangular to cylindrical, ± 7 cm long, 2 cm wide, very dense. ***Floral bracts*** 13–15 mm long. ***Pedicels*** absent. ***Flowers***: *perianth* green, 15 mm long, narrowing slightly towards slightly bilabiate mouth, cylindrical-trigonous; outer segments free to base; *stamens* exserted to 1 mm; *style* not exserted.

#### Flowering time.

November.

#### Habitat.

In sparse short grass in areas of dry, low-altitude, thorny, open woodland. Grows in the transition zone between open grassland and valley bushveld. Shale and sandstone. Hot summers, but can be very cold in winter.

#### Diagnostic characters.

*Aloe inconspicua* can be distinguished from other grass aloes in KwaZulu-Natal where the leaf bases form a subterranean bulb-like swelling (*Aloe bergeriana*, *Aloe kniphofioides* and *Aloe modesta*), by the very narrow leaves (10–20 × 0.3–0.4 cm) that are heavily spotted on the lower surface and with soft transluscent marginal teeth. It is also characterised by the very dense, unbranched, cylindrical raceme (± 7 cm long) with sessile, suberectly spreading, green, slightly bilabiate, unscented flowers (15 mm long).

#### Conservation status.

Endangered. Threats include habitat degradation owing to overgrazing, subsistence farming and urban expansion (Raimondo et al. 2009).

#### Distribution.

**Figure 17.**
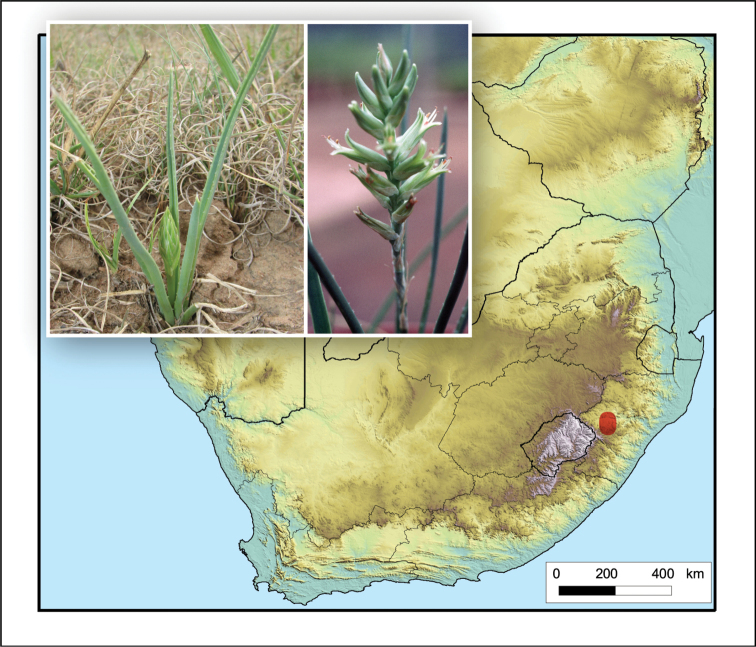
*Aloe inconspicua*. Photos: P. Joffe (flowers), E. van Wyk (plant).

Only known from the Bushmans River catchment between Weenen and Estcourt in KwaZulu-Natal, South Africa (Fig. 17).

### Aloe kniphofioides

Taxon classificationPlantaeAsparagalesAsphodelaceae

Baker

B514473A-D573-533A-B6CF-38850252112B

#### Common names.

Grass aloe (English); grasaalwyn (Afrikaans).

#### Description.

Grass aloe. Acaulescent plants; rosettes solitary, leaf bases forming bulb-like underground swelling. ***Leaves*** rosulate, erect, green, without spots, narrowly linear, 20–40 cm long, 0.6–0.7 cm wide, dilating below ground-level to 2.0–3.0 cm wide; margin entire or minutely dentate, with small, white teeth, ± 0.7 mm long, 1–2 mm apart, more crowded lower down; exudate clear. ***Inflorescence*** up to 0.30–0.55 m high, erect, simple. ***Raceme*** cylindrical, 10–15 cm long, very lax, few-flowered. ***Floral bracts*** 15–22 mm long, 4–7 mm wide. ***Pedicels*** 12–18 mm long. ***Flowers***: *perianth* pale pink to scarlet, green-tipped, 30–50 mm long, base rounded, 6–7 mm across ovary, not narrowed above ovary, cylindrical, slightly curved; outer segments free for 6–8 mm; *stamens* and *style* not or very shortly exserted to 1 mm.

#### Flowering time.

October–November.

#### Habitat.

Grassland in reasonably high rainfall areas. Rather heavy, stone-free soils.

#### Diagnostic characters.

*Aloe kniphofioides* can be distinguished from other grass aloes in KwaZulu-Natal where the leaf bases form a subterranean bulb-like swelling (*Aloe bergeriana*, *Aloe inconspicua* and *Aloe modesta*), by the long, narrow, bright red, unscented flowers (30–50 mm long) that are pendent in a very lax, unbranched, cylindrical raceme (10–15 cm long), with pedicels 12–18 mm long. The narrow leaves (20–40 × 0.6–0.7 cm) are usually without spots and with or without minute white marginal teeth.

#### Conservation status.

Near-threatened. Threats include habitat transformation and degradation owing to mining, commercial afforestation and alien invasives, as well as a loss of pollinators and poor fire management leading to poor recruitment (Raimondo et al. 2009, L. von Staden pers. comm.).

#### Distribution.

**Figure 18.**
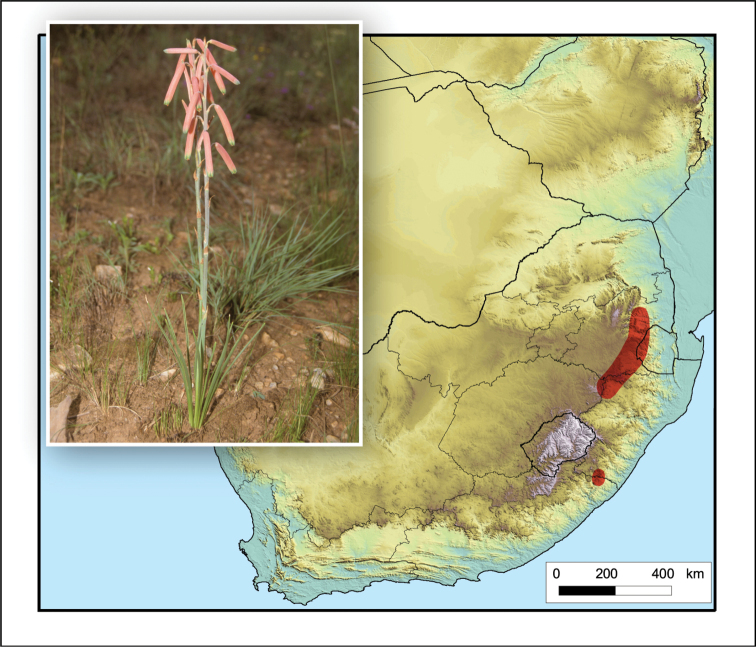
*Aloe kniphofioides*. Photo: J.E. Burrows.

Widely but sparsely distributed. This species has a disjunct distribution: it occurs in the Kokstad area on the border of KwaZulu-Natal and the Eastern Cape province; and then along the Great Escarpment in northern KwaZulu-Natal, Mpumalanga and just entering the eastern Free State, South Africa, as well as in Eswatini (Fig. 18).

### ^E^ Aloe kraussii

Taxon classificationPlantaeAsparagalesAsphodelaceae

Baker

A2B17A14-10E1-539B-9A2B-E3F5104AF1CD

#### Common names.

Broad-leaved yellow grass aloe (English); isipukutwane, isiputumane (Zulu).

#### Description.

Grass aloe. Acaulescent plants or ***stem*** very short, dried leaves not persistent; rosettes solitary or suckering to form small groups. ***Leaves*** distichous or sub-distichous, becoming rosulate in old plants, deciduous, erectly spreading, dull green, usually without spots, lower surface sometimes with few white spots near base, broadly linear-acuminate, ± 30–40 cm long, 3.5–5.0 cm wide; margin extremely narrow, white, cartilaginous, with minute white teeth; exudate clear. ***Inflorescence*** 0.35–0.40 m high, erect, simple. ***Raceme*** capitate, somewhat corymbose, ± 3 cm long, ± 10 cm wide, dense. ***Floral bracts*** up to 15 mm long, 5 mm wide. ***Pedicels*** up to 35 mm long. ***Flowers***: *perianth* lemon-yellow to yellow, green-tipped, 16–18 mm long, ± 6 mm across ovary, narrowing towards slightly upturned mouth, base tapering into pedicel, straight, cylindrical; outer segments free almost to base; *stamens* exserted to 3 mm; *style* exserted to 5 mm.

#### Flowering time.

November–February.

#### Habitat.

Damp places in sandy soil or on stony slopes of grassy hillsides in the mistbelt of the KwaZulu-Natal midlands.

#### Diagnostic characters.

*Aloe kraussii* can be distinguished from other grass aloes in KwaZulu-Natal with unkeeled leaves that are wider than 3.5 cm (*Aloe boylei*, *Aloe ecklonis*, *Aloe hlangapies* and *Aloe neilcrouchii*), by the rosettes of erectly spreading, distichous or sub-distichous leaves (± 30–40 × 3.5–5.0 cm), that sometimes have a few white spots near the base on the lower surface. It is further characterised by the unbranched inflorescences (0.35–0.40 m high) that have dense, capitate and somewhat corymbose racemes (± 3 cm long) with small (16–18 mm long), yellow, rather straight flowers.

#### Conservation status.

Endangered. Threats include habitat loss and degradation owing to silviculture, agiculture (mainly sugarcane) and urban expansion, as well as alien invasives (L. von Staden pers. comm.). It is one of the rarer of the grass aloes owing to habitat loss (Craib 2005).

#### Distribution.

**Figure 19.**
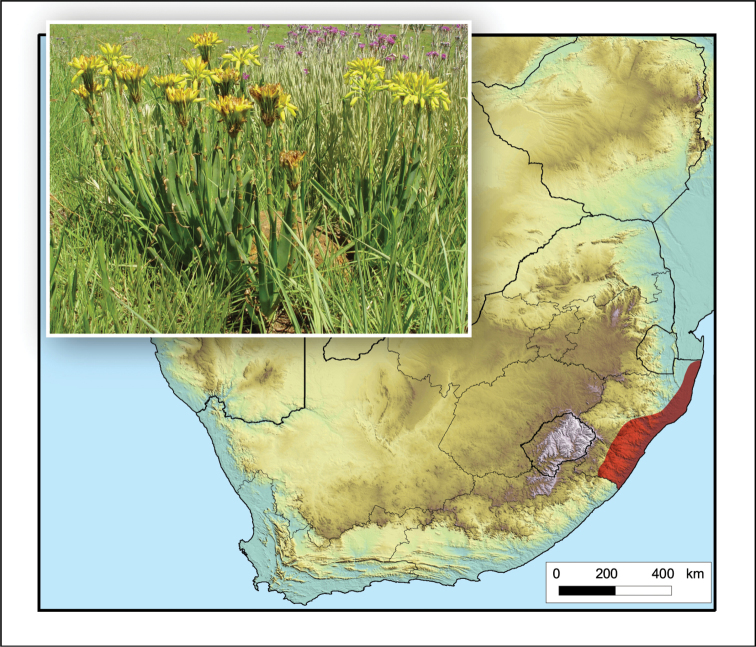
*Aloe kraussii*. Photo: G. Nichols.

Confined to the coastal areas of KwaZulu-Natal, South Africa, where it is still fairly common (Fig. 19).

#### Notes.

*Aloe kraussii* is considered by some to be a low-altitude form of *Aloe ecklonis* Salm-Dyck (Carter et al. 2011).

### ^NE^ Aloe linearifolia

Taxon classificationPlantaeAsparagalesAsphodelaceae

A.Berger

42F0791B-AA40-5E6E-A61B-94459228D328

#### Common names.

Dwarf yellow grass aloe (English); inkuphuyana (Zulu).

#### Description.

Grass aloe, up to ± 0.3 m high. Acaulescent plants or ***stem*** very short, usually simple, occasionally 1- or 2-branched at ground level, erect. ***Leaves*** usually distichous, rarely rosulate, erect to erectly spreading, deciduous, green, with copious white and brown spots near base on lower surface, linear, ± 25 cm long, 0.5–1.0 cm wide, basal portion dilating and becoming amplexicaul; margin usually minutely dentate near base, teeth up to 0.5 mm, up to 4 mm apart, without teeth towards tip; exudate clear. ***Inflorescence*** 0.20–0.35 m high, erect, simple. ***Racemes*** capitate, ± 2 cm long, dense. ***Floral bracts*** 10–14 mm long, 4–7 mm wide. ***Pedicels*** 12–15 mm long. ***Flowers***: *perianth* greenish-yellow to yellow, ± 12 mm long, ± 4.5 mm across ovary, base tapering into pedicel, not narrowed above ovary, mouth slightly upturned, cylindrical-trigonous; outer segments free almost to base; *stamens* exserted 0–2 mm; *style* exserted 1–2 mm.

#### Flowering time.

January–February (March).

#### Habitat.

Damp places in open sunny situations in stony grassveld or on grassy slopes, often on rocky outcrops.

#### Diagnostic characters.

*Aloe linearifolia* can be distinguished from other grass aloes in KwaZulu-Natal with unkeeled leaves that are usually narrower than 3.5 cm and that lack a bulb-like underground swelling (*Aloe dominella*, *Aloe micracantha*, *Aloe minima*, *Aloe nicholsii*, *Aloe parviflora* and *Aloe saundersiae*), by the distichous, erect to erectly spreading, long and narrow, green leaves (± 25 × 0.5–1.0 cm), with copious white and brown spots near the base on the lower surface. It is also characterised by the unbranched inflorescences (0.20–0.35 m high) with dense, capitate racemes of short, yellow flowers (± 12 mm long) that are carried on stout pedicels (12–15 mm long). Rosettes are usually solitary or occasionally in small groups.

#### Conservation status.

Least Concern. Threats include habitat transformation owing to commercial silvicultural and agricultural practices and urban expansion, as well as overgrazing and poor fire management (Raimondo et al. 2009, L. von Staden pers. comm.).

#### Distribution.

**Figure 20.**
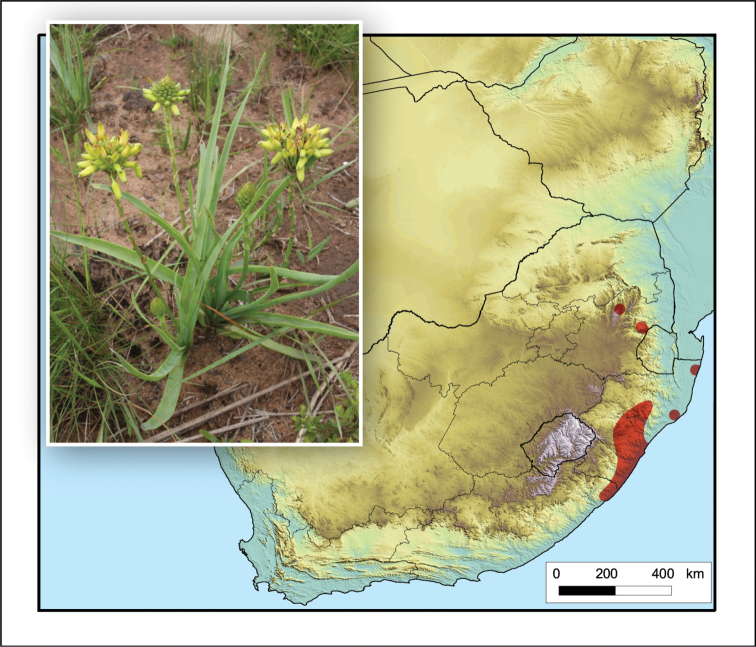
*Aloe linearifolia*. Photo: D. Styles.

Mainly found in the grasslands of southern and central KwaZulu-Natal and the northern Eastern Cape, South Africa, with a few scattered collections from northern KwaZulu-Natal and Mpumalanga of South Africa; also just entering north-western Eswatini (Fig. 20).

### Aloe maculata All. subsp. maculata

Taxon classificationPlantaeAsparagalesAsphodelaceae

6ACAA104-14D7-5237-8A35-38C8CFB203CF

#### Common names.

Common soap aloe (English); bontaalwyn (Afrikaans); amahlala, icena (isiZulu).

#### Description.

Acaulescent plants or with ***stem*** up to 0.5 m; rosettes solitary or suckering to form dense groups. ***Leaves*** densely rosulate, erectly spreading to slightly recurved, upper surface pale to darker green, with numerous, dull, white spots in irregular broken, wavy, transverse bands, lower surface paler green, obscurely lineate and usually without spots, ovate-lanceolate, up to 25–30 cm long, 8–12 cm wide, with dried twisted apex; margin with pungent, horny, brown teeth, 3–5 mm long, ± 10 mm apart; exudate clear. ***Inflorescence*** 0.4–1.0 m high, erect, branched. ***Racemes*** capitate-corymbose, 10–12 cm long, dense. ***Floral bracts*** ± 12–23 mm long, 3–5 mm wide. ***Pedicels*** 35–45 mm long. ***Flowers***: *perianth* usually salmon pink to orange, sometimes yellow or red, 35–50 mm long, up to 10 mm across ovary, abruptly constricted above ovary to form sub-globose basal swelling, enlarging towards wide open mouth, cylindrical, slightly decurved; outer segments free for 10–15 mm; *stamens* exserted 1–3 mm; *style* exserted to 5 mm.

#### Flowering time.

June–September in the north, December–January in the south.

#### Habitat.

Variety of grasslands, scrub, thicket and on rocky outcrops.

#### Diagnostic characters.

Aloe maculata subsp. maculata can be distinguished from other maculate aloes in KwaZulu-Natal (*Aloe dewetii*, *Aloe mudenensis*, *Aloe parvibracteata*, *Aloe prinslooi*, *Aloe pruinosa*, *Aloe suffulta*, *Aloe umfoloziensis*, *Aloe vanrooyenii* and *Aloe viridiana*) by its branched inflorescence (0.4–1.0 m high) with flat-topped, capitate, dense racemes (up to 10–12 × 12–16 cm) and pedicels of 35–45 mm long. Flowers are usually salmon pink to orange, sometimes yellow or red, 35–50 mm long, with a sub-globose basal swelling (up to 10 mm diameter). Leaves are spreading to slightly recurved, up to 25–30 × 8–12 cm and spotted on the upper surface, with the paler lower surface obscurely lineate and usually without spots. Marginal teeth are 3–5 mm long.

#### Conservation status.

Least Concern (Raimondo et al. 2009).

#### Distribution.

**Figure 21.**
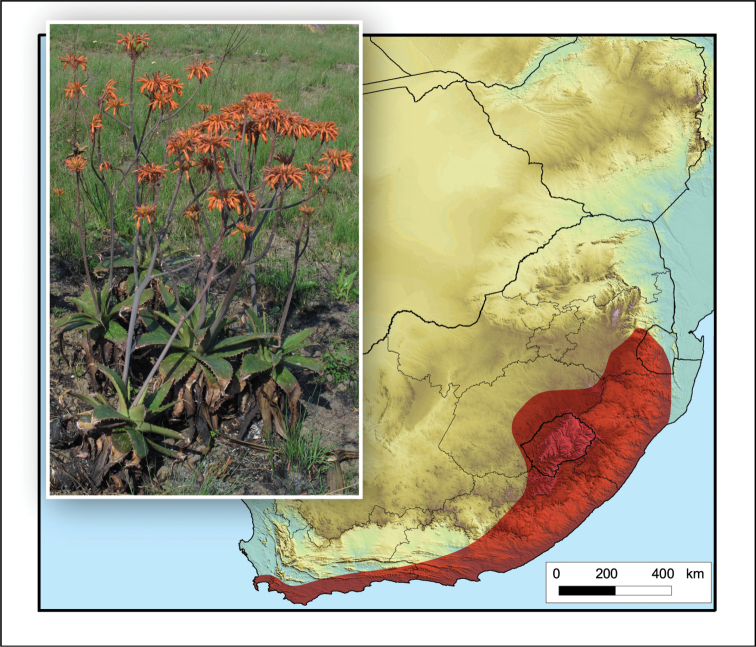
Aloe maculata subsp. maculata. Photo: N.R. Crouch.

This subspecies is one of the most widely distributed of the spotted aloes. It occurs from the Cape Peninsula through the Western and Eastern Cape, into the eastern Free State, through KwaZulu-Natal to Mpumalanga, South Africa; also in Lesotho and Eswatini (Fig. 21).

#### Notes.

One other subspecies is recognised, namely A. maculata subsp. ficksburgensis (Reynolds) Gideon F.Sm. & Figueiredo, which is only known from the eastern Free State, South Africa and western Lesotho.

### Aloe marlothii

Taxon classificationPlantaeAsparagalesAsphodelaceae

A.Berger

47D54199-3F6C-5FFB-B185-A611A045857D

#### Common names.

Mountain aloe (English); bergaalwyn, boomaalwyn, snuifaalwyn (Afrikaans); ikhala, imihlaba, inhlaba, inhlabane, umhlaba (Zulu).

**Table:** 

1	Stems simple, erect, up to 4 m high; leaves with many surface prickles; racemes 30–50 cm long; flowers 30–35 mm long	**Aloe marlothii subsp. marlothii**
–	Stems suckering to form clumps, erect or oblique to procumbent, 1.00–1.75 m high; leaves with no to few surface prickles; racemes 15–25 cm long; flowers 18–30 mm long	**Aloe marlothii subsp. orientalis Glen & D.S.Hardy**

#### Description.

Solitary, arborescent plant of up to 5–6 m high (subsp. marlothii) or 1.00–1.75 m high (subsp. orientalis). ***Stem*** simple and erect (subsp. marlothii) or often suckering to form small groups and erect or oblique to procumbent (subsp. orientalis), densely covered with persistent dried leaves. ***Leaves*** densely rosulate, suberect to spreading and eventually pendent, dull grey-green to glaucous (subsp. marlothii) or arcuate-incurved to spreading or slightly decurved, glaucous to blue-green (subsp. orientalis), without spots, with scattered, reddish-brown, pungent spines, usually more copious on lower surface (subsp. marlothii) or with no to few surface prickles (subsp. orientalis), lanceolate-attenuate, 100–150 cm long, 20–25 cm wide at base (subsp. marlothii) or 75–150 cm long, 8–25 cm wide at base (subsp. orientalis); margin with stout, pungent, reddish-brown teeth, 3–6 mm long, 10–20 mm apart (subsp. marlothii) or 3–4 mm long, 8–25 mm apart (subsp. orientalis); exudate honey-coloured. ***Inflorescence*** up to 0.8 m high, 10- to 30-branched from below middle, lower branches rebranched, branches horizontal to spreading. ***Racemes*** cylindrical, 30–50 cm long, horizontal to suboblique (subsp. marlothii) or 15–25 cm long, spreading to rarely erect (subsp. orientalis), dense; flowers markedly secund when open. ***Floral bracts*** 8–9 mm long, 5 mm wide (subsp. marlothii) or 4–9 mm long, 2–5 mm wide (subsp. orientalis). ***Pedicels*** 5–8 mm (subsp. marlothii) or 3–5 mm (subsp. orientalis) long. ***Flowers***: *perianth* orange to yellowish-orange, 30–35 mm long, ± 7 mm wide across ovary (subsp. marlothii) or yellow to orange, 18–30 mm long (subsp. orientalis), enlarging above ovary, narrowing towards usually wide-open mouth, cylindrical-clavate to cylindrical-ventricose (subsp. marlothii) or cylindrical to ventricose (subsp. orientalis); outer segments free for 20–23 mm (subsp. marlothii) or for 6–15 mm (subsp. orientalis); *stamens* exserted to 15 mm; *style* exserted 15–20 mm (subsp. marlothii) or *stamens* and *style* exserted 8–12 mm (subsp. orientalis).

#### Flowering time.

June–August, as late as September along the Witwatersrand, Gauteng, South Africa (subsp. marlothii). July–August (subsp. orientalis).

#### Habitat.

Aloe marlothii subsp. marlothii grows in a wide variety of habitats, including bushveld on stony, usually bare soils or rocky outcrops. Aloe marlothii subsp. orientalis is confined to coastal grassland in sandy soil.

#### Diagnostic characters.

*Aloe marlothii* differs from the other tall often single-stemmed aloes in KwaZulu-Natal with branched inflorescences and persistent dried leaves (*Aloe candelabrum*, *Aloe pluridens*, *Aloe rupestris*, *Aloe spectabilis* and *Aloe thraskii*), by having its racemes horizontal to oblique (not erect) with the flowers secund. The inflorescence is much-branched with up to 30 racemes. Flowers are yellow to orange, up to 35 mm long with the inner segment tips purplish and the exserted portion of stamens deep purple (not deep purplish-black to black segment tips and orange filaments as in *Aloe spectabilis*). Aloe marlothii subsp. marlothii is further distinguished by the presence of copious spines on both leaf surfaces. The main characters separating Aloe marlothii subsp. orientalis Glen & D.S.Hardy from the typical subspecies is its short stems of up to 1.75 m (not up to 4 m), that are sometimes oblique or procumbent and often suckering to form clumps (not solitary rosettes). Its leaves also have no or only a few surface prickles, while its racemes are usually oblique (not as horizontal as in subsp. marlothii). Drying leaves of Aloe marlothii subsp. orientalis have a peculiar, unpleasant odour (Glen and Hardy 1987).

#### Conservation status.

Both subspecies are Least Concern (Raimondo et al. 2009).

**Figure 22.**
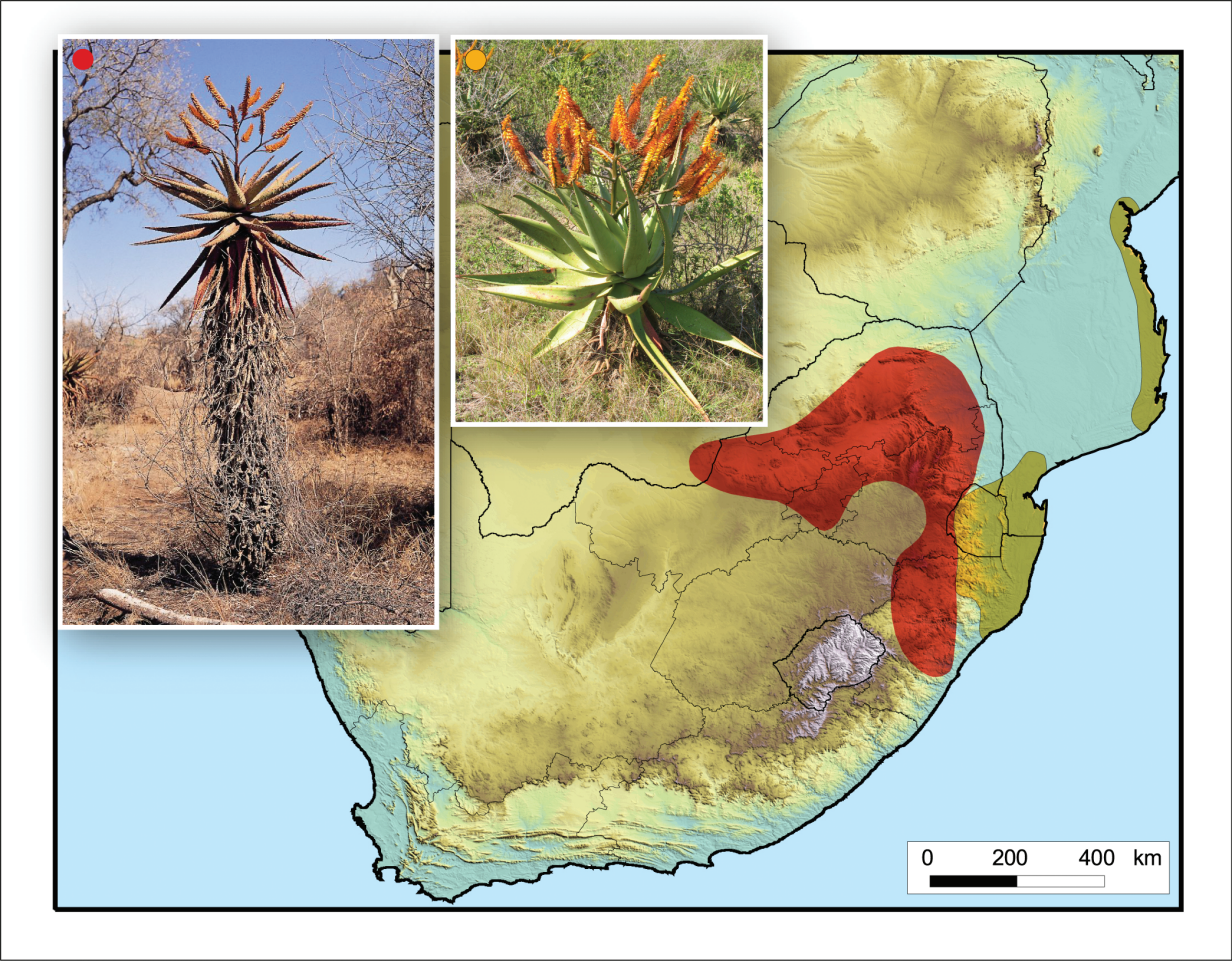
Aloe marlothii subsp. marlothii (red on map) and Aloe marlothii subsp. orientalis (orange on map). Photos: G.F. Smith (subsp. marlothii), N.R. Crouch (subsp. orientalis).

**Distribution.**Aloe marlothii subsp. marlothii (red on map) is widespread throughout North-West, Limpopo (as far north as the Soutpansberg), Mpumalanga, Gauteng and KwaZulu-Natal in South Africa, as well as in south-eastern Botswana and into western Eswatini. Aloe marlothii subsp. orientalis (orange on map) is only known from northern KwaZulu-Natal in South Africa, as well as southern Mozambique and low lying areas of Eswatini (Fig. 22). It is near-endemic to the Maputaland Centre of Endemism.

#### Notes.

*Aloe marlothii* grades through intermediates into *Aloe spectabilis* Reynolds at some localities (Reynolds 1950). As a result, the two species are considered conspecific by some authors (Glen and Hardy 2000; Carter et al. 2011).

### Aloe micracantha

Taxon classificationPlantaeAsparagalesAsphodelaceae

Haw.

4D64F3DE-B00A-5518-9A40-1C6FB7B633F2

#### Common names.

Wateraalwyn (Afrikaans).

#### Description.

Grass aloe, 0.35–0.50 m high; rosettes usually solitary. Acaulescent plants or ***stem*** very short, simple, sometimes 1- or 2-branched, erect. ***Leaves*** multifarious, sub-distichous in young plants, erect, rigid, deep green to yellowish-green, with copious white, subtuberculate and subspinulescent spots especially on lower surface towards base, deeply channelled on upper surface, narrowly deltoid to linear, acuminate, 30–50 cm long, 2–4 cm wide at base; margin with firm, white teeth, up to 2 mm long, 1–3 mm apart; exudate clear. ***Inflorescence*** 0.45–0.50 m high, erect, simple. ***Raceme*** capitate, somewhat corymbose, ± 8 cm long, dense. ***Floral bracts*** ± 35 mm long, 5–7 mm wide. ***Pedicels*** 20–35 mm long. ***Flowers***: *perianth* salmon-pink to reddish-orange, 25–40 mm long, slightly constricted above ovary, slightly widening towards wide open mouth, basally stipitate, straight, cylindrical-trigonous; outer segments free to base; *stamens* exserted 0–1 mm; *style* exserted 1–2 mm.

#### Flowering time.

January–March.

#### Habitat.

Well-drained, dry, open sandy or stony places in coastal grassland, often wedged between rocks.

#### Diagnostic characters.

*Aloe micracantha* can be distinguished from other grass aloes in KwaZulu-Natal with unkeeled leaves that are usually narrower than 3.5 cm and that lack a bulb-like underground swelling (*Aloe dominella*, *Aloe linearifolia*, *Aloe minima*, *Aloe nicholsii*, *Aloe parviflora* and *Aloe saundersiae*), by the multifarious to sub-distichous, erect and rigid, deep yellowish-green, smoothly channelled (not sharply keeled) leaves (30–50 × 2–4 cm), with copious white spots on both surfaces. It is also characterised by the unbranched inflorescences (0.45–0.50 m high) with dense, capitate racemes, where the pedicels (20–35 mm long), perianth (25–40 mm long) and ovary are all salmon-pink. Rosettes are usually solitary.

#### Conservation status.

Near-threatened. Threats include agricultural practices, urban expansion and the encroachment of alien invasives (Raimondo et al. 2009).

#### Distribution.

**Figure 23.**
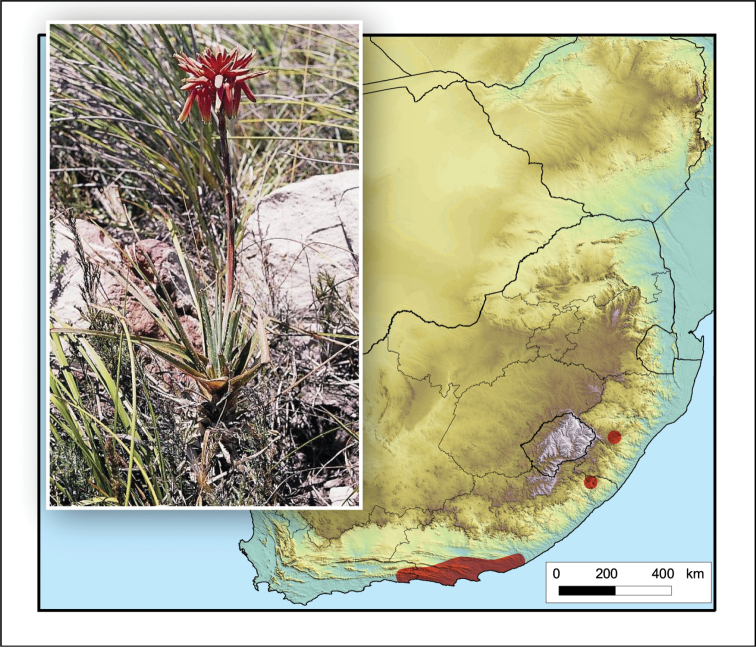
*Aloe micracantha*. Photo: G.F. Smith.

Occurs in a fairly narrow coastal to near-coastal strip from the Uniondale area in the Western Cape to Bathurst in the Eastern Cape, with outlier collections from Mt Ayliff in the north-eastern Eastern Cape and Karkloof in KwaZulu-Natal, South Africa (Fig. 23). It is the grass aloe with the most westerly distribution in southern Africa and the only grass aloe to occur in Fynbos vegetation.

### Aloe minima

Taxon classificationPlantaeAsparagalesAsphodelaceae

Baker

C44A0AC6-FE62-54BF-B0EE-35B4C145E68F

#### Common names.

Pink grass aloe (English); isipukushane, isipukutwane, isiputuma, isiphuthumane, isiphukhutshane (Zulu).

#### Description.

Grass aloe, 0.2–0.3 m high. Acaulescent plants; rosettes single, erect. ***Leaves*** rosulate, suberect, rather rigid, green, lower surface with copious slightly tuberculate-spinulescent spots towards base, linear, 25–35 cm long, 0.4–0.6 cm wide; margin ciliate, with minute whitish teeth in lower half, up to 0.5 mm long, 1–2 mm apart; exudate clear. ***Inflorescence*** 0.25–0.50 m high, erect, simple. ***Raceme*** capitate, ± 3 cm long, dense. ***Floral bracts*** 5–12 mm long, 3–5 mm wide. ***Pedicels*** 10–20 mm long. ***Flowers***: *perianth* dull pink, 10–11 mm long, ± 3–4 mm across ovary, narrowing towards upturned mouth, basally stipitate, cylindric-trigonous; outer segments free to base; *stamens* and *stigma* not or only slightly exserted.

#### Flowering time.

January–February.

#### Habitat.

Hilly and mountainous grassland on fairly heavy soils with loose stones.

#### Diagnostic characters.

*Aloe minima* can be distinguished from other grass aloes in KwaZulu-Natal with unkeeled leaves that are usually narrower than 3.5 cm and that lack a bulb-like underground swelling (*Aloe dominella*, *Aloe linearifolia*, *Aloe micracantha*, *Aloe nicholsii*, *Aloe parviflora* and *Aloe saundersiae*), by the short inflorescence (0.25–0.50 m high) with dense, capitate racemes and pedicels 10–20 mm long. The peduncle is smooth (without prickles as in *Aloe parviflora*). Flowers are small (10–11 mm long) and dull pink, with a spreading, upturned mouth. The rosulate leaves (25–35 × 0.4–0.6 cm) are suberect and rather rigid, with copious slightly tuberculate-spinulescent spots towards the base on the lower surface. Rosettes are solitary.

#### Conservation status.

Least Concern (Raimondo et al. 2009).

#### Distribution.

**Figure 24.**
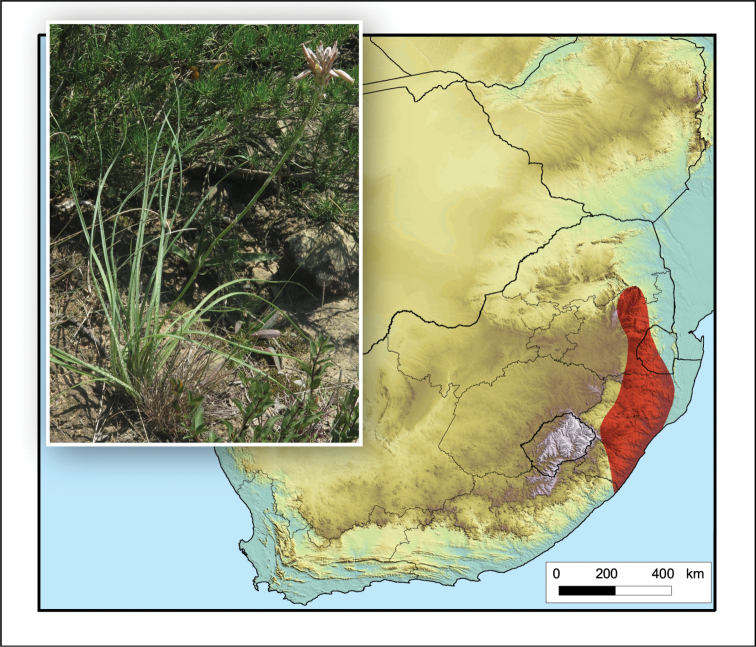
*Aloe minima*. Photo: N.R. Crouch.

Occurs from the far northern parts of the Eastern Cape, just south of Port Edward, widespread through KwaZulu-Natal and along the Great Escarpment into Mpumalanga, South Africa and western Eswatini (Fig. 24).

### Aloe modesta

Taxon classificationPlantaeAsparagalesAsphodelaceae

Reynolds

C4DA128E-11C6-5604-8B34-BDAEF7418A58

#### Common names.

Vlei aloe (English); vlei-aalwyn (Afrikaans).

#### Description.

Grass aloe. Acaulescent plants; rosettes solitary, erect, 0.2–0.3 m high; leaf bases forming bulb-like swelling underground. ***Leaves*** rosulate, erect, dull deep green, upper surface without spots, lower surface copiously white-spotted near base, linear-acute, slightly channelled on upper surface, 15–20 cm long, 0.8–0.9 cm wide at ground level; margin exceedingly narrow, cartilaginous, translucent, without teeth or with minute soft whitish teeth; exudate clear. ***Inflorescence*** 0.25–0.30 m high, erect, simple. ***Raceme*** subcapitate, slightly conical, 3.5–4.0 cm long, 3.0–3.5 cm wide, very dense. ***Floral bracts*** 10–13 mm long, 4–6 mm wide. ***Pedicels*** 1 mm long. ***Flowers***: *perianth* yellowish-green, scented, 10–15 mm long, 4 mm across ovary, not narrowed above ovary, slightly narrowed towards slightly upturned mouth, cylindrical-trigonous; outer segments free to base; *stamens* exserted 2–3 mm; *style* exserted 3–5 mm.

#### Flowering time.

January–February.

#### Habitat.

Stony ground in high altitude open grassland in areas characterised by cold winters and high rainfall. Reasonably heavy and sometimes shale soils.

#### Diagnostic characters.

*Aloe modesta* can be distinguished from other grass aloes in KwaZulu-Natal where the leaf bases form a subterranean bulb-like swelling (*Aloe bergeriana*, *Aloe inconspicua* and *Aloe kniphofioides*), by the narrow leaves (15–20 × 0.8–0.9 cm) with minute translucent marginal teeth and that are copiously spotted near the base of the lower surface. It is also characterised by the very dense, unbranched, subcapitate raceme (3.5–4.0 cm long) with almost sessile, yellow, sweetly scented flowers (10–15 mm long). It is the only species of aloe with scented flowers outside of Madagascar (Dyer and Hardy 1974; Van der Riet 1977; Glen and Hardy 2000).

#### Conservation status.

Endangered. Threats include commercial afforestation, overgrazing, alien invasives and urban expansion (Raimondo et al. 2009, L. von Staden pers. comm.).

#### Distribution.

**Figure 25.**
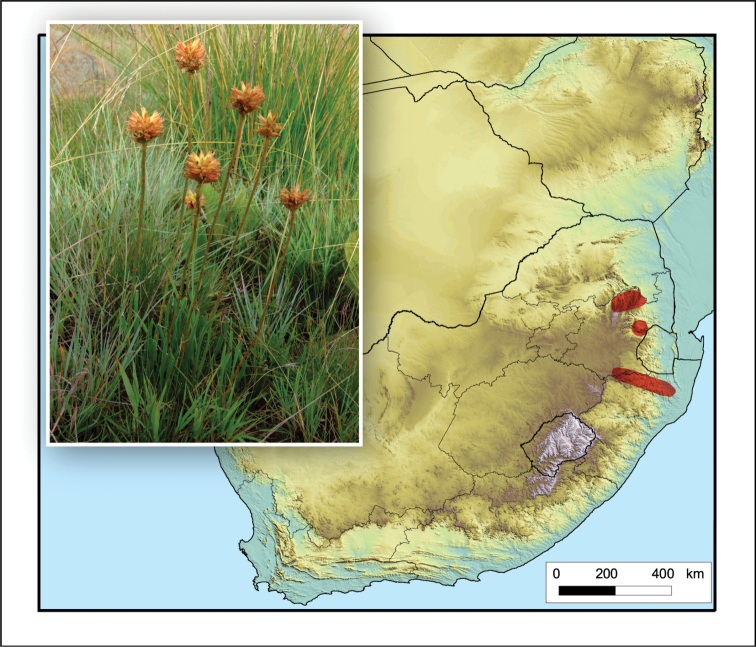
*Aloe modesta*. Photo: H.M. Steyn.

Known only from the mountains around Barberton and near Dullstroom and Lydenburg, Mpumalanga and from the Wakkerstroom area near the KwaZulu-Natal border, South Africa (Fig. 25).

### ^NE^ Aloe mudenensis

Taxon classificationPlantaeAsparagalesAsphodelaceae

Reynolds

D60519E5-7B24-517C-9903-22D68EF4817D

#### Common names.

Muden aloe (English); kleinaalwyn (Afrikaans); icena (Zulu).

#### Description.

Caulescent plants, 0.25–0.5 m tall; rosettes simple or sometimes in small groups. ***Stem*** sometimes absent, usually up to 0.8 m, unbranched, erect or sometimes decumbent, without persistent dried leaves. ***Leaves*** densely rosulate, spreading, bluish-green, paler on lower surface, with numerous irregularly scattered white oblong spots on both surfaces, spots sometimes in irregular transverse bands, sometimes lineate, lower surface sometimes without spots and lineate, ovate-lanceolate, attenuate, 25–30 cm long, 8–9 cm wide; margin horny, with pungent, deltoid, brown, usually straight teeth, up to 7 mm long, 10–20 mm apart; exudate clear, drying reddish-purple. ***Inflorescence*** up to 1 m high, erect, 4- to 8-branched from about middle or below. ***Racemes*** subcapitate, broadly cylindrical, slightly conical, ± 12 cm long, 8–9 cm wide, rather dense. ***Floral bracts*** 12–15 cm, 2–4 mm wide. ***Pedicels*** 20–25 cm long. ***Flowers***: *perianth* salmon-orange, sometimes red, 25–35 mm long, 8 mm across ovary, abruptly constricted above ovary to form subglobose basal swelling, enlarging towards wide-open mouth, slightly decurved; outer segments free for 5–9 mm; *stamens* exserted to 4 mm; *style* exserted to 5 mm.

#### Flowering time.

June–July.

#### Habitat.

Valley bushveld and thicket on sandy loam. Lower areas of warm valleys.

#### Diagnostic characters.

*Aloe mudenensis* can be distinguished from other maculate aloes in KwaZulu-Natal (*Aloe dewetii*, Aloe maculata subsp. maculata, *Aloe parvibracteata*, *Aloe prinslooi*, *Aloe pruinosa*, *Aloe suffulta*, *Aloe umfoloziensis*, *Aloe vanrooyenii* and *Aloe viridiana*) by the short, usually erect stem, without persistent dried leaves. Rosettes are usually solitary or in small groups. Leaves are spreading, 25–30 × 8–9 cm and spotted on both surfaces, with the paler lower surface sometimes without spots and lineate. Marginal teeth are up to 7 mm long. The 4- to 8-branched inflorescence (up to 1 m high) has rather dense, cylindrical, yet terminally rounded racemes (± 12 × 8–9 cm) with spreading buds and flowers. Pedicels are 20–25 cm long. Flowers are salmon-orange, sometimes red, 25–35 mm long and with a subglobose basal swelling (8 mm diameter).

#### Conservation status.

Near-threatened. Threats include silviculture, agriculture (mainly sugarcane) and urban expansion, as well as overgrazing (L. von Staden pers. comm.).

#### Distribution.

**Figure 26.**
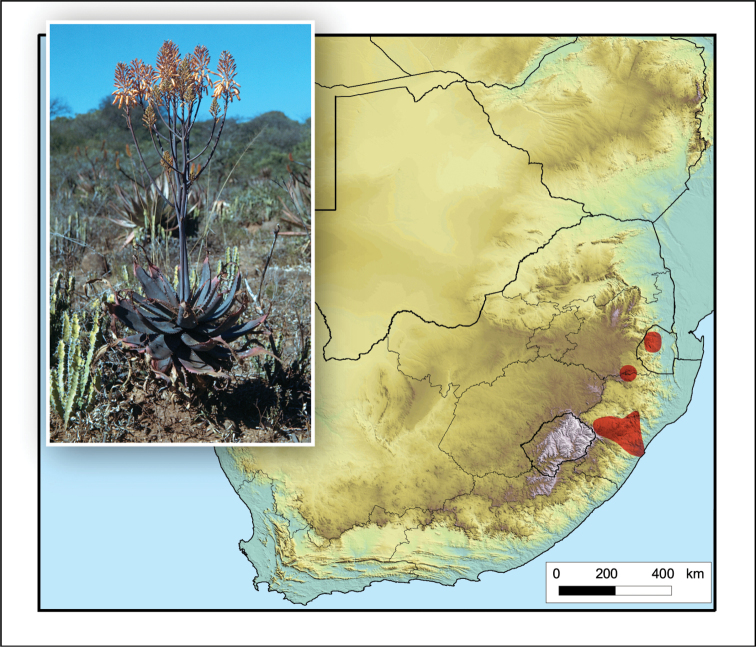
*Aloe mudenensis*. Photo: G.W. Reynolds.

KwaZulu-Natal midlands, on the Mpumalanga border with northern KwaZulu-Natal, South Africa and in Eswatini (Fig. 26).

### Aloe myriacantha

Taxon classificationPlantaeAsparagalesAsphodelaceae

(Haw.) Schult. & Schult.f.

98D5E382-F1ED-5440-ACC7-A6ADB6091305

#### Common names.

umakhuphulwane (Zulu).

#### Description.

Grass aloe, 0.2–0.3 m high. Acaulescent plants; rosettes solitary or sometimes suckering to form small groups. ***Leaves*** rosulate, erectly spreading, dull green, with few white spots towards base, with more copious somewhat tuberculate-subspinulescent spots on lower surface, narrowly linear, keeled, ± 25 cm long, 0.4–1.0 cm wide; margin with minute white teeth, up to 0.5–1 mm long and more crowded near base, smaller towards apex; exudate clear. ***Inflorescence*** 0.20–0.30 m high, erect, simple. ***Raceme*** capitate, 4.5–8.0 cm long, dense. ***Floral bracts*** 10–20 mm long, 5–12 mm wide. ***Pedicels*** 10–25 mm long. ***Flowers***: *perianth* dull white to dull reddish-pink or purple, rarely greenish-white, 15–20 mm long, not or only slightly narrowed above ovary, basally substipitate, narrowing slightly towards mouth, cylindrical-trigonous, mouth distinctly bilabiate and upturned; outer segments free to base; *stamens* and *style* exserted 0–1 mm.

#### Flowering time.

January–May/April (southern Africa), May–June (Kenya and Uganda).

#### Habitat.

Grows amongst rocks and on rocky slopes in high-altitude montane grassland.

#### Diagnostic characters.

*Aloe myriacantha* is distinguished from other grass aloes in KwaZulu-Natal with strongly-keeled leaves (*Aloe cooperi* and *Aloe sharoniae*) by the dull pinkish-red flowers (15–20 mm long) with a distinctly bilabiate upturned mouth. The inflorescence (0.20–0.30 m high) is equal to or slightly longer than the rosulate leaves (± 25 cm long). Leaves have a minutely toothed margin and have spots near the base, with the spots more copious and somewhat tuberculate-subspinulescent on the lower surface.

#### Conservation status.

Least Concern (Raimondo et al. 2009).

#### Distribution.

**Figure 27.**
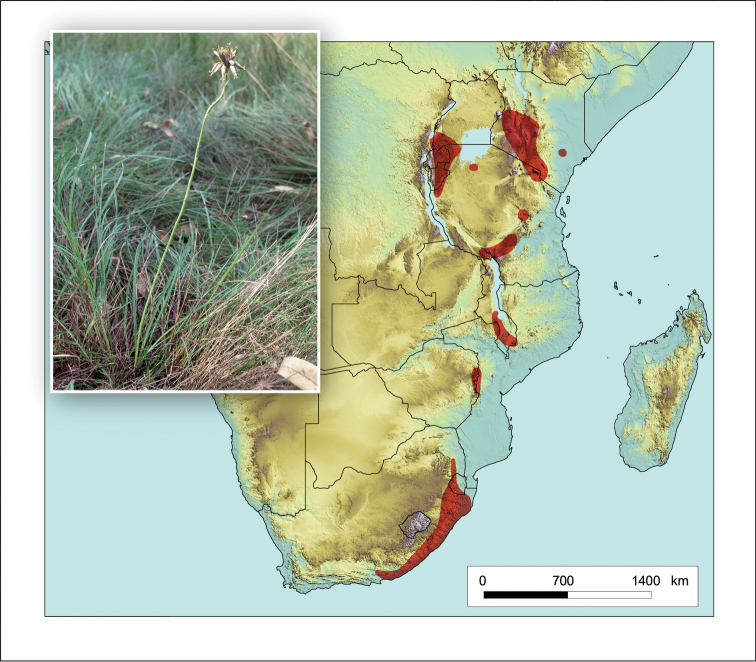
*Aloe myriacantha*. Photo: N.R. Crouch.

A typical Afromontane (sensu White 1983) floristic element, this species has probably the widest distribution range of any *Aloe*. It occurs from the Humansdorp area in the Eastern Cape, along the coast and widespread through KwaZulu-Natal, northwards along the escarpment to the Bosbokrand area in Mpumalanga, South Africa and also in western Eswatini. It is also found further north on the border between Zimbabwe and Mozambique, in Malawi and the Eastern Arc of mountains in Tanzania and Kenya, as well as Uganda, Burundi and Rwanda in southern Tropical Africa (Fig. 27).

### ^E^ Aloe neilcrouchii

Taxon classificationPlantaeAsparagalesAsphodelaceae

Klopper & Gideon F.Sm.

E6F621A1-6253-5410-8323-A2DEDFF20940

#### Common names.

Neil’s aloe (English); neilse-aalwyn (Afrikaans).

#### Description.

Grass aloe. ***Stem*** up to 0.95 m long, decumbent to erect, branched mainly from base, forming robust offshoots along its length, without persistent dried leaves. ***Leaves*** deciduous, densely rosulate, erectly spreading, green, with numerous elongated, white, somewhat tuberculate spots on both surfaces, deltoid to ovate-lanceolate, up to 43 cm long, up to 13.5 cm wide at base; margin narrow, cartilaginous, whitish, with small whitish, deltoid, irregularly spaced teeth, 1–2 mm long, 2–5 mm apart; leaf exudate clear, drying clear, not bitter. ***Inflorescence*** 0.6–0.8 m high, erect, simple. ***Raceme*** capitate, ± 12 cm long, 10 cm wide, dense. ***Floral bracts*** ± 30 mm long, 7 mm wide. ***Pedicels*** 30–45 mm long. ***Flowers***: *perianth* salmon-pink, green-tipped, ± 45 mm long, 10–13 mm across ovary, slightly narrowed above ovary, slightly constricted just before flared mouth, cylindrical-trigonous; outer segments free almost to base; *stamens* not or only slightly exserted; *style* exserted to ± 5 mm.

#### Flowering time.

December–January.

#### Habitat.

Southeast-facing aspects in rocky grassland.

#### Diagnostic characters.

*Aloe neilcrouchii* can be distinguished from other grass aloes in KwaZulu-Natal with unkeeled leaves that are wider than 3.5 cm (*Aloe boylei*, *Aloe ecklonis*, *Aloe hlangapies* and *Aloe kraussii*), by the large rosettes of erectly spreading, rosulate leaves (up to 43 × 13.5 cm), with copious white tuberculate spots on both surfaces. It is further characterised by the long, sprawling, leafless stems of up to almost 1 m long, which branch at the base and form offshoots along its length. The unbranched inflorescences (0.6–0.8 m high) have dense, capitate racemes (± 12 cm long) with large (± 45 mm long), salmon-pink, rather straight flowers. This is the largest and most robust species of the leptoaloe group, also known as ‘slender aloes’ (Klopper and Smith 2010; Smith et al. 2011).

#### Conservation status.

Endangered. Threats include habitat fragmentation and destruction owing to commercial silvicultural and agricultural practices (Johnson et al. 2011).

#### Distribution.

**Figure 28.**
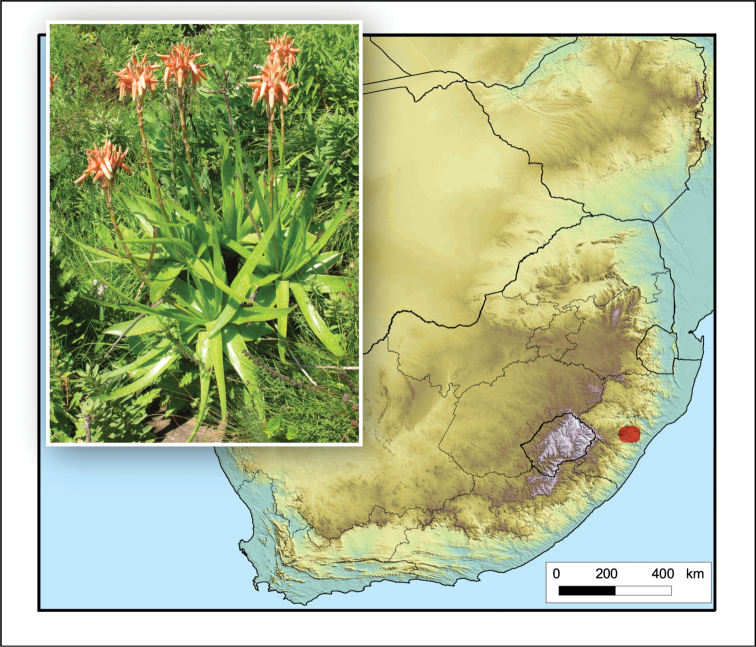
*Aloe neilcrouchii*. Photo: N.R. Crouch.

Known from only two localities near Karkloof and New Hanover, in the midlands of KwaZulu-Natal, South Africa (Fig. 28).

### ^E^ Aloe nicholsii

Taxon classificationPlantaeAsparagalesAsphodelaceae

Gideon F.Sm. & N.R.Crouch

C3DCCF37-5FE5-51D0-B399-FFC37A374542

#### Common names.

Shiny aloe (English); blinkaalwyn (Afrikaans).

#### Description.

Grass aloe of ± 0.30–0.36 m tall. ***Stem*** short, ± 0.06–0.14 m high, erect, sometimes unbranched, usually suckering to form clumps; without persistent dried leaves. ***Leaves*** distichous becoming semi-rosulate, flaccidly spreading, mid-green to light yellowish-green, occasionally with a few scattered white spots towards base, spots more common on lower surface, texture smooth, narrowly linear, tapering towards apex, canaliculate, 20–46 cm long, 2.0–3.5 cm wide; margin coarse, faintly ivory-coloured, mostly without teeth or sometimes with tiny, harmless, triangular, ivory-coloured to greenish-white teeth, less than 0.5 mm long, 5–10 mm apart; exudate drying translucent. ***Inflorescence*** 0.30–0.46 m tall, erect, simple. ***Raceme*** capitate, 3.0–3.5 cm long, 5–6 cm wide, dense. ***Floral bracts*** 10–26 mm long. ***Pedicels*** 25–30 mm long. ***Flowers***: *perianth* metallic salmon-pink above, greenish below, purplish-brown tipped, lightly pruinose, 13–16 mm long, 5 mm across middle, enlarging towards slightly open, distinctly upturned mouth, tubular cymbiform; outer segments free for most of their length; *stamens* not exserted; *style* exserted.

#### Flowering time.

January–March.

#### Habitat.

Open rocky grassland.

#### Diagnostic characters.

*Aloe nicholsii* can be distinguished from other grass aloes in KwaZulu-Natal with unkeeled leaves that are usually narrower than 3.5 cm and that lack a bulb-like underground swelling (*Aloe dominella*, *Aloe linearifolia*, *Aloe micracantha*, *Aloe minima*, *Aloe parviflora* and *Aloe saundersiae*), by the unbranched inflorescences (0.30–0.46 m high) with dense, capitate racemes and pedicels 25–30 mm long. The small flowers (13–16 mm long) are pruinose, greenish below and a metallic salmon-pink above, with a distinctly upturned mouth. The distichous to semi-rosulate leaves (20–46 × 2.0–3.5 cm) are flaccidly spreading and occasionally with a few scattered white spots towards the base, more commonly on the lower surface. Rosettes are usually in dense groups (Crouch et al. 2011).

#### Conservation status.

Critically Endangered. Threats include habitat degradation due to overgrazing, urban expansion and commercial afforestation (Von Staden 2013).

#### Distribution.

**Figure 29.**
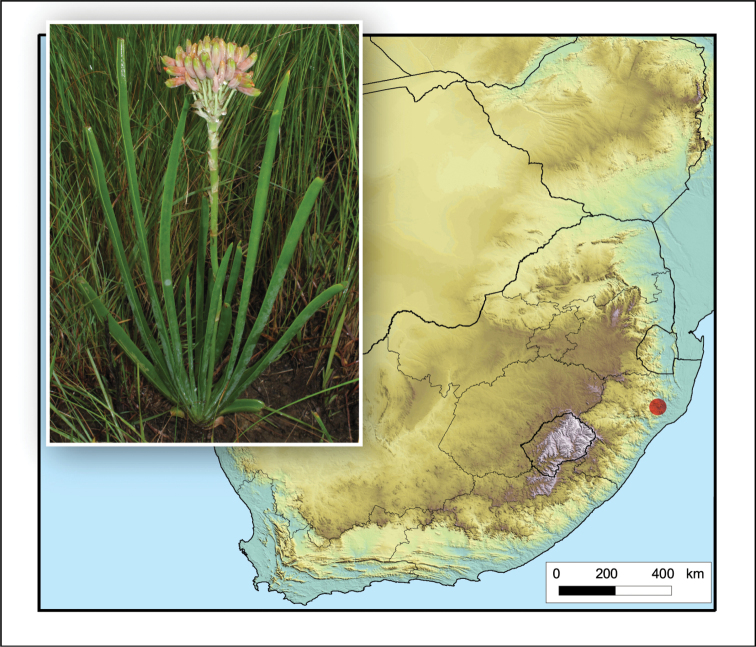
*Aloe nicholsii*. Photo: N.R. Crouch.

Known from a small area near Babanango in the KwaZulu-Natal midlands, South Africa (Fig. 29).

### Aloe parvibracteata

Taxon classificationPlantaeAsparagalesAsphodelaceae

Schönland nom. cons.

BFE90506-9BC1-5C07-9F75-1726A143FB71

#### Common names.

Lowveld spotted aloe (English); pers-bontaalwyn (Afrikaans); icena, inkalane (Zulu).

#### Description.

Acaulescent plants or ***stem*** very short, up to 0.4 m tall; rosettes suckering to form large dense groups. ***Leaves*** densely rosulate, spreading-decurved, upper surface green to brownish-green to purplish-green, with numerous oblong dull white spots, usually arranged in interrupted, undulating transverse bands, lower surface paler green, usually without spots, narrowly lanceolate, gradually attenuate, 30–40 cm long, 6–10 cm wide at base; margin with pungent brown teeth, sometimes remarkably deflexed, 3–5 mm long, 10–15 mm apart; exudate honey-coloured, drying deep purple. ***Inflorescences*** 1.0–1.5 m high, erect, 4- to 8-branched from above middle. ***Racemes*** cylindrical, slightly acuminate, terminal 15–30 cm long, 6–7 cm wide, lateral shorter, lax. ***Floral bracts*** 8–12 mm long. ***Pedicels*** 6–15 mm long. ***Flowers***: *perianth* dull to somewhat glossy red or orange, 30–40 mm long, 7–9 mm across ovary, abruptly constricted above ovary to form globose basal swelling, enlarging towards sometimes wide-open mouth, slightly decurved; outer segments free for 8–10 mm; *stamens* and *style* exserted 1–2 mm.

#### Flowering time.

June–July.

#### Habitat.

On rocky outcrops in flat grassland in hot, low-lying thorny savannah and similar thorny woodland in the Lebombo Mountains.

#### Diagnostic characters.

*Aloe parvibracteata* can be distinguished from other maculate aloes in KwaZulu-Natal (*Aloe dewetii*, Aloe maculata subsp. maculata, *Aloe mudenensis*, *Aloe prinslooi*, *Aloe pruinosa*, *Aloe suffulta*, *Aloe umfoloziensis*, *Aloe vanrooyenii* and *Aloe viridiana*) by the rosettes that sucker profusely to form large groups. It is further characterised by the spreading-decurved almost depressed leaves (30–40 × 6–10 cm) that give the rosette a ‘flattened out’ appearance. Leaves are spotted on the upper surface, while the paler lower surface is usually without spots and marginal teeth are 3–5 mm long. The 4- to 8-branched inflorescence (1.0–1.5 m high) has a very slender (but rigidly erect) peduncle and branches. Racemes are lax, cylindrical-acuminate and 15–30 cm long, with pedicels 6–15 mm long. Flowers are dull to somewhat glossy red or orange, 30–40 mm long and with a globose basal swelling (7–9 mm diameter).

#### Conservation status.

Least Concern (Raimondo et al. 2009).

#### Distribution.

**Figure 30.**
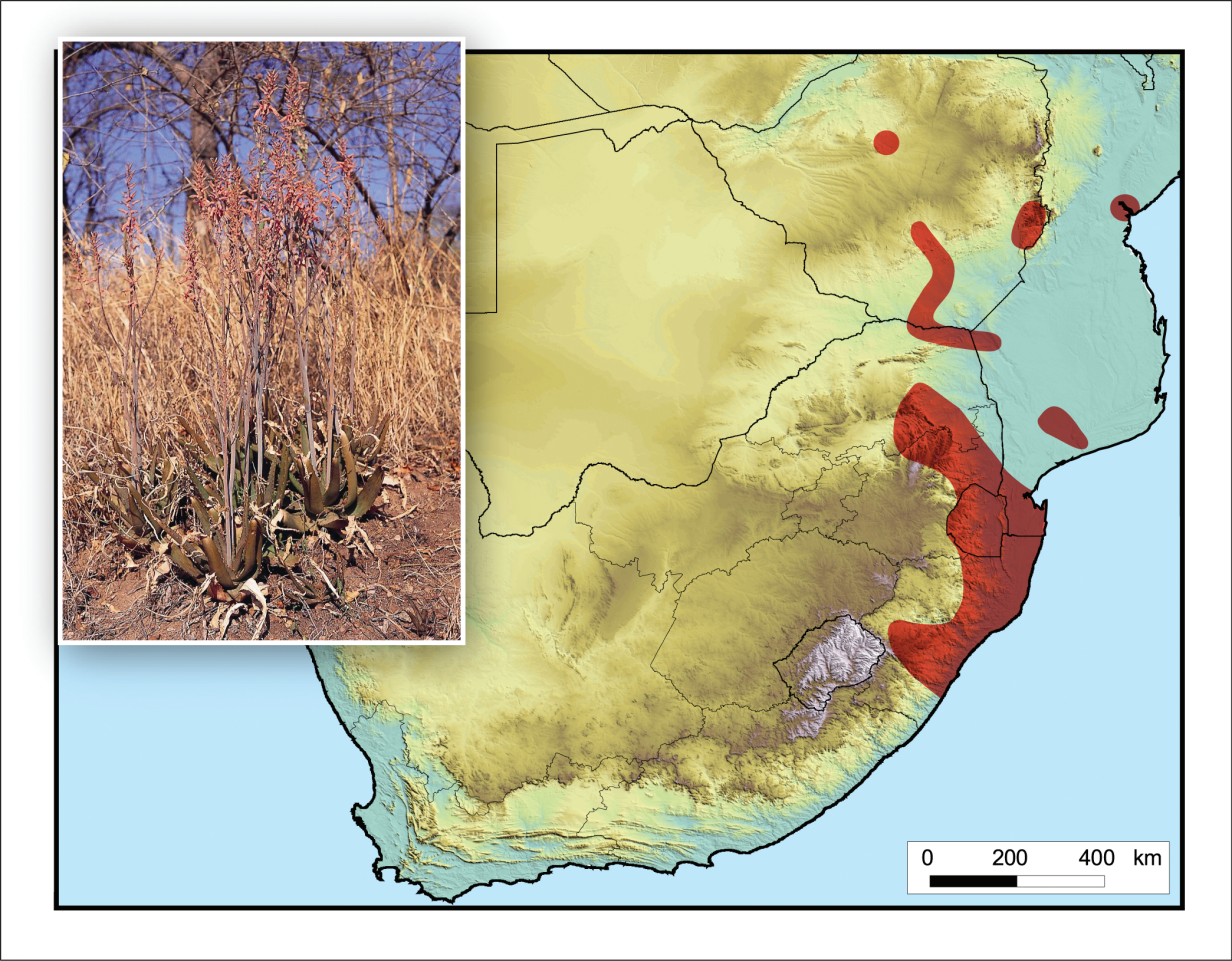
*Aloe parvibracteata*. Photo: G.F. Smith.

Northern KwaZulu-Natal, eastern Mpumalanga and Limpopo in South Africa, also in Eswatini, southern Mozambique and Zimbabwe (Fig. 30).

#### Notes.

Until recently, *Aloe monteiroae* Baker was regarded as an insufficiently known species, since its true identity could not be determined with certainty (see Reynolds 1950 and Carter 2001). The discovery of a population of aloes near Komatipoort, Mpumalanga, that match the description of *A. monteiroae* has enabled Crouch et al. (2015) to confirm that it is conspecific with *A. parvibracteata*. However, *A. monteiroae* is the older of the two names. To avoid nomenclatural disruptions by allowing a previously unknown name to replace one that has for long been in common use for a widespread aloe, a successful proposal was published to conserve the familiar name *Aloe parvibracteata* and enable its continued use for this aloe (Klopper et al. 2015; Wilson 2017).

### ^E^ Aloe parviflora

Taxon classificationPlantaeAsparagalesAsphodelaceae

Baker

C6B454A0-CC1B-5980-8C75-FDAF331C2E5B

#### Description.

Grass aloe. Acaulescent, rosettes solitary, erect. ***Leaves*** few, distichous to rosulate, spreading, deciduous, bright green, lower surface with nerves and numerous spinulescent white spots especially towards base, texture tuberculate-muricate, lorate-linear, attenuate at base, apex obtuse, 20–25 cm long, 0.6–0.8 cm wide; margin ciliate with minute white crowded teeth; exudate clear. ***Inflorescence*** 0.4 m high, erect, simple. ***Raceme*** capitate, ± 3 cm long, 3 cm wide, dense. ***Floral bracts*** 8–12 mm long. ***Pedicels*** 8–12 mm long. ***Flowers***: *perianth* pale rose, 8 mm long, widening slightly towards middle, narrowing towards mouth, straight or slightly decurved, shortly cylindrical; outer segments free to base; *stamens* and *style* not exserted.

#### Flowering time.

January–March.

#### Habitat.

Short grassland, on level or gently sloping areas on the summit of hills, in shallow soil over exposed sloping sandstone rock sheets and in rocky places with thin soil and sparse grass.

#### Diagnostic characters.

*Aloe parviflora* can be distinguished from other grass aloes in KwaZulu-Natal with unkeeled leaves that are usually narrower than 3.5 cm and that lack a bulb-like underground swelling (*Aloe dominella*, *Aloe linearifolia*, *Aloe micracantha*, *Aloe minima*, *Aloe nicholsii* and *Aloe saundersiae*), by the distichous to rosulate, spreading, lorate-linear leaves (20–25 × 0.6–0.8 cm) that are distinctly muricate with soft spinulescent white spots on the lower surface. It is further characterised by the peduncle, which has numerous small spines on the lower part. The unbranched inflorescences (0.4 m high) have dense, capitate racemes with pedicels 8–12 mm long and very small, pale rose flowers (8 mm long). Rosettes are solitary.

#### Conservation status.

Vulnerable. Threats include habitat loss and degradation owing to urban expansion, as well as alien invasives, overgrazing and incorrect fire management (L. von Staden pers. comm.).

#### Distribution.

**Figure 31.**
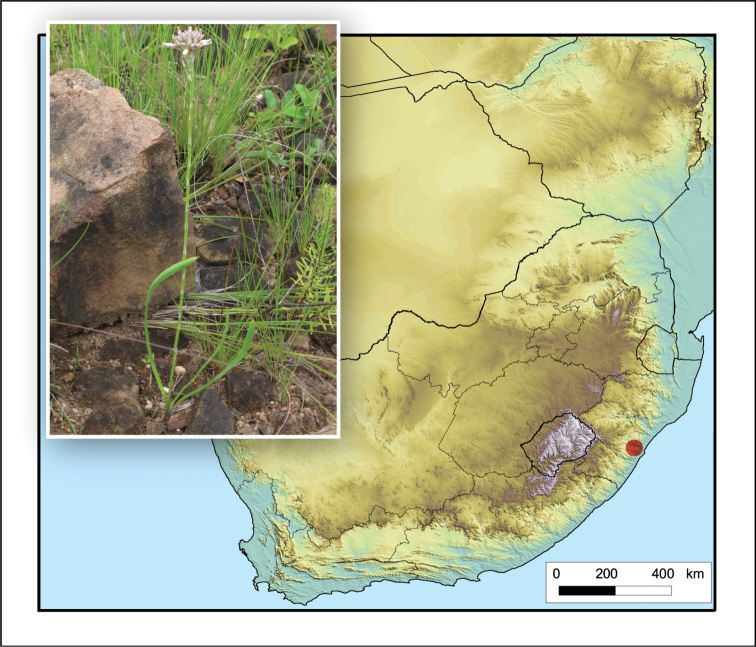
*Aloe parviflora*. Photo: N.R. Crouch.

Confined to a small area between Pinetown and Cato Ridge in central KwaZulu-Natal, South Africa (Fig. 31).

#### Notes.

*Aloe parviflora* is sometimes considered to be conspecific with *Aloe minima* Baker (Glen and Hardy 2000).

### Aloe pluridens

Taxon classificationPlantaeAsparagalesAsphodelaceae

Haw.

C6A95298-3DE4-5C33-BD01-B26357113CAC

#### Common names.

French aloe, many-toothed tree-aloe (English); fransaalwyn (Afrikaans).

#### Description.

Tree or shrub, up to 3 m high. ***Stem*** ± 2–3 m high, can reach up to 5 m, simple or branched at ground level or from middle or above, erect, with persistent dried leaves in upper half. ***Leaves*** densely rosulate, erectly spreading and gracefully recurved, sometimes falcately deflexed, pale to yellowish-green, obscurely lineate, lanceolate-falcate, 60–70 cm long, 5–6 cm wide at base; margin narrow, white, cartilaginous, with deltoid, incurved, white or very pale pink teeth, 2–3 mm long, 5–10 mm apart; exudate clear. ***Inflorescence*** 0.8–1.0 m high, erect, up to 4-branched from below middle. ***Racemes*** conical, 25–30 cm long, dense. ***Floral bracts*** ± 20 mm long, 10–12 mm wide. ***Pedicels*** 30–35 mm long. ***Flowers***: *perianth* salmon pink to orange to dull scarlet or yellow, 40–45 mm long, 6–7 mm across ovary, slightly constricted above ovary, slightly widening towards mouth, cylindrical-trigonous; outer segments free to base; *stamens* exserted 2–4 mm; *style* exserted to 5 mm.

#### Flowering time.

May–June.

#### Habitat.

Succulent thicket vegetation on hillside slopes within a coastal strip and, in the north of its range, along the ecotone of coastal forest pockets.

#### Diagnostic characters.

*Aloe pluridens* differs from the other tall often single-stemmed aloes in KwaZulu-Natal (*Aloe candelabrum*, *Aloe marlothii*, *Aloe rupestris*, *Aloe spectabilis* and *Aloe thraskii*) with branched inflorescences, by having narrow (60–70 × 5–6 cm), erectly spreading and gracefully recurved, pale green to yellowish-green, obscurely lineate leaves with small crowded pinkish-white marginal teeth and exudate with a distinct sharp odour. The inflorescence is up to 4-branched with erect, rather lax, conical racemes of 25–30 cm long. Flowers are salmon-pink to orange to dull scarlet or yellow and 40–45 mm long. Note though that the flowers of *A. pluridens* never take on the bright scarlet colour of some forms of *A. arborescens*.

#### Conservation status.

Least Concern (Raimondo et al. 2009).

#### Distribution.

**Figure 32.**
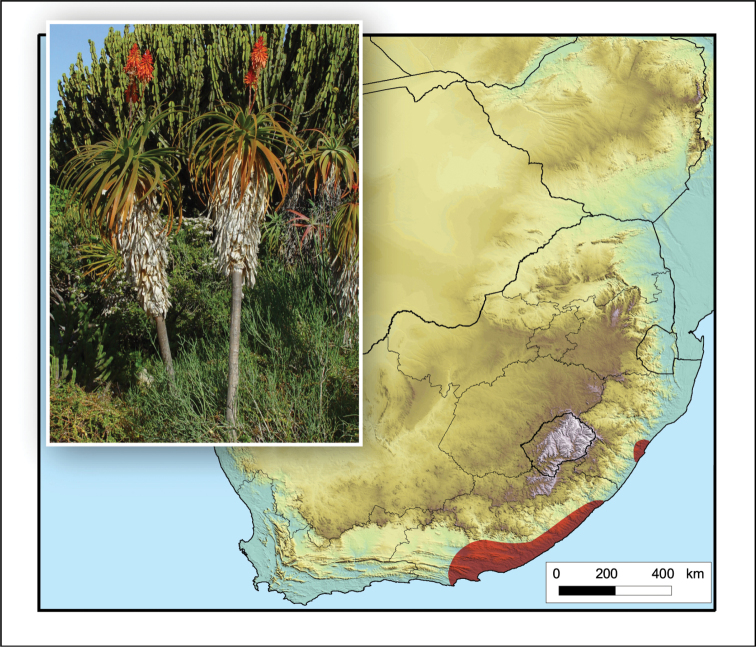
*Aloe pluridens*. Photo: G. Nichols.

This species has a disjunct distribution range. It occurs from the Humansdorp area to the Kei River Mouth in the Eastern Cape, as well as in the Durban area in KwaZulu-Natal, South Africa (Fig. 32; Walker et al. 2019a).

### Aloe pratensis

Taxon classificationPlantaeAsparagalesAsphodelaceae

Baker

C10EDB08-5625-549C-9D8D-E1316DDB92AE

#### Common names.

Meadow aloe (English); bergaalwyn (Afrikaans).

#### Description.

Acaulescent plants; rosettes solitary or branching from the base to form small groups, 15–25 cm diameter. ***Leaves*** densely rosulate, erectly spreading to slightly incurved, glaucous, lineate, deltoid-lanceolate, 10–17 cm long, 4–6 cm wide, lower surface with few scattered red-brown spines from white tuberculate bases, keel armed with few brown spines, 2–3 mm long; margin not distinctly coloured, with pungent, deltoid, reddish-brown teeth, ± 5 mm long, ± 10 mm apart; exudate clear, drying deep orange. ***Inflorescence*** 0.5–0.6 m high, erect, simple; peduncle almost entirely covered with large, thin, imbricate sterile bracts. ***Racemes*** cylindrical, ± 20 cm long, dense; buds completely hidden by large bracts. ***Floral bracts*** up to 40 mm long, 15–18 mm wide. ***Pedicels*** 25–40 mm long. ***Flowers***: *perianth* rose-red, 35–40 mm long, ± 5 mm across ovary, slightly enlarged towards mouth, basally stipitate, cylindrical; outer segments free to base; *stamens* exserted 0–1 mm; *style* exserted 1–2 mm.

#### Flowering time.

August–December.

#### Habitat.

In exposed positions amongst rocks in sloping montane grassland in some of the coldest parts of the southern Drakensberg.

#### Diagnostic characters.

*Aloe pratensis* can easily be distinguished from other KwaZulu-Natal aloes by being an acaulescent plant with smallish rosettes (± 20 cm diameter) that occurs solitary or in small groups. Leaves (10–17 × 4–6 cm) have pungent reddish-brown marginal teeth (± 5 mm long) and spines on the lower surface that arise from white tuberculate bases. The inflorescence (0.5–0.6 m high) is simple with the peduncle covered in large, imbricate bracts. Racemes are cylindrical and dense and elongates significantly as flowering progresses, although the length of the peduncle stays roughly constant. Flower buds are hidden by large floral bracts. Flowers are cylindrical and rose-red (35–40 mm long).

#### Conservation status.

Least Concern (Raimondo et al. 2009).

#### Distribution.

**Figure 33.**
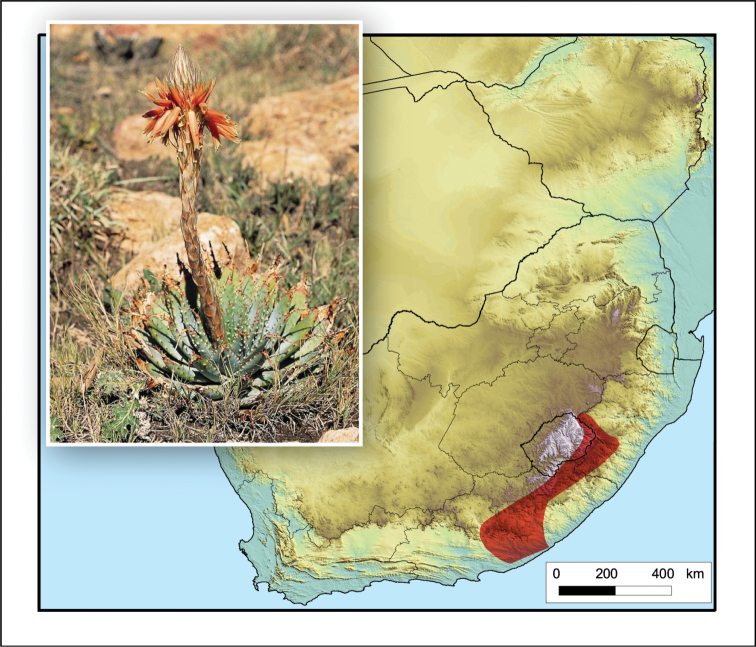
*Aloe pratensis*. Photo: G.F. Smith.

It occurs in the central and northern Eastern Cape and along the Great Escarpment and in south-western KwaZulu-Natal along the Drakensberg Mountain Range to Royal Natal National Park, South Africa, as well as Lesotho (Fig. 33).

### ^E^ Aloe prinslooi

Taxon classificationPlantaeAsparagalesAsphodelaceae

I.Verd. & D.S.Hardy

2E1F5BFC-8AFE-5CC2-8CE8-F0B82DB8AA49

#### Common names.

Spotted aloe (English); bontaalwyn (Afrikaans).

#### Description.

Acaulescent plants, 0.15–0.25 m high; rosettes usually solitary, erect. ***Leaves*** rosulate, suberect to spreading, light green, with white, oblong spots, denser on upper surface, occasionally arranged in transverse bands, shortly deltoid, 14–20 cm long, 4–8 cm wide; margin with pungent brown teeth, ± 4 mm long, 5–7 mm apart; exudate clear. ***Inflorescence*** up to 0.6 m high, erect, 2- to 5-branched above middle. ***Racemes*** corymbose-capitate, 6–12 cm long, 6–7 cm wide, dense. ***Floral bracts*** 15–30 mm long, 3–5 mm wide. ***Pedicels*** 12–30 mm long. ***Flowers***: *perianth* pale whitish-green, tinged with pale to deep pink, 13–17 mm long, sometimes slightly narrowing above ovary, widening towards slightly upturned mouth, cylindrical; outer segments free for 5–7 mm; *stamens* exserted 0–1 mm; *style* slightly or not exserted.

#### Flowering time.

June–October.

#### Habitat.

Dense grass understorey of open woodland in KwaZulu-Natal midlands on thin soil. More rarely in open, rocky outcrops. Rainfall relatively low, summers hot and winters very cold.

#### Diagnostic characters.

*Aloe prinslooi* can be distinguished from other maculate aloes in KwaZulu-Natal (*Aloe dewetii*, Aloe maculata subsp. maculata, *Aloe mudenensis*, *Aloe parvibracteata*, *Aloe pruinosa*, *Aloe suffulta*, *Aloe umfoloziensis*, *Aloe vanrooyenii* and *Aloe viridiana*) by its short, 2- to 5-branched inflorescence (up to 0.6 m high) with almost spherical, very dense racemes (6–12 × 6–7 cm) of creamish to pinkish-white flowers (up to 17 mm long) that lack the distinctive globose basal swelling typical of the maculate aloes. Pedicels are 12–30 mm long. Leaves are suberect to spreading, 14–20 × 4–8 cm and spotted on both surfaces, with the spots being denser on the upper surface. Marginal teeth are ± 4 mm long.

#### Conservation status.

Endangered. Threats include trampling by livestock and too frequent fires. In the past, populations were negatively impacted by illegal collecting (Raimondo et al. 2009, L. von Staden pers. comm.).

#### Distribution.

**Figure 34.**
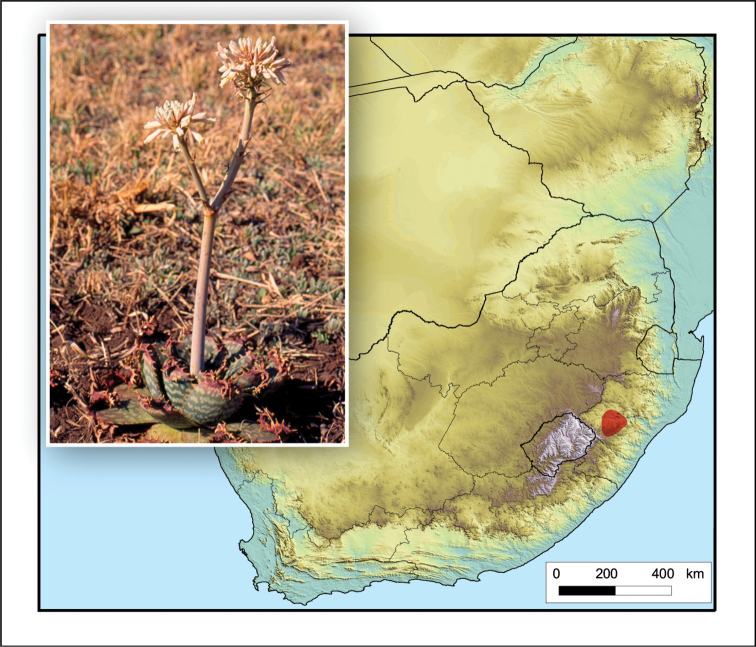
*Aloe prinslooi*. Photo: P. Joffe.

Limited to an area near Colenso in the KwaZulu-Natal midlands, South Africa (Fig. 34).

### ^E^ Aloe pruinosa

Taxon classificationPlantaeAsparagalesAsphodelaceae

Reynolds

96470829-8828-54F1-99F5-1CB93268D12F

#### Common names.

Kleinaalwyn, slangkop (Afrikaans); icena elihkulu (Zulu).

#### Description.

Shortly caulescent plant, 0.25–0.60 m tall. ***Stem*** up to 0.5 m, unbranched, procumbent; rosettes solitary, erect. ***Leaves*** densely rosulate, erectly spreading to slightly recurved, bright green, with numerous white, somewhat H-shaped spots, scattered or confluent in wavy, irregular, interrupted, transverse bands, spots more numerous and in more defined transverse bands on lower surface, lanceolate-attenuate, 50–70 cm long, 8–10 cm wide at base; margin with deltoid, pungent, pale pinkish-brown teeth, 3–4 mm long, 15–20 mm apart; exudate honey-coloured, drying deep purple. ***Inflorescence*** 1.4–2.0 m high, erect, ± 11-branched above middle. ***Racemes*** cylindrical-acuminate, terminal up to 30 cm long, 7 cm wide, lateral shorter, usually 10–12 cm long, lax. ***Floral bracts*** 10–20 mm long. ***Pedicels*** 10–20 mm long. ***Flowers***: *perianth* dull dark brownish-red to pinkish white, with heavy grey powdery bloom, 30–40 mm long, 8 mm across ovary, abruptly constricted above ovary to form globose basal swelling, widening towards mouth, sharply decurved, laterally compressed; outer segments free for 5–7 mm; *stamens* exserted 1–2 mm; *style* exserted 1–4 mm.

#### Flowering time.

February–March.

#### Habitat.

In shade in acacia savannah in KwaZulu-Natal midlands on heavy loam in areas of fairly high summer rainfall.

#### Diagnostic characters.

*Aloe pruinosa* can be distinguished from other maculate aloes in KwaZulu-Natal (*Aloe dewetii*, Aloe maculata subsp. maculata, *Aloe mudenensis*, *Aloe parvibracteata*, *Aloe prinslooi*, *Aloe suffulta*, *Aloe umfoloziensis*, *Aloe vanrooyenii* and *Aloe viridiana*) by the tall, ± 11-branched inflorescence (1.4–2.0 m high) with the peduncle and flowers that are very heavily coated with a greyish powdery substance. The flowers, which are dull dark brownish-red to pinkish-white, 30–40 mm long and with a globose basal swelling (8 mm diameter), have the most pronounced powdery-covered leaves and inflorescence of all South African aloe species. It is further characterised by the erectly spreading to slightly recurved leaves (50–70 × 8–10 cm) that are spotted on both surfaces, with the spots more pronounced on the lower surface. Marginal teeth are 3–4 mm long. The lax racemes are cylindrical-acuminate, with the terminal one the longest (up to 30 × 7 cm) and the lateral ones usually 10–12 cm long (Smith et al. 1999).

#### Conservation status.

Endangered. Threats include urban expansion and harvesting for use in traditional medicine (Raimondo et al. 2009, L. von Staden pers. comm.).

#### Distribution.

**Figure 35.**
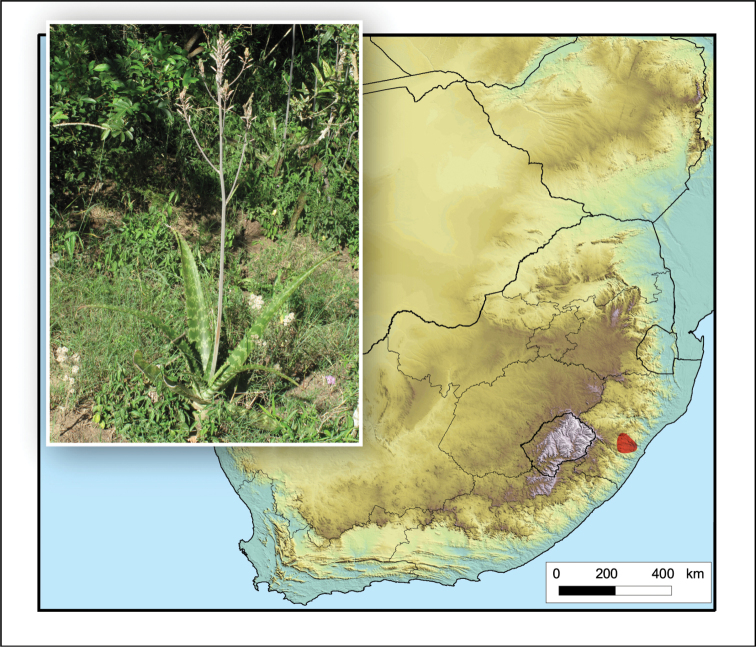
*Aloe pruinosa*. Photo: N.R. Crouch.

Occurs from Pietermartizburg to Durban and northwards to the uThukela (Tugela) River valley, KwaZulu-Natal, South Africa (Fig. 35).

### ^E^ Aloe reitzii Reynolds var. vernalis

Taxon classificationPlantaeAsparagalesAsphodelaceae

D.S.Hardy

B91EB2BF-134E-5153-A53D-2FEB8505FB68

#### Common names.

Reitz’s spring aloe (English); lente-bergaalwyn (Afrikaans).

#### Description.

Acaulescent plants or rarely with short ***stem*** of up to 0.5 m, simple, rarely branched, procumbent; rosette solitary, erect. ***Leaves*** densely rosulate, arcuate-erect, dull green, without spots, texture smooth, lanceolate-ensiform, 40–65 cm long, 5–9 cm wide, lower surface sometimes with brownish spines in median line near apex, leaf tip armed with pungent spine; margin not distinctly coloured, with deltoid, pungent, brownish to reddish-brown teeth from distinct white base, ± 3 mm long, ± 5 mm apart; exudate drying bright yellow. ***Inflorescence*** 0.70–0.75 m high, erect, 2- to 4-branched from below middle. ***Racemes*** cylindrical, slightly acuminate, 30–40 cm long, 5–6 cm wide, very dense, with open flowers pendent and pressed against the peduncle. ***Floral bracts*** ± 6 mm long, 4–5 mm wide. ***Pedicels*** 3 mm long. ***Flowers***: *perianth* dull orange-red in bud, turning yellow when flowers open, 32–40 mm long, ± 5 mm across ovary, enlarging towards middle, slightly narrowing towards mouth, curved-cylindrical; outer segments free for 15 mm; *stamens* exserted to 8 mm; *style* exserted to 10 mm.

#### Flowering time.

August–September.

#### Habitat.

Steep well-drained granitic slopes in grassland.

#### Diagnostic characters.

Aloe reitzii var. vernalis can be distinguished from other virtually acaulescent, non-maculate aloes in KwaZulu-Natal (*Aristaloe aristata*, Aloe chabaudii var. chabaudii, *Aloe gerstneri*, *Aloe pratensis*, *Aloe suprafoliata* and *Aloe vanbalenii*) by the very dense racemes (30–40 × 5–6 cm) with short erect pedicels (3 mm). Flowers are bicoloured (outer part of tube orange-red, inner part yellow), 32–40 mm long, tubular and curved, pointing downwards and pressed against the peduncle, with rather long-exserted stamens and style. Leaves are narrow (40–65 × 5–9 cm), arcuate-erect, dull green, sometimes with spines on median line of lower surface and with pungent marginal teeth from a distinct white base. Flowering time is in spring.

#### Conservation status.

Vulnerable. Threats include harvesting for medicinal purposes and damage by feeding baboons (Raimondo et al. 2009, L. von Staden pers. comm.).

#### Distribution.

**Figure 36.**
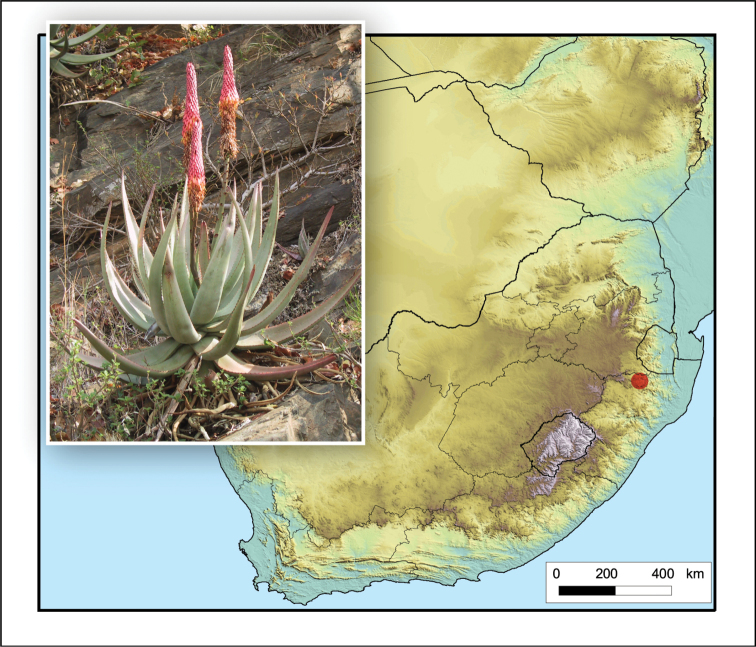
Aloe reitzii var. vernalis. Photo: E. van Wyk.

Confined to a small area in the Vryheid District, KwaZulu-Natal, South Africa (Fig. 36).

#### Notes.

The typical variety, A. reitzii var. reitzii only occurs in the Belfast District in Mpumalanga, South Africa.

### ^NE^ Aloe rupestris

Taxon classificationPlantaeAsparagalesAsphodelaceae

Baker

C5F802AF-590C-54C3-BD25-2A1F28C36B62

#### Common names.

Bottle-brush aloe (English); borselaalwyn, kraalaalwyn (Afrikaans); inkhalane, umhlabanhlazi, uphondonde (Zulu).

#### Description.

Solitary, arborescent plant. ***Stem*** usually unbranched, up to 6–8 m high, erect, with persistent dried leaves in upper third only. ***Leaves*** densely rosulate, erectly spreading to recurved, dull to slightly glossy deep green, without spots, texture smooth, lanceolate-attenuate, 30–70 cm long, 7–10 cm wide; margin deep pink to pale red, with stout, pungent, reddish-brown, deltoid teeth, 4–6 mm long, 8–12 mm apart; exudate honey-coloured. ***Inflorescence*** 1.0–1.3 m high, erect, 6- to 9-branched from above middle, lower branches rebranched. ***Racemes*** cylindrical, very slightly acuminate, somewhat truncate, 20–25 cm long, 7 cm wide, very dense. ***Floral bracts*** ± 1 mm long, 2 mm wide. ***Pedicels*** 1–2 mm long. ***Flowers***: *perianth* orange-yellow in bud, green striped in upper half, lemon-yellow in lower third and orange-yellow to brownish-yellow upwards when mature, 15–20 mm long, 4 mm across ovary, widening slightly towards middle, narrowing at mouth, cylindrical, slightly ventricose; outer segments free for 12 mm; *stamens* exserted 7–15 mm; *style* exserted 7–20 mm.

#### Flowering time.

August–September.

#### Habitat.

Zululand thornveld, coastal plain on sandy soils, sometimes dense bush, usually on rocky outcrops. Areas with warm, completely frost-free winters. Usually found in groups amongst trees.

#### Diagnostic characters.

*Aloe rupestris* differs from the other tall often single-stemmed aloes in KwaZulu-Natal (*Aloe candelabrum*, *Aloe marlothii*, *Aloe pluridens*, *Aloe spectabilis* and *Aloe thraskii*) with branched inflorescences, by having wide (30–70 × 7–10 cm), erectly spreading to recurved leaves that lack surface prickles and have pungent, reddish-brown marginal teeth. The inflorescence is 6- to 9-branched and rebranched with up to 20 erect, very dense, cylindrical, very slightly acuminate and somewhat truncate racemes of 20–25 cm long. Flowers are almost sessile, lemon-yellow to brownish-yellow and 15–20 mm long. The long-exserted deep orange to dark red stamens and style emerge from the flowers straight (not at an angle as in *Aloe thraskii*).

#### Conservation status.

Least Concern (Raimondo et al. 2009).

#### Distribution.

**Figure 37.**
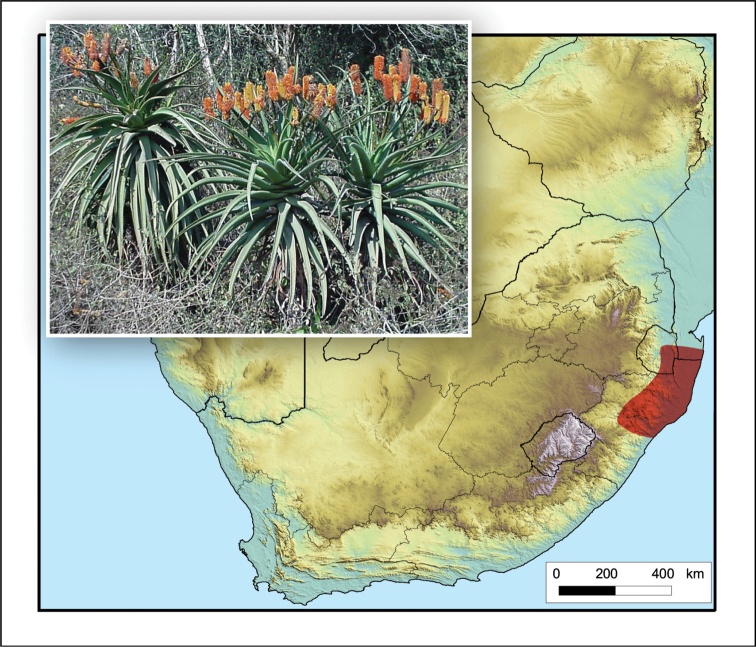
*Aloe rupestris*. Photo: G. Nichols.

Central to northern KwaZulu-Natal in South Africa, eastern Eswatini and southern Mozambique (Fig. 37).

### ^E^ Aloe saundersiae

Taxon classificationPlantaeAsparagalesAsphodelaceae

(Reynolds) Reynolds

925643B8-D0D8-527E-8CBA-4660781A6819

#### Description.

Acaulescent grass aloe, 0.05–0.075 m high; rosettes solitary or suckering to form small tufted groups. ***Leaves*** rosulate, widely spreading to recurved, deciduous, green, without spots or with few white spots near base on lower surface, narrowly linear, 4–10 cm long, 0.3 cm wide, basally amplexicaul and 1 cm wide below ground; margin with rather soft, white, deltoid teeth, ± 0.5 mm long, 1 mm apart; exudate clear. ***Inflorescence*** 0.14–0.18 m high, erect, simple. ***Raceme*** capitate, 2.0–2.5 cm long, 3.0–3.5 mm wide, dense. ***Floral bracts*** 7 mm long, 3–4 mm wide. ***Pedicels*** 8–10 mm long. ***Flowers***: *perianth* pale cream-pink, 9–12 mm long, 3–4 mm across ovary, narrowing towards slightly upturned mouth, cylindrical-trigonous, slightly ventricose; outer segments free to base; *stamens* and *style* not exserted.

#### Flowering time.

February–March.

#### Habitat.

Rocky outcrops in rock crevices and clumps of moss or in flat exposed places in short rocky grassland on mountain tops, in rich black soil.

#### Diagnostic characters.

*Aloe saundersiae* can be distinguished from other grass aloes in KwaZulu-Natal with unkeeled leaves that are usually narrower than 3.5 cm and that lack a bulb-like underground swelling (*Aloe dominella*, *Aloe linearifolia*, *Aloe micracantha*, *Aloe minima*, *Aloe nicholsii* and *Aloe parviflora*), by its rosulate, spreading to recurved leaves (4–10 × 0.3 cm) that are without spots or with a few white spots near the base on the lower surface. The unbranched inflorescences (0.14–0.18 m high) have dense, capitate racemes of small, pale pinkish flowers (9–12 mm long), with the mouth slightly upturned and with spreading tips, but not bilabiate. Pedicels are 8–10 mm long. Rosettes are solitary or in small tufted groups.

#### Conservation status.

Endangered. Threats include overgrazing and too frequent fires (Raimondo et al. 2009, L. von Staden pers. comm.).

#### Distribution.

**Figure 38.**
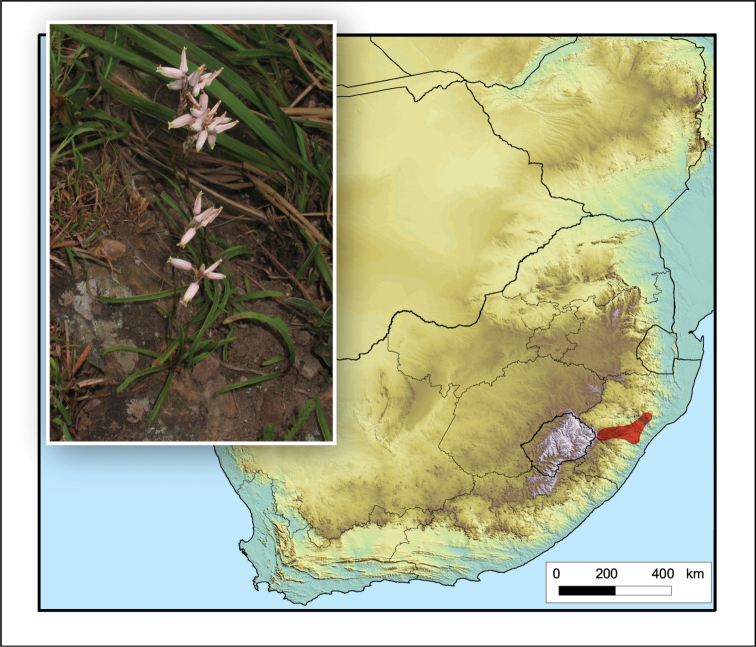
*Aloe saundersiae*. Photo: N.R. Crouch.

Only known from the central parts of KwaZulu-Natal, South Africa (Fig. 38).

### ^NE^ Aloe sharoniae

Taxon classificationPlantaeAsparagalesAsphodelaceae

N.R.Crouch & Gideon F.Sm.

C1CA307A-A31C-5976-A1F4-883A0AA8B1E8

#### Syn.

Aloe cooperi subsp. pulchra Glen & D.S.Hardy.

#### Common names.

Sharon’s grass aloe (English); sharonse-grasaalwyn (Afrikaans)

#### Description.

Grass aloe, 0.30–0.65 m high. Acaulescent plants or ***stem*** up to 0.15 m, erect, usually solitary, rarely with offshoots at ground level to form small groups, dried leaves not persistent. ***Leaves*** distichous, semi-erect to decurved, deciduous, green, usually without spots on upper surface, with copious white tubercles each bearing a hair-like process at base on lower surface, obscurely lineate, narrowly attenuate, 30–44(–82) cm long, 1.6–2.6(–3.9) cm wide at base, distinctly keeled, strongly V-shaped in cross section; margin narrow, white, cartilaginous, with rubbery, hair-like, ivory to greenish-white teeth in basal quarter to third only, 3–5 mm long, 1–2 mm apart at mid-leaf; exudate clear, drying clear. ***Inflorescences*** 0.33–0.59 m high, erect, simple. ***Raceme*** capitate to slightly elongate, 3.0–9.5 cm long, 7.5–9.5 cm wide, dense. ***Floral bracts*** 23–30 mm long, 6–8 mm wide, clasping the pedicel. ***Pedicels*** 33–43 mm long. ***Flowers***: *perianth* bright orange-red, yellowish-brown to purplish-brown tipped, 25–35 mm long, 6–8 mm across ovary, narrowing towards mouth, roundly trigonous, basally stipitate and narrowing into pedicel; outer segments free almost to base; *stamens* not or very slightly exserted; *style* only slightly exserted.

#### Flowering time.

February–March.

#### Habitat.

Open grassland on all slope aspects.

#### Diagnostic characters.

*Aloe sharoniae* is distinguished from other grass aloes in KwaZulu-Natal with strongly keeled leaves (*Aloe cooperi* and *Aloe myriacantha*) by the distichous leaves (30–44 cm long) that have no marginal teeth in the upper ⅔ and that are basally covered with white tuberculate maculations on the lower surface. It is further characterised by the floral bracts that clasp the pedicels (not flat as in *Aloe cooperi*). The inflorescence (0.33–0.59 m) is longer than the leaves. Flowers are bright orange-red, yellowish-brown to purplish-brown tipped and 25–35 mm long, with the mouth not bilabiate or upturned.

#### Conservation status.

Least Concern (Von Staden 2014b).

#### Distribution.

**Figure 39.**
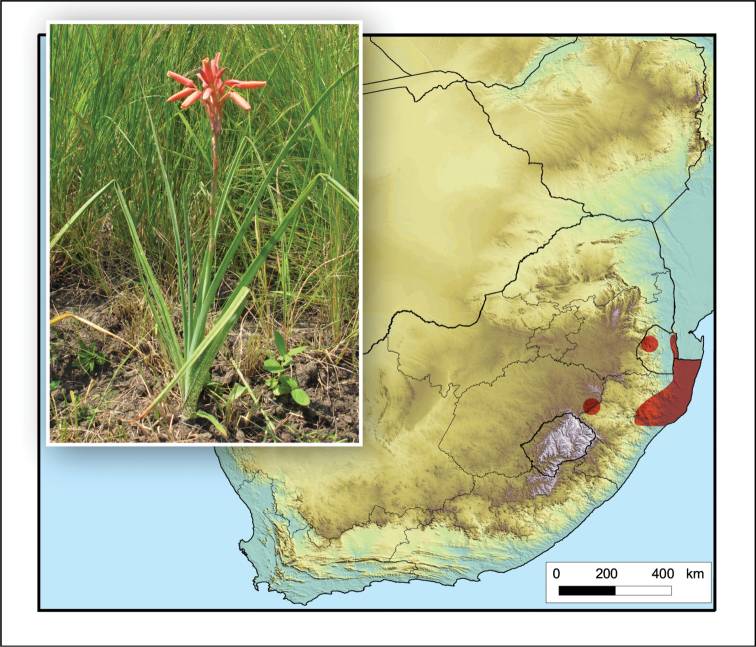
*Aloe sharoniae*. Photo: N.R. Crouch.

Sparse. Limited to KwaZulu-Natal, South Africa and Eswatini, although this species may also occur in southern Mozambique (Fig. 39).

### ^E^ Aloe spectabilis

Taxon classificationPlantaeAsparagalesAsphodelaceae

Reynolds

3CCDD0B3-585F-5AF6-8EAB-73E806B52063

#### Common names.

Natal aloe (English); natalaalwyn (Afrikaans); umhlaba (Zulu).

#### Description.

Solitary, arborescent plant up to 5 m high. ***Stem*** simple, rarely forked high up, erect, up to 4 m high, densely covered with persistent dried leaves. ***Leaves*** densely rosulate, suberect to spreading and eventually pendent, dull green, sometimes reddish tinged, without spots, usually with copious spines on both leaf surfaces, lanceolate-ensiform, ± 100 cm long, 12–15 cm wide at base; margin with stout, pungent, reddish to brownish teeth, 5–7 mm long, 10–20 mm apart; exudate honey-coloured. ***Inflorescence*** erect, much-branched, lower branches rebranched. ***Racemes*** cylindrical, rather truncate, ± 25 cm long, 9–10 cm wide, erect to suberect, dense. ***Floral bracts*** 4–5 mm long, 5 mm wide. ***Pedicels*** ± 3 mm long. ***Flowers***: *perianth* yellow to golden-yellow, buds with slightly redder tinge, ± 32 mm long, ± 5 mm wide across ovary, enlarging above ovary, narrowing towards mouth, slightly decurved; outer segments free for ± 15 mm; *stamens* and *style* exserted 20 mm.

#### Flowering time.

June–August.

#### Habitat.

Wide variety of habitats, including rocky places and open situations in grassland and savannah on hills.

#### Diagnostic characters.

*Aloe spectabilis* differs from the other tall often single-stemmed aloes in KwaZulu-Natal (*Aloe candelabrum*, *Aloe marlothii*, *Aloe pluridens*, *Aloe rupestris* and *Aloe thraskii*) with branched inflorescences, by having large (± 100 × 12–15 cm), suberect to spreading, eventually pendent leaves that usually have copious spines on both surfaces and pungent, reddish to brownish marginal teeth. The inflorescence is much-branched and rebranched with erect to suberect, very dense, cylindrical, rather truncate racemes of ± 25 cm long. Flowers are golden-yellow to reddish tinged and ± 32 mm long with the inner segment tips dull to glossy deep purplish-black to black and the exserted portion of the stamens orange (not with purplish segment tips and deep purple filaments as in *Aloe marlothii*).

#### Conservation status.

Least Concern (Raimondo et al. 2009).

#### Distribution.

**Figure 40.**
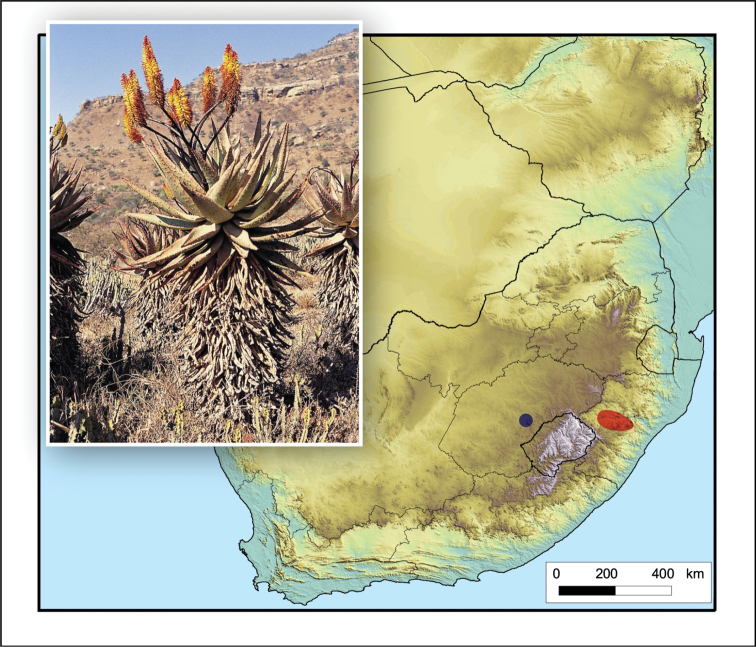
*Aloe spectabilis*. Photo: G.F. Smith.

Occurs in a small area in west-central KwaZulu-Natal, South Africa. Plants on the farm Bester Schrik, Winburg, Free State, South Africa, 5 km north of the Korannaberg, are a naturalised population (blue on map; Fig. 40) (For more details see Klopper et al. 2010).

### Aloe spicata

Taxon classificationPlantaeAsparagalesAsphodelaceae

L.f.

C33B5EEB-2653-5E4D-966F-0A1B3CB91E92

#### Syn.

*Aloe sessiliflora* Pole Evans.

#### Common names.

Lebombo aloe (English); Lebombo-aalwyn (Afrikaans); inhlaba (Zulu).

#### Description.

Acaulescent to arborescent or shrubby plants. ***Stem*** 1–2 m high, unbranched or branched low down or high up, erect to decumbent, with persistent dried leaves. ***Leaves*** densely rosulate, spreading to slightly recurved, green to sometimes almost entirely reddish on upper surface, without markings, lanceolate-attenuate, canaliculate, 50–80 cm long, 7–10 cm wide; margin reddish, with small deep pink to reddish teeth, 1–2 mm long, 8–15 mm apart; exudate pale honey-coloured. ***Inflorescence*** 0.6–1.2 m high, erect, simple. ***Raceme*** cylindrical, 30–50 cm long, 4–5 cm diameter, very dense. ***Floral bracts*** ± 10 mm long, 6 mm wide. ***Pedicels*** absent. ***Flowers***: *perianth* buds pale brownish-red, greenish-yellow when mature, 14–15 mm long, 5–6 mm across ovary, widening towards wide open mouth, campanulate; outer segments free to base; *stamens* exserted to 10 mm; *style* exserted 10–12 mm.

#### Flowering time.

June–August.

#### Habitat.

Wide variety of soils and habitats, including steep rocky slopes and cliffs. It is never found in exposed situations in deep soil.

#### Diagnostic characters.

*Aloe spicata* is one of only two aloes indigenous to KwaZulu-Natal that have sessile campanulate flowers with dark nectar in a simple inflorescence. It differs from *Aloe vryheidensis* in often being acaulescent in KwaZulu-Natal or sometimes having erect to decumbent stems (not procumbent), spreading to recurved leaves (not erect) and an erect inflorescence (not oblique). Racemes of *Aloe spicata* are narrow (4–5 cm wide) with greenish-yellow flowers (not pinkish-brown). The ovary is uniformly green (without red lines).

#### Conservation status.

Least Concern (Raimondo et al. 2009).

#### Distribution.

**Figure 41.**
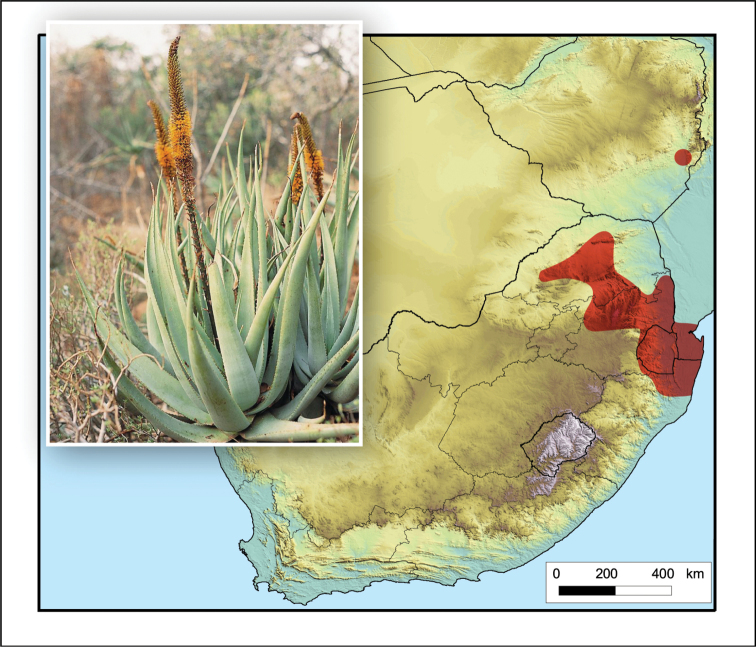
*Aloe spicata*. Photo: G.F. Smith.

Northern KwaZulu-Natal, Mpumalanga and Limpopo in South Africa, also throughout Eswatini and in southern Mozambique, with an isolated record in Zimbabwe (Fig. 41).

### Aloe suffulta

Taxon classificationPlantaeAsparagalesAsphodelaceae

Reynolds

6BBF09A7-B742-5954-969D-2E1794E4B1D8

#### Common names.

Sand aloe, climbing flower aloe (English); sandaalwyn (Afrikaans).

#### Description.

Shortly caulescent plants; rosettes solitary or sometimes suckering to form dense groups. ***Stem*** 0.1–0.2 m long, decumbent, with persistent dried leaves. ***Leaves*** loosely rosulate to cauline dispersed, spreading and recurved, dark glossy green, with dull white spots, spots sometimes irregularly scattered, usually more or less arranged in series of interrupted undulating transverse bands, larger and more confluent on lower surface, lanceolate-attenuate, 40–50 cm long, 2.5–4.0 cm wide at base; sheath 5–10 mm long, striatulate, light green, not auriculate; margin with whitish teeth, 1–2 mm long, 5–10 mm apart; exudate pale honey-coloured. ***Inflorescence*** 1–2 m high, supported by shrubs, 5- to 9-branched. ***Racemes*** cylindrical, slightly acuminate, terminal ± 15 cm long, lateral ± 8 cm long, lax. ***Floral bracts*** 4–6 mm long, 2–4 mm wide. ***Pedicels*** 7–10 mm long. ***Flowers***: *perianth* salmon-pink, whitish at mouth, 25–35 mm long, ± 6 mm across ovary, slightly narrowed above ovary, enlarging towards wide-open mouth, slightly curved, cylindrical-trigonous; outer segments free for 7 mm; *stamens* exserted to 6 mm; *style* exserted to 8 mm.

#### Flowering time.

June–July.

#### Habitat.

Under bushes, in sand with loose humus, on heavy black clay soils or on termite mounds, in very hot places. Very susceptible to cold.

#### Diagnostic characters.

*Aloe suffulta* can be distinguished from other maculate aloes in KwaZulu-Natal (*Aloe dewetii*, Aloe maculata subsp. maculata, *Aloe mudenensis*, *Aloe parvibracteata*, *Aloe prinslooi*, *Aloe pruinosa*, *Aloe umfoloziensis*, *Aloe vanrooyenii* and *Aloe viridiana*) by the 5- to 9-branched, climbing inflorescence (1–2 m high) with its very slender peduncle (7–9 mm diameter) that is supported by surrounding bushes. It is further characterised by the spreading and recurved, green deeply channelled leaves (40–50 × 2.5–4.0 cm) that are laxly rosulate to cauline dispersed (with striatulate sheaths) and are spotted on both surfaces, with marginal teeth 1–2 mm long. Flowers are salmon-pink, 25–35 mm long and lack the distinctive globose basal swelling of the maculate aloes. Pedicels are 7–10 mm long.

#### Conservation status.

Least Concern (Raimondo et al. 2009).

#### Distribution.

**Figure 42.**
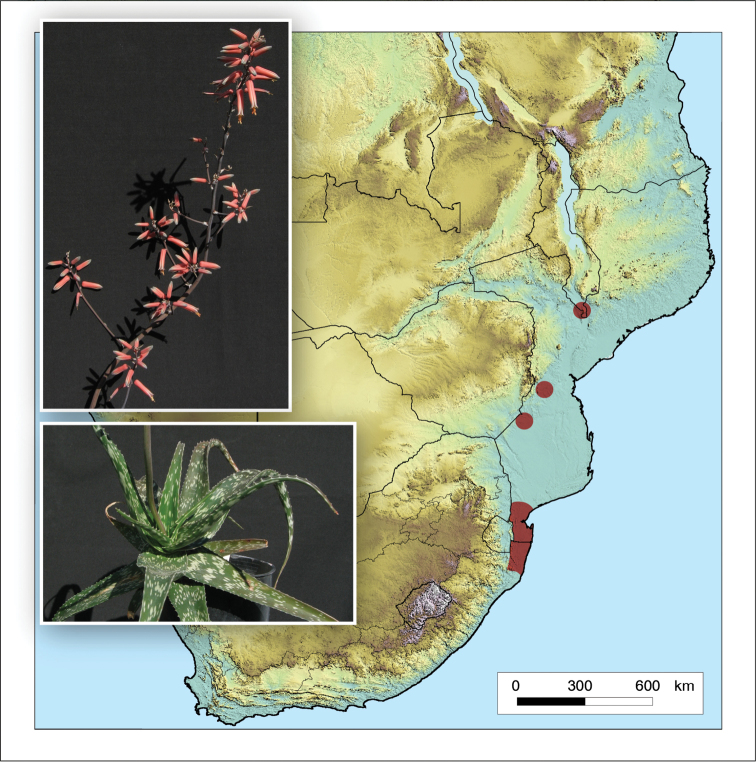
*Aloe suffulta*. Photos: J.B. Miller.

Widespread but infrequent, from northern KwaZulu-Natal, South Africa, through the coastal plains of southern Mozambique, to south-eastern Zimbabwe and southern Malawi (Fig. 42).

### ^NE^ Aloe suprafoliata

Taxon classificationPlantaeAsparagalesAsphodelaceae

Pole-Evans

9EA428D0-96C8-5D3B-8548-893C1723CC01

#### Common names.

Book aloe (English); boekaalwyn (Afrikaans); icena, umhlabandlazi (Zulu).

#### Description.

Acaulescent plants or rarely with short ***stem***, up to 0.5 m high, erect or procumbent; rosettes solitary, sometimes in small groups. ***Leaves*** distichous in young plants becoming densely rosulate, widely spreading to recurved, bluish-green to bluish-grey, more milky bluish-grey on lower surface, turning reddish-brown near apex, unspotted, obscurely lineate, texture smooth, lanceolate-acuminate, 30–40 cm long, 5–7 cm wide, with ± 8 cm dried twisted apex; margin with deltoid, sometimes bifid, reddish-brown teeth, 2–5 mm long, 5–10 mm apart; exudate clear. ***Inflorescence*** 0.6–2.0 m high, erect, simple. ***Raceme*** conical to cylindrical-acuminate, up to 25 cm long, 10 cm wide, rather dense. ***Floral bracts*** 15–20 mm long, 9–13 mm wide. ***Pedicels*** 14–20 mm long. ***Flowers***: *perianth* red, blue-grey tipped in bud, becoming rose-pink to scarlet red, greenish tipped, with a bloom, 33–50 mm long, 6–7 mm across ovary and throughout, cylindrical-trigonous, straight; outer segments free to base; *stamens* not or very slightly exserted; *style* exserted 1–2 mm.

#### Flowering time.

May–July.

#### Habitat.

Usually occurs in cracks in rocks or near sheer cliffs, along or near top of mountains, on rocks or rocky slopes in montane grassland or in places where soil is virtually absent or too thin to support other vegetation. Most localities receive frequent mist.

#### Diagnostic characters.

*Aloe suprafoliata* can be distinguished from other virtually acaulescent, non-maculate aloes in KwaZulu-Natal (*Aristaloe aristata*, Aloe chabaudii var. chabaudii, *Aloe gerstneri*, *Aloe pratensis*, Aloe reitzii var. vernalis and *Aloe vanbalenii*) by usually having solitary rosettes with leaves always distichous in young plants, becoming densely rosulate. Although other aloes also have distichous leaves when juveniles, this character persists for longer in *A. suprafoliata*. It is further characterised by having widely spreading to recurved, bluish-green to bluish-grey leaves (30–40 × 5–7 cm) with rather pungent marginal teeth. The inflorescence is erect, 0.6–2.0 m high and simple. The narrow racemes (up to 25 × 10 cm) have a silvery sheen with the flower buds hidden by large rounded silvery green floral bracts (15–20 mm long). Pedicels are erect (14–20 mm). Flowers are rose-pink to scarlet-red, up to 50 mm long and pencil-shaped.

#### Conservation status.

Least Concern (Raimondo et al. 2009).

#### Distribution.

**Figure 43.**
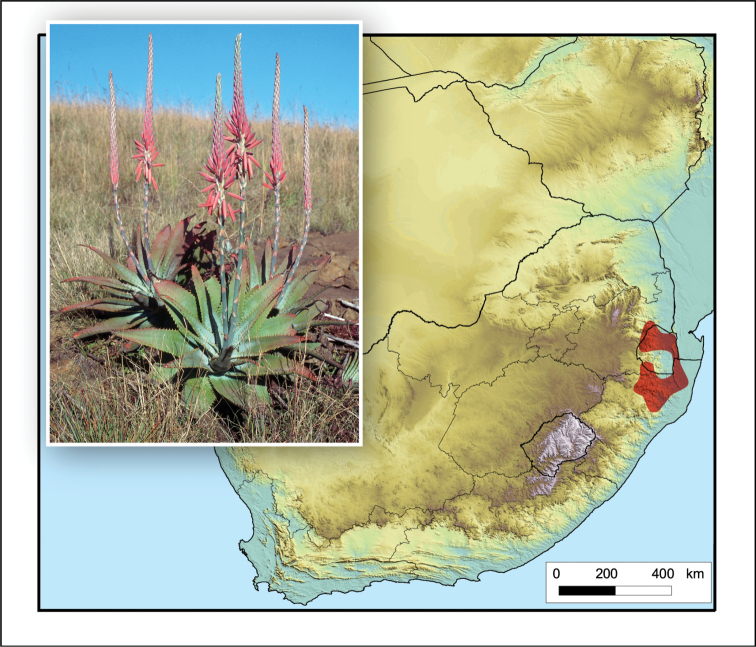
*Aloe suprafoliata*. Photo: N.R. Crouch.

Northern KwaZulu-Natal and just into eastern Mpumalanga in South Africa, as well as Eswatini (Fig. 43).

### ^NE^ Aloe thraskii

Taxon classificationPlantaeAsparagalesAsphodelaceae

Baker

A6F40EA8-C75A-51E7-9FC0-BA96AED44D48

#### Common names.

Dune aloe, strand aloe (English); strandaalwyn (Afrikaans); umhlaba (Zulu).

#### Description.

Solitary, arborescent plant, up to 3 m high. ***Stem*** simple, erect, 1–2 m high, can be up to 4 m, with persistent dried leaves. ***Leaves*** condensed rosulate, gracefully recurved, dull green to glaucous, without lines or spots, lower surface sometimes with a few spines in median line, lanceolate-attenuate, 160 cm long, 22 cm wide at base; margin very thin, narrow, reddish or brownish-red, with small deltoid reddish teeth, ± 2 mm long, 10–20 mm apart; exudate honey-coloured. ***Inflorescence*** 0.5–0.8 m high, erect, 4- to 8-branched. ***Racemes*** broadly cylindrical, slightly acuminate, somewhat truncate, up to 25 cm long, usually shorter, very dense. ***Floral bracts*** 9 mm long, 6 mm wide. ***Pedicels*** 1–2 mm long. ***Flowers***: *perianth* greenish to orange in buds, lemon-yellow to pale orange when mature, greenish tipped, ± 25 mm long, ± 6 mm across ovary, enlarging towards throat, mouth constricted and upturned, cylindrical, slightly clavate; outer segments free for ± 17 mm; *stamens* exserted 15–20 mm; *style* exserted to 20 mm.

#### Flowering time.

June–July.

#### Habitat.

Beach dunes, in almost pure sand in low coastal vegetation or taller bush.

#### Diagnostic characters.

*Aloe thraskii* differs from the other tall, often single-stemmed aloes in KwaZulu-Natal (*Aloe candelabrum*, *Aloe marlothii*, *Aloe pluridens*, *Aloe rupestris* and *Aloe spectabilis*) with branched inflorescences, by its strictly coastal habitat and in having long (± 160 × 22 cm), strongly recurved, deeply channelled leaves with small reddish marginal teeth. The inflorescence is 4- to 8-branched with erect, very dense, broadly cylindrical, slightly acuminate and somewhat truncate racemes of up to 25 cm long. Flowers are lemon-yellow to pale orange and ± 25 mm long. The long-exserted yellowish-orange stamens and style emerge from the flower at an angle (not straight as in *Aloe rupestris*).

#### Conservation status.

Near-threatened. Threats include habitat loss owing to urban expansion along the coast, as well as illegal collecting (Raimondo et al. 2009).

#### Distribution.

**Figure 44.**
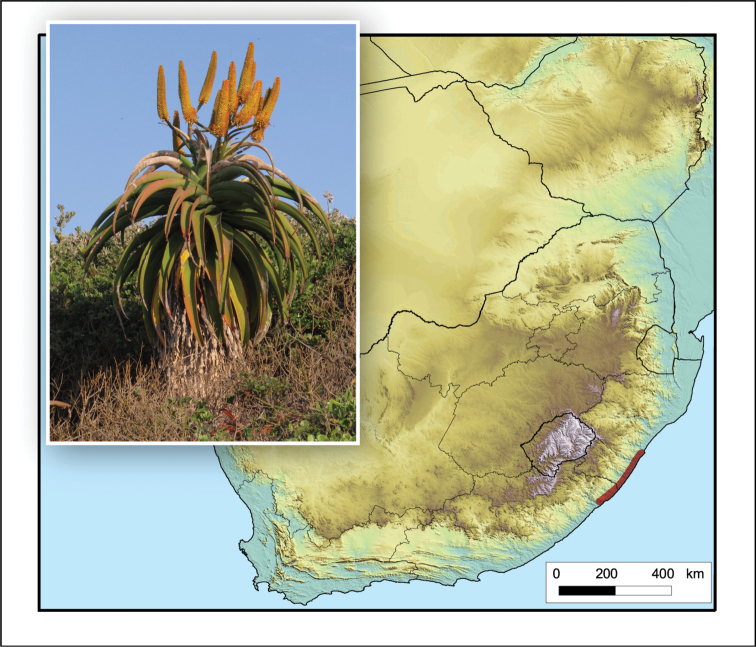
*Aloe thraskii*. Photo: G. Nichols.

Occurs in a narrow coastal strip from the far northern coast of the Eastern Cape into KwaZulu-Natal to just north of Durban, South Africa (Fig. 44).

### ^NE^ Aloe umfoloziensis

Taxon classificationPlantaeAsparagalesAsphodelaceae

Reynolds

BFBD1F2C-4AB3-5F05-87C6-F37C0A5A2A66

#### Common names.

Groot-bontaalwyn (Afrikaans); icena, ilicena (Zulu).

#### Description.

Acaulescent plant or with short ***stem***, up to 0.4 m high; rosettes sometimes solitary, usually suckering to form small groups; with persistent dried leaves. ***Leaves*** densely rosulate, spreading or deflexed, upper surface green to brownish-green, with numerous dull white oblong spots, irregularly scattered or sometimes in undulating interrupted transverse bands, lower surface paler green, without spots or obscurely to distinctly spotted, usually somewhat lineate, lanceolate-attenuate, up to 20–30 cm long, 8–9 cm wide, with dried twisted apex; margin with horny, pungent deltoid brown teeth, 3–5 mm long, 10–15 mm apart; exudate honey-coloured, drying purplish. ***Inflorescence*** 1.0–1.5 m high, erect, 5- to 8-branched from about middle or above, lowest branch sometimes rebranched. ***Racemes*** capitate, apex rounded, 7–9 cm long, 7–9 cm wide, rather dense. ***Floral bracts*** 8–12 mm long. ***Pedicels*** 10–15 mm long. ***Flowers***: *perianth* coral-red, 33–38 mm long, 8–9 mm across ovary, abruptly constricted above ovary to form subglobose basal swelling, widening towards wide-open mouth, slightly decurved, laterally compressed; outer segments free for 8–9 mm; *stamens* and *style* exserted 3–5 mm.

#### Flowering time.

July–August.

#### Habitat.

Low-lying sub-tropical open savannah, open grassland and on rocky places for some distance along rivers and watercourses.

#### Diagnostic characters.

*Aloe umfoloziensis* can be distinguished from other maculate aloes in KwaZulu-Natal (*Aloe dewetii*, Aloe maculata subsp. maculata, *Aloe mudenensis*, *Aloe parvibracteata*, *Aloe prinslooi*, *Aloe pruinosa*, *Aloe suffulta*, *Aloe vanrooyenii* and *Aloe viridiana*) by the tall, 5- to 8-branched, sometimes rebranched, inflorescence (1.0–1.5 m high) with rather small round-topped capitate racemes (7–9 cm long and wide) and pedicels that are 10–15 mm long. Flowers are coral-red, 33–38 mm long and with a globose basal swelling (8–9 mm diameter). Leaves are spreading or deflexed, up to 20–30 × 8–9 cm and densely spotted on the upper surface, while the paler lower surface is without spots or obscurely to distinctly spotted, usually somewhat lineate. Marginal teeth are 3–5 mm long.

#### Conservation status.

Near-threatened. Threats include habitat loss and degradation owing to trampling by livestock, erosion and agriculture (L. von Staden pers. comm.).

#### Distribution.

**Figure 45.**
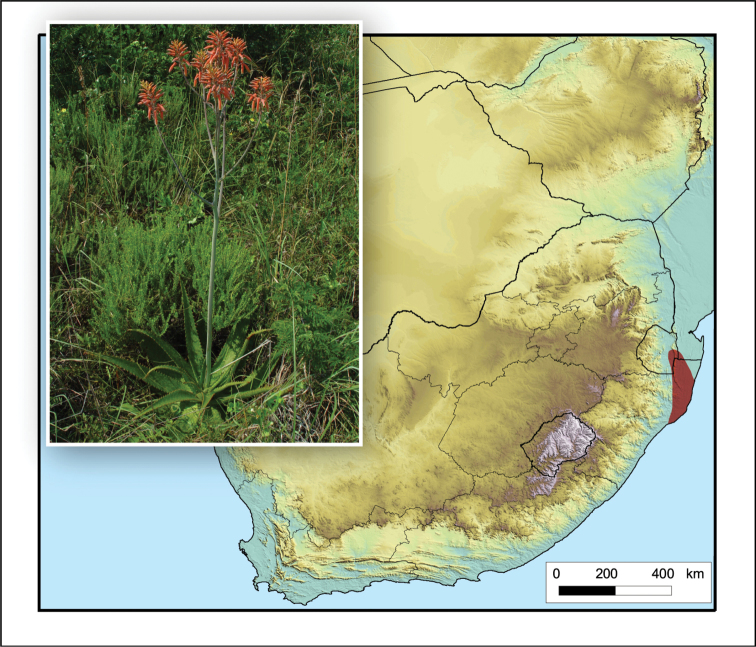
*Aloe umfoloziensis*. Photo: G. Nichols.

Northern KwaZulu-Natal in South Africa, as well as south-eastern Eswatini and just entering southern Mozambique (Fig. 45).

### ^NE^ Aloe vanbalenii

Taxon classificationPlantaeAsparagalesAsphodelaceae

Pillans

72335E46-B9CC-5DF8-895C-B3F543CA7A11

#### Common names.

Van Balen’s aloe (English); rooiblaaraalwyn (Afrikaans); icenalamatshe, incenalendlovu, icenandhlovu, inhlahlwane (Zulu).

#### Description.

Acaulescent plants or ***stem*** very short, 0.2–0.3 m tall, branching at top and base; rosettes suckering to form dense groups, erect. ***Leaves*** densely rosulate, spreading to strongly decurved, green to copper red, greener on lower surface, usually obscurely lineate, lanceolate, long attenuate, deeply channelled, 50–80 cm long, 9–15 cm wide; margin somewhat horny, reddish to reddish-brown, with pungent, reddish, deltoid teeth, 3–5 mm long, 10–15 mm apart; exudate pale honey-coloured. ***Inflorescence*** ± 1 m high, erect, 2- or 3-branched from about middle. ***Racemes*** narrowly conical, up to 25–30 cm long, 8–10 cm wide, rather dense. ***Floral bracts*** up to 15 mm long, 6–7 mm wide. ***Pedicels*** 14–23 mm long. ***Flowers***: *perianth* orange-yellow or sometimes dull reddish-pink in bud, usually buff-yellow or sometimes dull red when mature, 30–40 mm long, 6–7 mm across ovary, widening slightly towards wide-open mouth, straight, cylindrical-trigonous, slightly laterally compressed; outer segments free to base; *stamens* exserted to 10 mm; *style* exserted 10–12 mm.

#### Flowering time.

June–July.

#### Habitat.

Flat rocks and rocky outcrops with minimal soil in Nkonkoni Veld and Zululand Thornveld. Frost-free area with moderately high summer rainfall.

#### Diagnostic characters.

*Aloe vanbalenii* can be distinguished from other virtually acaulescent, non-maculate aloes in KwaZulu-Natal (*Aristaloe aristata*, Aloe chabaudii var. chabaudii, *Aloe gerstneri*, *Aloe pratensis*, Aloe reitzii var. vernalis and *Aloe suprafoliata*) by its suckering habit that forms dense groups of rosettes. It is further characterised by its much recurved, green to copper red, broad (50–80 × 9–15 cm), deeply-channelled leaves with pungent marginal teeth. The inflorescence is erect, ± 1 m high and 2- or 3-branched. Floral bracts are long (up to 15 mm) and pedicels erect (14–23 mm long). Flowers are orange-yellow or reddish-pink, 30–40 mm long and not narrowed above the ovary. Leaves have a characteristic cinnamon or musty smell when damaged (Carter et al. 2011).

#### Conservation status.

Least Concern (Raimondo et al. 2009).

#### Distribution.

**Figure 46.**
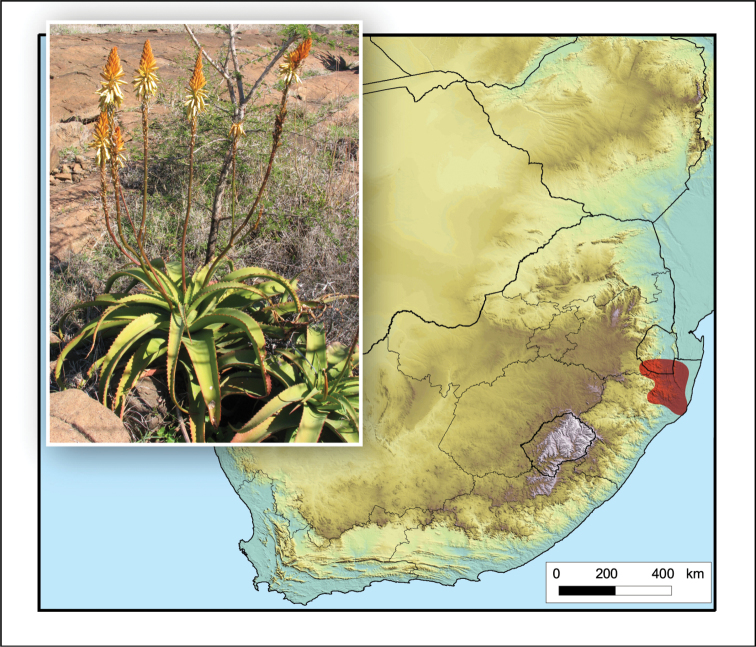
*Aloe vanbalenii*. Photo: N.R. Crouch.

Confined to the Lebombo Mountain range in northern KwaZulu-Natal, South Africa and southern Eswatini (Fig. 46).

### ^E^ Aloe vanrooyenii

Taxon classificationPlantaeAsparagalesAsphodelaceae

Gideon F.Sm. & N.R.Crouch

F668A354-F4E0-5901-8DFC-A864F3F565F3

#### Common names.

Van Rooyen’s aloe (English); vanrooyense-aalwyn (Afrikaans).

#### Description.

Acaulescent plants; rosettes solitary, very rarely suckering to form small groups; with persistent dried leaves. ***Leaves*** densely rosulate, distinctly spreading, reflexed, upper surface shiny pale green, with pale milky green to whitish, variously shaped spots, often more or less confluent in transverse bands, lower surface uniformly milky green, rarely with longitudinal darker greenish striations, deltoid-lanceolate, attenuate, 12–15 cm long, 6–8 cm wide, apex dry, sometimes with small teeth at keel near apex; margins whitish, near-translucent, with very pungent, brownish-orange, recurved teeth, 3–4 mm long, 3–4 mm apart; exudate clear, drying purplish. ***Inflorescence*** 0.5–0.8 m high, erect, 1- or 2-branched below middle. ***Racemes*** cylindrical to slightly conical, 25–47 cm long, 7–9 cm wide, lax. ***Floral bracts*** 8–10 mm long. ***Pedicels*** 8–10 mm long. ***Flowers***: *perianth* orange or red, 33–38 mm long, 8–10 mm across ovary, abruptly constricted above ovary to form globose basal swelling, widening towards wide open mouth, cylindrical-trigonous; outer segments free for 8–15 mm; *stamens* exserted up to 3 mm; *style* slightly exserted.

#### Flowering time.

October–November.

#### Habitat.

Thornveld savannah.

#### Diagnostic characters.

*Aloe vanrooyenii* can be distinguished from other maculate aloes in KwaZulu-Natal (*Aloe dewetii*, Aloe maculata subsp. maculata, *Aloe mudenensis*, *Aloe parvibracteata*, *Aloe prinslooi*, *Aloe pruinosa*, *Aloe suffulta*, *Aloe vanrooyenii* and *Aloe viridiana*) by the 1- or 2-branched inflorescence with lax, cylindrical to slightly conical racemes (25–47 × 7–9 cm) with pedicels 8–10 mm long. It is further characterised by the very large, erect fruit (25–28 × 14–18 mm in fresh state), which cause the peduncle to bend towards the ground as it cannot support the weight of the large mature capsules. Flowers are orange or red, 33–38 mm long and with a globose basal swelling (8–10 mm diameter). Leaves are distinctly spreading and reflexed, 12–15 × 6–8 cm and spotted on the upper surface, while the lower surface is rarely lineate. Marginal teeth are 3–4 mm long. This is the only early summer-flowering maculate aloe in KwaZulu-Natal (Smith and Crouch 2006).

#### Conservation status.

Least Concern (Raimondo et al. 2009).

#### Distribution.

**Figure 47.**
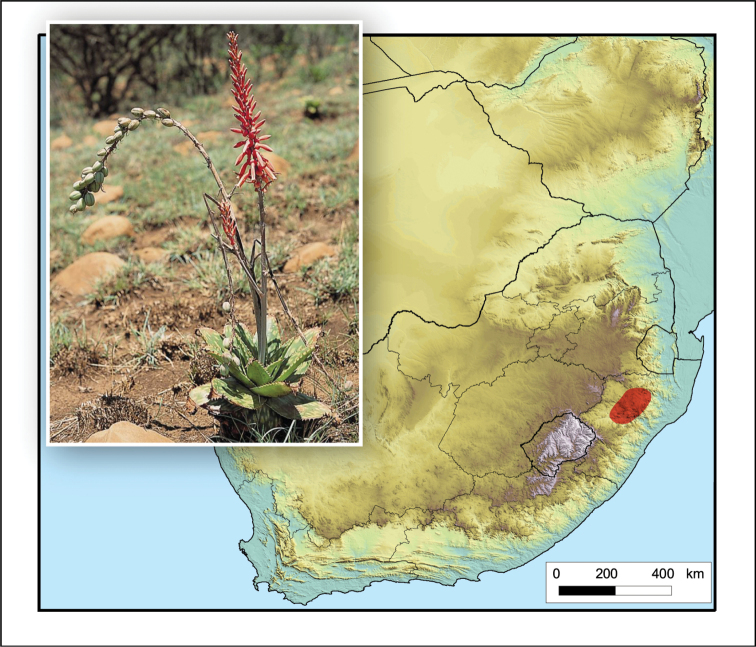
*Aloe vanrooyenii*. Photo: G.F. Smith.

Known from the region linking Ladysmith, Newcastle and Vryheid, KwaZulu-Natal, South Africa (Fig. 47).

### ^E^ Aloe viridiana

Taxon classificationPlantaeAsparagalesAsphodelaceae

Gideon F.Sm. & Figueiredo

E85C9979-E4FC-58B2-A6BE-4FE851804E0E

#### Syn.

*Aloe greenii* Baker, nom. illegit.

#### Common names.

Green's aloe (English); groenaalwyn (Afrikaans); icena (isiZulu).

#### Description.

Acaulescent plants; rosettes suckering to form large dense groups, erect, 0.15–0.25 m high. ***Leaves*** densely rosulate, suberect to spreading-recurved, bright green, obscurely lineate, with many confluent oblong white spots forming irregular transverse wavy bands, bands broader and more pronounced on lower surface, broadly linear-lanceolate, gradually attenuate, ± 40–50 cm long, 7–8 cm wide; margin with deltoid, pale brown to pink teeth, 3–4 mm long, 8–10 mm apart; exudate clear, drying yellow. ***Inflorescence*** ± 1.0–1.3 m high, erect, 5- to 7-branched from above middle, lower branches sometimes rebranched. ***Racemes*** oblong-cylindrical, 15–25 cm long, rather dense. ***Floral bracts*** ± 10 mm long, 2–3 mm wide. ***Pedicels*** 7–10 mm long. ***Flowers***: *perianth* light to dark flesh pink, with powdery bloom, 28–30 mm long, ± 7 mm across ovary, abruptly constricted above ovary to form globose basal swelling, widening towards mouth, slightly decurved; outer segments free for 7–10 mm; *stamens* exserted 1–2 mm; *style* exserted 2–4 mm.

#### Flowering time.

January–March.

#### Habitat.

On stony soil, in low-lying flat sandy areas, often in deep shade or semi-shade in dry thorny woodland.

#### Diagnostic characters.

*Aloe viridiana* can be distinguished from other maculate aloes in KwaZulu-Natal (*Aloe dewetii*, Aloe maculata subsp. maculata, *Aloe mudenensis*, *Aloe parvibracteata*, *Aloe prinslooi*, *Aloe pruinosa*, *Aloe suffulta*, *Aloe umfoloziensis* and *Aloe vanrooyenii*) by the rosettes that sucker profusely to form large groups. It is further characterised by the recurved leaves (± 40–50 × 7–8 cm), with spots on both surfaces, but with the markings more pronounced on the lower surface. The 5- to 7-branched inflorescence (± 1.0–1.3 m high), that is without a grey bloom, has oblong-cylindrical (15–25 cm long), rather dense racemes, with pedicels 7–10 mm long. Flowers are light to dark flesh pink, with a powdery bloom, 28–30 mm long and with a globose basal swelling (± 7 mm diameter).

#### Conservation status.

Least Concern (Raimondo et al. 2009).

#### Distribution.

**Figure 48.**
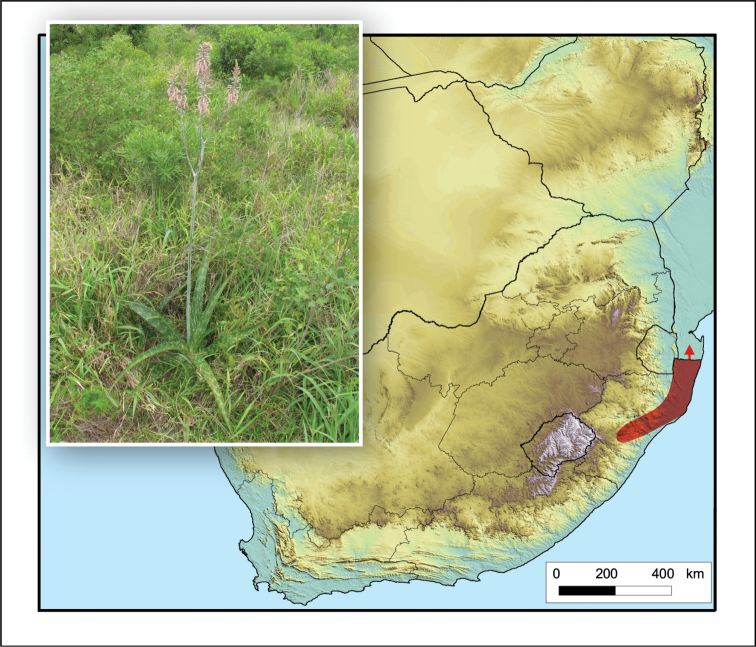
*Aloe viridiana*. Photo: N.R. Crouch.

Fairly widespread, but uncommon, in eastern KwaZulu-Natal, South Africa. Possibly also in southern Mozambique (Fig. 48). This aloe is not encountered in large numbers where it occurs in the wild.

#### Notes.

This aloe has been known under the name *Aloe greenii* Baker. However, this validly published name (Baker 1880) is a later illegitimate homonym, as the combination was earlier published as *Aloe greenii* Green ex Rob. in 1875. The name published by Robinson (1875) cannot, with certainty, be applied to any known maculate aloe owing to the very short descriptive text accompanying the name; however, it was nonetheless validly published. Smith and Figueiredo (2018) provided the necessary replacement name, *Aloe viridiana*, for material of this KwaZulu-Natal aloe, so providing nomenclatural certainty for material thus far known as *A. greenii* Baker.

### Aloe vryheidensis

Taxon classificationPlantaeAsparagalesAsphodelaceae

Groenew.

73DE1315-5269-5218-9CA2-9AB28D4EC7B4

#### Common names.

Dolomite aloe, Vryheid aloe (English); bruinaalwyn (Afrikaans).

#### Description.

Acaulescent or arborescent plants or ***stem*** up to 2 m high, procumbent or shortly suberect, unbranched or sometimes branched at base; with persistent dried leaves; rosettes solitary or in small groups. ***Leaves*** densely rosulate, arcuate-erect to slightly spreading and recurved, glaucous green to dark green with bluish or reddish tinge, without spots, texture smooth, lanceolate-attenuate, 40–80 cm long, 9–13 cm wide at base; margin red, subcorneous, with pungent, deltoid, straight, reddish teeth, 2–3 mm long, 10–15 mm apart; exudate drying yellow. ***Inflorescences*** 0.6–1.5 m high, oblique to erect, simple. ***Raceme*** cylindrical, 30–40 cm long, 5–7 cm wide, erect, very dense. ***Floral bracts*** 8–15 mm long, 5–10 mm wide. ***Pedicels*** absent. ***Flowers***: *perianth* greenish-yellow to reddish in bud, rose-coloured or greenish-yellow to yellowish when mature, 8–20 mm long, 4–5 mm across ovary, widening to wide open mouth, campanulate-cylindrical; outer segments free to base; *stamens* exserted 6–15 mm; *style* exserted 7–17 mm.

#### Flowering time.

July–August.

#### Habitat.

Usually on alkaline soils derived from shale or dolomite (on sandstone at Vryheid).

#### Diagnostic characters.

*Aloe vryheidensis* is one of only two aloes indigenous to KwaZulu-Natal that have sessile campanulate flowers with dark nectar in a simple inflorescence. It differs from *Aloe spicata* in having procumbent to shortly sub-erect stems (not erect) that are sometimes absent, arcuate-erect to slightly spreading leaves (not recurved) and an inflorescence with the peduncle oblique (not erect), then bent upwards directly below the erect raceme. The racemes of *Aloe vryheidensis* are narrow (5–7 cm wide), but slightly wider than those of *Aloe spicata*, with pinkish-brown flowers (not greenish-yellow). The ovary has red lines longitudinally down the 3 broad angles.

#### Conservation status.

Least Concern (L. von Staden pers. comm.).

#### Distribution.

**Figure 49.**
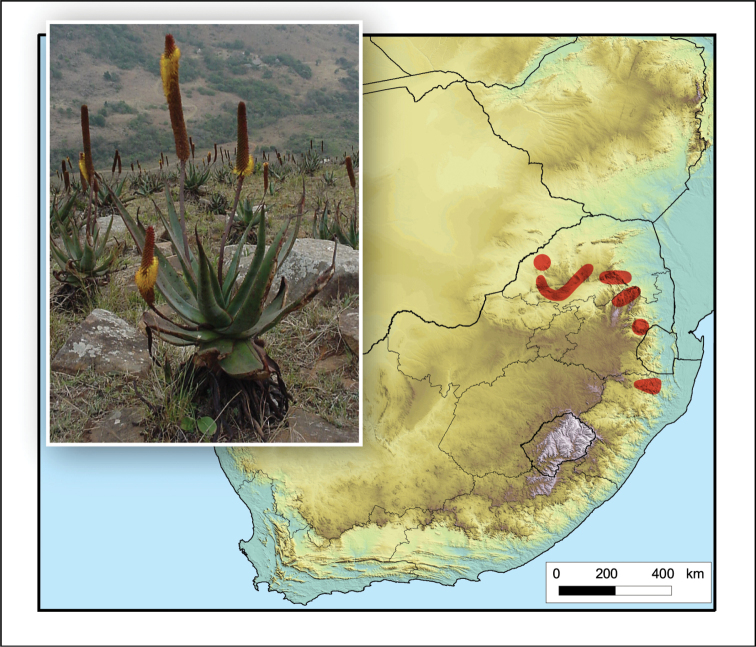
*Aloe vryheidensis*. Photo: G. Nichols.

Mountainous areas of northern KwaZulu-Natal, Mpumalanga and Limpopo, South Africa (Fig. 49).

## Supplementary Material

XML Treatment for Aloiampelos tenuior

XML Treatment for Aloidendron barberae

XML Treatment for Aloidendron tongaense

XML Treatment for Aristaloe aristata

XML Treatment for Aloe arborescens

XML Treatment for Aloe bergeriana

XML Treatment for Aloe boylei

XML Treatment for Aloe candelabrum

XML Treatment for Aloe chabaudii Schönland var. chabaudii

XML Treatment for Aloe cooperi

XML Treatment for Aloe dewetii

XML Treatment for Aloe dominella

XML Treatment for Aloe ecklonis

XML Treatment for Aloe gerstneri

XML Treatment for Aloe hlangapies

XML Treatment for Aloe inconspicua

XML Treatment for Aloe kniphofioides

XML Treatment for Aloe kraussii

XML Treatment for Aloe linearifolia

XML Treatment for Aloe maculata All. subsp. maculata

XML Treatment for Aloe marlothii

XML Treatment for Aloe micracantha

XML Treatment for Aloe minima

XML Treatment for Aloe modesta

XML Treatment for Aloe mudenensis

XML Treatment for Aloe myriacantha

XML Treatment for Aloe neilcrouchii

XML Treatment for Aloe nicholsii

XML Treatment for Aloe parvibracteata

XML Treatment for Aloe parviflora

XML Treatment for Aloe pluridens

XML Treatment for Aloe pratensis

XML Treatment for Aloe prinslooi

XML Treatment for Aloe pruinosa

XML Treatment for Aloe reitzii Reynolds var. vernalis

XML Treatment for Aloe rupestris

XML Treatment for Aloe saundersiae

XML Treatment for Aloe sharoniae

XML Treatment for Aloe spectabilis

XML Treatment for Aloe spicata

XML Treatment for Aloe suffulta

XML Treatment for Aloe suprafoliata

XML Treatment for Aloe thraskii

XML Treatment for Aloe umfoloziensis

XML Treatment for Aloe vanbalenii

XML Treatment for Aloe vanrooyenii

XML Treatment for Aloe viridiana

XML Treatment for Aloe vryheidensis
